# Functionalized azobenzenes for micellar solar thermal energy storage as a next-generation MOST system

**DOI:** 10.1038/s42004-025-01750-5

**Published:** 2025-11-24

**Authors:** Rui Huang, Alex S. Loch, Alice Pincham, Andrew J. Smith, Annela Seddon, Zhihang Wang, Dave J. Adams

**Affiliations:** 1 School of Chemistry, University of Glasgow, Glasgow, UK; 2 School of Physics, HH Wills Physics Laboratory, University of Bristol, Bristol, UK; 3 Diamond Light Source Ltd, Diamond House, Harwell Science and Innovation Campus, Didcot, UK; 4 School of Engineering, College of Science and Engineering, University of Derby, Derby, UK; 5 Department of Materials Science and Metallurgy, University of Cambridge, Cambridge, UK

**Keywords:** Light harvesting, Materials for energy and catalysis, Soft materials

## Abstract

Despite being the most abundant sustainable energy resource, solar energy still faces major challenges in efficient capture and long-term storage. Molecular Solar Thermal Energy Storage (MOST) systems address this issue by employing photoswitchable molecules that absorb sunlight and store energy through reversible isomerization, cyclization or other intramolecular rearrangements. Azobenzenes are attractive due to their well-characterized photoresponsive behavior; however, conventional systems are hindered by low energy density, limited energy storage duration, and a reliance on organic solvents. Here, we present the Micellar Solar Thermal Energy Storage system (MIST) approach based on micellar aggregates that operate effectively across aqueous dispersions and gel states. These systems exhibit progressively enhanced energy storage lifetimes with increasing degrees of self-assembly, while delivering competitive energy densities. The thermal stability arises from restricted molecular mobility within the self-assembled structures and is enhanced on gelation, extending the calculated thermal half-life of the *cis* isomer from 148 days in dimethyl sulfoxide (DMSO), to 233 days in water, and to 12.8 years in the gel state. Compared to previous azobenzene-based MOST systems, our MIST approach offers significantly extended energy storage durations and improved material processability, including water-compatible formulations and, macroscopic heat release in the gel state (up to 5.7 °C).

## Abstract (web summary)

Molecular solar thermal energy storage (MOST) systems employ photoswitchable molecules that absorb sunlight and store energy through reversible isomerization, cyclization or other intramolecular rearrangements. Here, the authors introduce a micellar solar thermal energy storage (MIST) system, utilizing micellar aggregates based on azobenzene-functionalized dipeptide amphiphiles to achieve extended energy storage durations, offering water-compatible formulations and improved processability for sustainable energy applications.

## Introduction

Molecular Solar Thermal Energy Storage (MOST) is a means of capturing and storing solar energy through chemical bonds^1–3^. The absorption of sunlight by photoswitchable molecules induces a transition from the ground state to a metastable, high-energy isomer. If the isomer remains stable over a measurable timescale before reverting to its original form, it can serve as an effective medium for energy storage. When energy is needed, back-conversion to the original form can be triggered (typically by heat^4^, catalyst^5,6^, electrical potential^3,7^, or, more efficiently, by direct irradiation with a different wavelength of light^8–11^) releasing the stored energy as latent heat. This process has the potential to allow energy to be stored without losses and retrieved on demand, offering an efficient and sustainable method for harnessing solar power.

Azobenzenes have been widely examined for MOST due to their ability to undergo reversible photoisomerization^12,13^. However, there are many reasons why this class of materials is generally not efficient^14^. For instance, the stability of the *cis* isomer over time is critical for long-term energy storage. However, in most azobenzene-based systems, thermal relaxation to the *trans* isomer occurs readily, especially under elevated temperatures, leading to significant energy loss over time. Therefore, minimizing undesired thermal back-conversion is essential for effective long-term energy storage^15^. From a practical perspective, the dependence on organic solvents further restricts scalability and real-world applicability, especially when integration with water-based heat transfer fluids is desired. Designing water-soluble azobenzenes, therefore, requires tailored molecular structures to ensure compatibility and functionality in aqueous environments.

As one promising strategy for modifying azobenzenes, the development of azobenzene-based gels has attracted considerable attention, with numerous examples reported in recent years^16–18^. For instance, dipeptide-functionalized azobenzenes have been shown to form gels that transform into solutions upon ultraviolet (UV) irradiation, driven by *trans*-to-cis isomerization of the azobenzene unit^19^. Gels were reformed on irradiation with visible light as the *trans* isomer is reformed. However, whether such systems are suitable for MOST applications remains unexplored.

Previously, we have shown that a range of related functionalized dipeptides can form micellar states at high pH where the carboxylic acid at the C-terminus is deprotonated^20,21^. The micellar state formed is controlled by the chemical structure of the functionalized dipeptide as well as the counterion and the presence of additives. Micellar aggregates can be formed at concentrations as high as 200 mg mL^-1^ and are stable for extended periods of time (on the timescale of years). For other azobenzene-based photoswitchable molecules, self-assembly into defined nanostructures has been shown to dramatically enhance the activation barrier to *cis*-to-*trans* isomerization^22^, ascribed to the non-covalent interactions (specifically hydrogen bonding and π − π stacking) between molecules in the aggregated state. Phase changes on photoisomerization have been shown to be effective in improving MOST systems^9,10,23,24^. With this in mind, we hypothesized that assembling a micellar phase from functionalized azobenzenes to construct a Micellar Solar Thermal Energy Storage (MIST) system could reduce the rate of cis-to-trans isomerization and enable the development of materials suited for the next generation of MOST technologies.

Here, we present a series of azobenzene-functionalized dipeptide amphiphiles designed to self-assemble into micellar structures in aqueous environments at high pH. The systems were engineered to introduce a strategy using self-assembled gels to extend the half-life of photoisomers. Notably, this gel-based approach has not been reported in the previously cited studies. Our MIST methodology does not conflict with established principles for prolonging the thermal lifetime of MOST systems; instead, it builds upon them by offering a different dimension of control. This approach provides a straightforward and efficient means of stabilizing photoisomers, with fewer synthetic demands compared to the development of new photoactive molecular frameworks. We demonstrate that the formation of distinct micellar morphologies, ranging from spherical to worm-like and elliptical cylinder aggregates, can be precisely modulated by the dipeptide sequence, as confirmed by comprehensive small-angle X-ray scattering (SAXS) measurements. Importantly, we further show that transitioning from an organic solution to micellar dispersion and ultimately to gel states, achieved through calcium-induced crosslinking, leads to a progressive enhancement of the energy storage lifetime, reaching up to 4674 days (12.8 years, extrapolated) in the gel state. For a practical demonstration of MIST functionality, we establish that these systems allow multiple photoswitching cycles with structural reversibility, and that macroscopic energy release can be effectively triggered by light in gel films for the first time. Together, these findings validate our hypothesis and offer a different design for next-generation energy storage systems that are water-processable, highly stable, and suitable for scalable solar thermal energy conversion and storage applications.

## Results and discussion

**Fig. 1:**
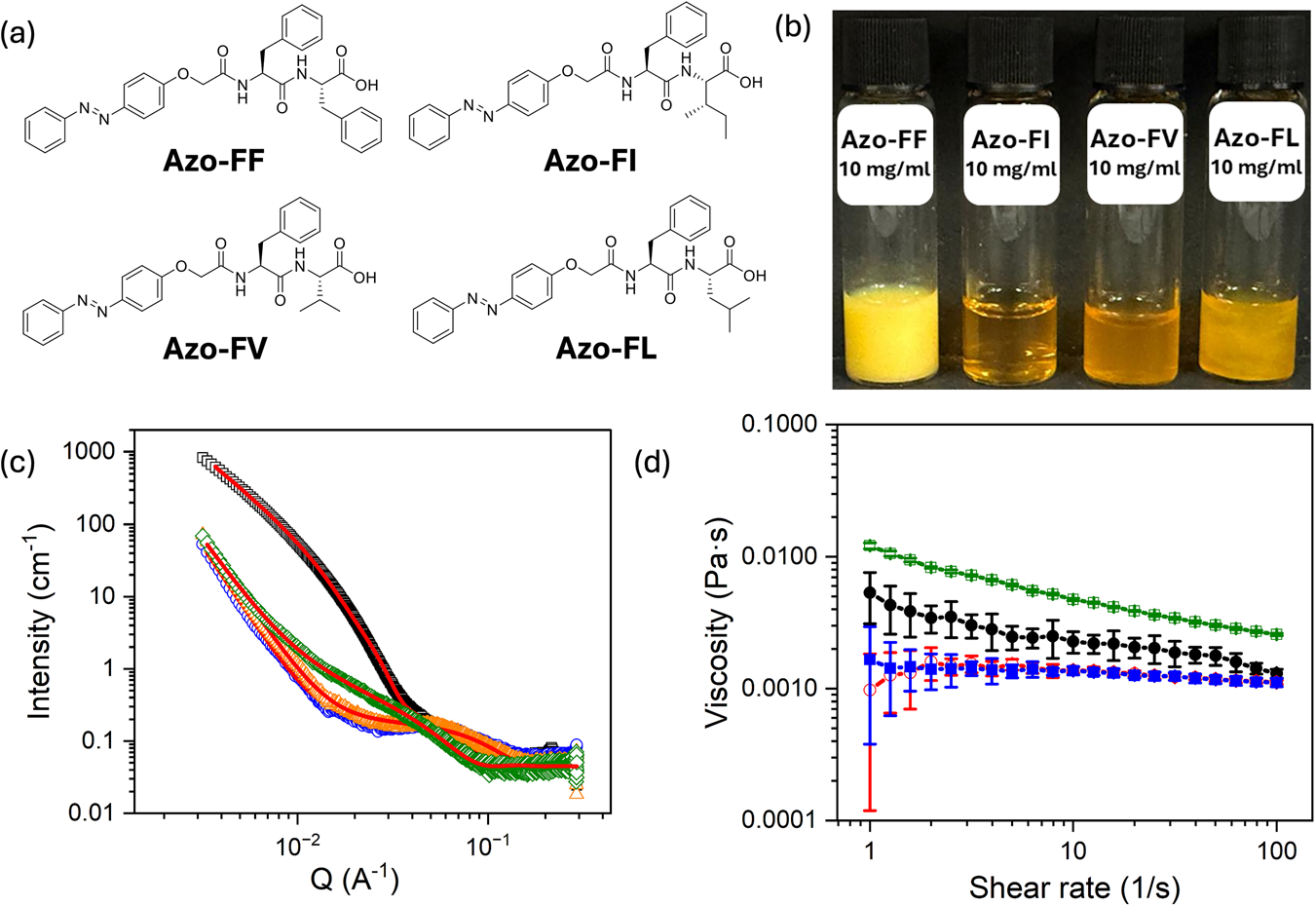
Compounds used here and characterization of micellar solutions formed from these. **a** Chemical structures of the four azobenzene-dipeptides used here. The amino acids used are named using one letter codes; **b** photographs of solutions formed from each azobenzene-dipeptide in water at pH 10.5 at a concentration of 10 mg mL^−1^; **c** SAXS data for each azobenzene-dipeptide in water at pH 10.5 at a concentration of 10 mg mL^−1^ (black, Azo-FF; blue, Azo-FI; orange, Azo-FV; green, Azo-FL) with the fits to the data shown as red lines; **d** viscosity data for each azobenzene-dipeptide in water at pH 10.5 at a concentration of 10 mg mL^−1^ (black, Azo-FF; blue, Azo-FI; orange, Azo-FV; green, Azo-FL). The data are plotted as the average of three measurements, with the error bars representing the standard deviation.

Here, we describe a series of functionalized dipeptides with the N-terminus modified by an azobenzene moiety (Fig. 1a), while retaining a free C-terminus. At high pH, where the *C*-terminus is deprotonated, micellar aggregates are formed. For such systems, it is difficult to predict which micellar structures will be formed, but the use of hydrophobic amino acids favors persistent micelles^20,25^. Azo-FF forms a liquid crystalline phase at 10 mg mL^−1^ (Fig. S31), while Azo-FI, Azo-FV, and Azo-FL form free-flowing solutions with varying degrees of turbidity (Fig. 1b). The small angle X-ray scattering (SAXS) data for Azo-FF are best fit to a power law combined with a flexible elliptical cylinder model, showing the presence of persistent worm-like structures with a radius of 9.1 nm and an axis ratio of 2.35 (Fig. 1c and Table S1). For Azo-FI and Azo-FV, the data are best fit to a power law combined with a sphere model, showing the presence of spherical micelles with a radius of 2.4 nm in each case (Fig. 1c and Table S2 and S3, respectively). The data for Azo-FL are best fit to a power law combined with a cylinder model, showing the presence of cylindrical micelles with a radius of 3.5 nm (Fig. 1c and Table S4). Hence, as for other functionalized dipeptides at high pH^21,25^, different micellar aggregates exist depending on the dipeptide sequence used. The viscosity of each solution correlates with the SAXS data, with the cylindrical structures resulting in higher viscosity solutions than the spherical micelles (Fig. 1d).

**Fig. 2:**
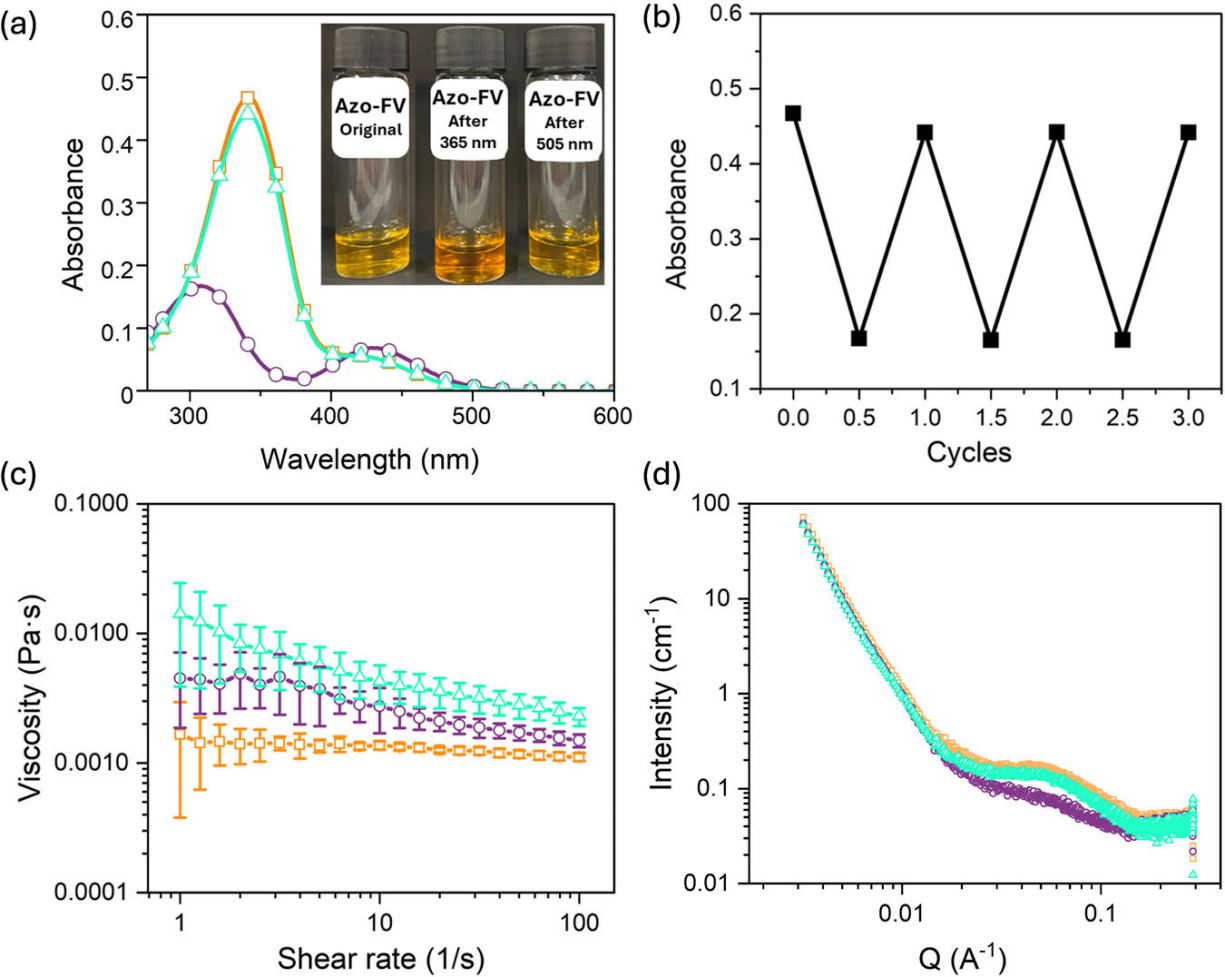
Optical and morphological data for Azo-FV. All data were collected at pH 10.5 at a concentration of 10 mg mL^−1^. **a** UV-vis spectra for the initial *trans*-isomer (orange squares), after isomerization to the *cis*-isomer with 365 nm irradiation (purple circles), and after reversal back to the *trans*-isomer with 505 nm irradiation (pale blue triangles). The inset shows a photograph of the initial *trans*-isomer (left), the *cis*-isomer (middle) and the *trans*-isomer after reversal (right) and the data in (**a**) were recorded from these solutions; **b** Change in absorbance at 340 nm over multiple *trans*-to-*cis*-to-*trans* isomerizations; **c** Change in viscosity form the initial *trans*-isomer (orange), after isomerization to the *cis*-isomer with 365 nm irradiation (purple), and after reversal back to the *trans*-isomer after 505 nm irradiation (pale blue); **d** SAXS data for the initial *trans*-isomer (orange), after isomerization to the *cis*-isomer with 365 nm irradiation (purple), and after reversal back to the *trans*-isomer after 505 nm irradiation (pale blue). All isomerizations were carried out by placing two LEDs 5 cm away from the sample and irradiating for 10 minutes.

All examples show the expected *trans*-to-*cis* isomerization when irradiated with 365 nm light (Fig. 2a and Fig. S32). Irradiation with 505 nm light reverses the azobenzene back to the *trans* state (Fig. 2a and Fig. S32). As for most examples, full reversal to the *trans* isomer does not occur (a 95% recovery is observed), but this cycle can be carried out many times (Fig. 2b and Fig. S33). The isomerization process also induces a notable change in viscosity (Fig.2c and Fig. S34), which is attributed to a reorganization of the self-assembled structures (Fig. 2d and Fig. S34); for Azo-FV, like the *trans* isomer, the data for the *cis* isomer fit best to a sphere combined with a power law model, with the spheres having a higher radius of 3.6 nm. On reversal back to the *trans* isomer, the SAXS data (all four samples was prepared in water at pH 10.5 at concentration of 10 mg mL^−1^) show that the original self-assembled structures are reformed, with the spheres having a similar radius of 2.6 nm (Fig. 2d, Fig. S35 and Table S1–S4). Slight differences in the viscosity after reisomerization can be attributed to the history of the sample, driven by stirring and relaxation times. Considering these values are all low, we would not read too much into this. All the above data were collected using LEDs as opposed to using solar energy. Due to the spectral overlap for the two isomerization processes, use of a solar simulator does enable the *trans*-to-*cis* and also *cis*-to-*trans* isomerization, but not effectively (Fig. S36). The conversion rate reaches a plateau at approximately 50% of the *trans*-isomer. However, using a 390 nm short-pass filter lens for the *trans*-to-*cis* step and a 496 nm long-pass filter lens for the *cis*-to-*trans* isomerization shows significant isomerization (Fig. S37), showing the potential here for direct use of sunlight.

**Fig. 3:**
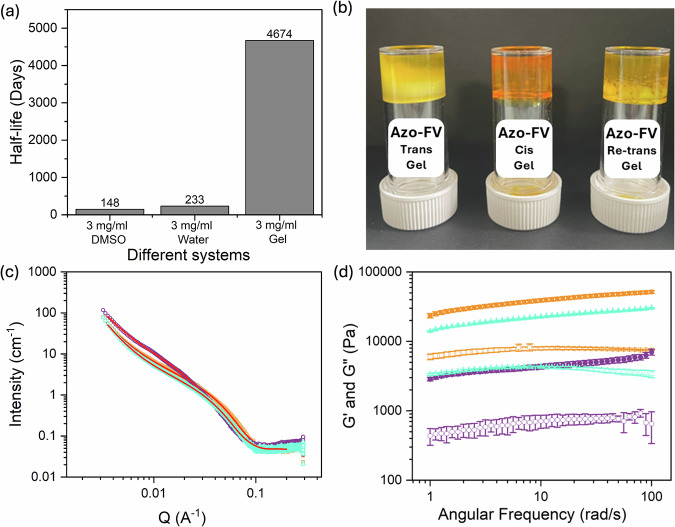
Half-lives and behavior of Azo-FV gels at low concentration. **a** Calculated half-lives for the Azo-FV *cis*-to-*trans* isomerization at 20 °C (data were determined from solutions at 3 mg mL^−1^); (**b**) photographs of Azo-FV gels formed from the *trans*-isomer (left), the *cis*-isomer (middle) and after reversal back to the *trans*-isomer (right). All data were collected at pH 10.5 at a concentration of 5 mg mL^−1^; (**c**) SAXS data for Azo-FV gels formed from the *trans*-isomer (orange) and *cis*-isomer (purple) and after reversal back to the trans-isomer (light blue) with fits to the data shown as red lines. All data were collected at pH 10.5 at a concentration of 5 mg mL^−1^; (**d**) rheology of Azo-FV gels formed from the *trans*-isomer (orange), the *cis*-isomer (purple) and after reversal back to the *trans*-isomer (light blue). All data were collected at pH 10.5 at a concentration of 5 mg/mL. All isomerizations were carried out by placing two LEDs 5 cm away from the sample and irradiating for 10 min.

From the perspective of potential candidates for energy storage, we investigated the energy released from the bulk solids for each of the functionalized dipeptides after irradiation to the *cis*-isomers. There was a significant energy release on heating as expected as *cis*-to-*trans* isomerization occurred. By maintaining a constant heating rate (at 10 °C min^−1^) during differential scanning calorimetry (DSC) for energy storage capacity determination, it was found that the energy densities of the four molecules were comparable to those of other azobenzene-based systems^24^. Among them, Azo-FV exhibited the highest energy release (151 J g^−1^, Figs. S38 and S39), and was therefore selected for further investigation. All the molecules are stable to at least 250 °C under an inert atmosphere (Fig. S40). As noted above, we hypothesized that preparing a micellar phase formed by functionalized azobenzenes would reduce the rate of the *cis*-to-*trans* back conversion. Specifically, the half-life of the *cis*-to-*trans* isomerization at 20 °C was 148 days as a solution at a concentration of 3 mg mL^−1^ in DMSO (this concentration was chosen due to the absorbance; Fig. 3b and Fig. S41). As a micellar dispersion in water at high pH at the same concentration, the half-life was 233 days (Fig. 3a and Fig. S42 for calculations). We ascribe this increase in half-life to the formation of the micelles; for the isomerization to occur, we presume that there is a need for multiple isomerizations to occur concurrently to ensure packing within a micellar structure is effective. On top of this, association between molecules within the self-assembled aggregates driven by non-covalent interactions such as hydrogen bonding and stacking will also likely enhance the activation barrier for *cis*-to-*trans* isomerization as suggested elsewhere^22^, leading to an increase in the half-life.

To further increase the half-life, we formed a gel by the addition of a calcium salt to the micellar dispersion at high pH at a concentration of 5 mg mL^−1^. As for related systems^20^, the divalent salt crosslinks the micelles present at high pH leading to a self-supporting gel (Fig. 3b). Both the *trans*-isomer and the *cis*-isomer form gels on addition of a calcium salt. Interestingly, if the *trans*-gel is irradiated, the gel falls apart but adding a calcium salt to a preformed solution of the *cis*-isomer results in a gel that is then stable when irradiated to the *trans*-isomer. Using SAXS to understand the underpinning network, for the gel formed from the *trans* isomer of Azo-FV, the data fit best to a power law combined with an elliptical cylinder model with a radius of 2.6 nm and an axis ratio of 1.9. Hence, the addition of the calcium salt leads to a morphological change from spheres to elliptical cylinders; the gel is formed by the entangled network of these structures. The data for the gel formed from the *cis* isomer also fit best to a power law combined with an elliptical cylinder model, with the cylinders having a higher radius of 3.2 nm and an axis ratio of 3.2. On reversal back to the *trans* isomer, the SAXS data show that the original self-assembled structures are reformed, with the cylinders having a similar radius of 2.8 nm (Fig. 3c and Table S5). All gels have a storage modulus (G′) that is significantly higher than the loss modulus (G″) (Fig. 3d). The *cis*-gel is mechanically weaker than the *trans*-gel, and the original *trans*-gel is stiffer than the *trans*-gel reformed after irradiation with a 505 nm LED. Again, this shows the process dependency of such systems and may also be related to the fact that the steady state after irradiation is not quite the same as that of the original system (Fig. 2a). An important result of the gelation is that the half-life for the *cis*-to-*trans* isomerization is increased significantly as compared to the starting micellar solution to 4674 days (Fig. 3a and Fig S43). This substantial increase in half-life can be attributed to additional interactions from non-covalent crosslinking, as well as increased molecular rigidity within the gel network, which collectively raise the activation barrier for *cis*-to-*trans* isomerization. Indeed, the activation energies (*Eₐ*) for the cis-to-trans isomerization of Azo-FV were calculated to be 85.4 kJ mol⁻¹ in DMSO, 100.3 kJ mol⁻¹ in the micellar state, and 166.1 kJ mol⁻¹ in the gel state (see Supporting Information, page S48). It is worth noting that gels formed through the self-assembly of small molecules are often metastable. However, they can remain stable for extended periods. In fact, this has been experimentally confirmed, as we have gel samples in our laboratory that have maintained their structure and properties for several years.

**Fig. 4:**
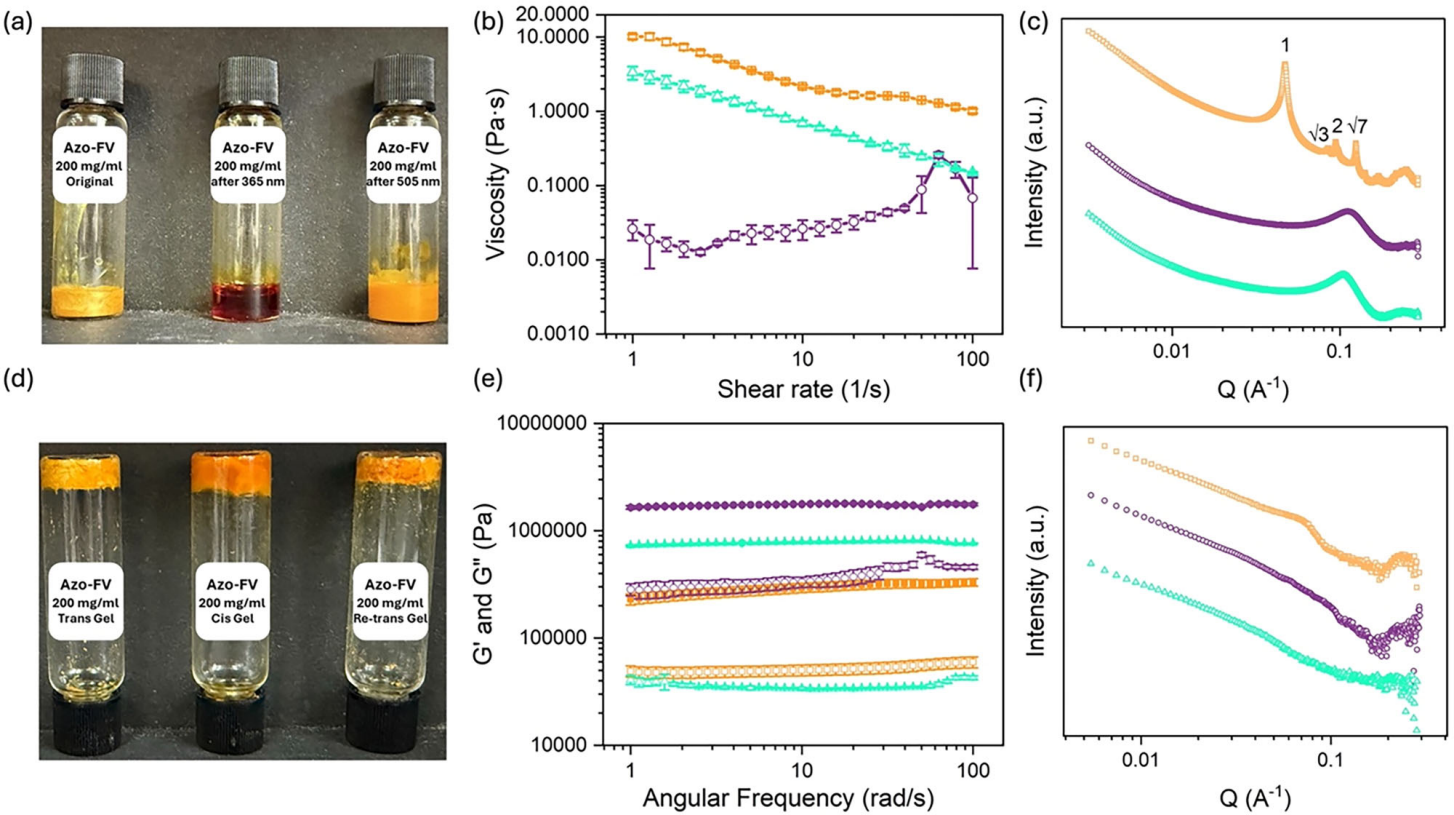
Behavior of Azo-FV systems at high concentration. **a** Photographs of solutions formed from the initial Azo-FV *trans*-isomer (left), the *cis*-isomer (middle) and the *trans*-isomer after reversal (right), all at 200 mg mL^−1^; **b** viscosity data for solutions formed from the *trans*-isomer (orange), the *cis*-isomer (purple) and after reversal back to the *trans*-isomer (light blue); **c** SAXS data for the solutions formed from initial *trans*-isomer (orange), the *cis*-isomer (purple) and the *trans*-isomer after reversal (light blue), all at 200 mg mL^−1^; **d** photographs of gels formed from initial *trans*-isomer (left), the *cis*-isomer (middle) and the *trans*-isomer after reversal (right), all at 200 mg mL^−1^, on addition of a calcium salt; **e** rheology of gels formed at 200 mg mL^−1^ from the trans-isomer (orange), the *cis*-isomer (purple) and after reversal back to the *trans*-isomer (light blue); **f** SAXS data for the gels formed from initial *trans*-isomer (orange), the *cis*-isomer (purple) and the *trans*-isomer after reversal (light blue), all at 200 mg mL^−1^. All isomerizations were carried out by placing two LEDs 5 cm away from the sample and irradiating for 10 min.

From a practical application standpoint, whilst the use of water as a medium is particularly appealing, especially for future industrial-scale MOST systems due to its safety compared to organic solvents and its high heat capacity for heat transfer purposes, there have been very few demonstrations of azobenzene derivatives in aqueous solutions for real-world applications. With our materials, micellar aggregates can be formed at a concentration of 200 mg mL^−1^ (Fig. 4a, left), resulting in an extremely viscous solution (Fig. 4b). Irradiation with 365 nm light results in a clear change in color and turbidity (Fig. 4a, middle) with a significantly lower viscosity (Fig. 4b); irradiation with 505 nm light results in a solution with similar color and turbidity to the original solution (Fig. 4a, right) and a higher viscosity. This higher viscosity likely reflects the process of forming the *trans*-isomer from the *cis*-isomer as compared to direct dissolution. SAXS data for these solutions shows that the initially formed solution contains sharp Bragg diffraction peaks (Fig. 4c), indicating the formation of a lyotropic liquid crystal (LLC) phase; the peaks have a *q* ratio of 1:√3:2:√7, characteristic of a reverse hexagonal phase (Fig. 4c)^26^. In agreement with this assignment, we observe a birefringent, densely-packed fan pattern by cross-polarized microscope (CPM, Fig. S44). On irradiation, this order is lost, and the data show the presence of a Bragg peak at 56.8 Å (Fig. 4c). After re-isomerization to the *trans*-isomer using irradiation from a 505 nm LED, the original scattering data are not recovered but shows the presence of a Bragg peak at a similar position to that present in the *cis*-isomer (Fig. 4c). These data therefore agree with the viscosity data showing that there is a process dependency in the solutions formed. We note that fitting these SAXS data as we were able to do for the low concentration samples is highly challenging due to the high concentration of structures present that renders the models invalid.

Gelation can again be achieved by the addition of a calcium salt (Fig. 4d), with extremely stiff gels being formed at this concentration (Fig. 4e). In all cases, G′ is again significantly higher than the loss modulus (G″) and, importantly for real applications, at this concentration the gel state persists when the *trans*-gel is isomerized to the *cis*-gel with irradiation from a 365 nm LED. The SAXS data are complicated and (due to the high concentration) difficult to fit, but again it is clear that the data for the initial *trans*-gel and the resulting *cis*-gel are different to one another (Fig. 4f). Re-isomerization to the *trans*-gel does not fully restore the original SAXS profile; instead, the data more closely resemble those of the *cis*-gel (Fig. 4f), again indicating a strong dependence on the isomerization history and process pathway. We highlight here that many of our systems show such a process dependence where the structures formed depend on the route used to form them.

**Fig. 5:**
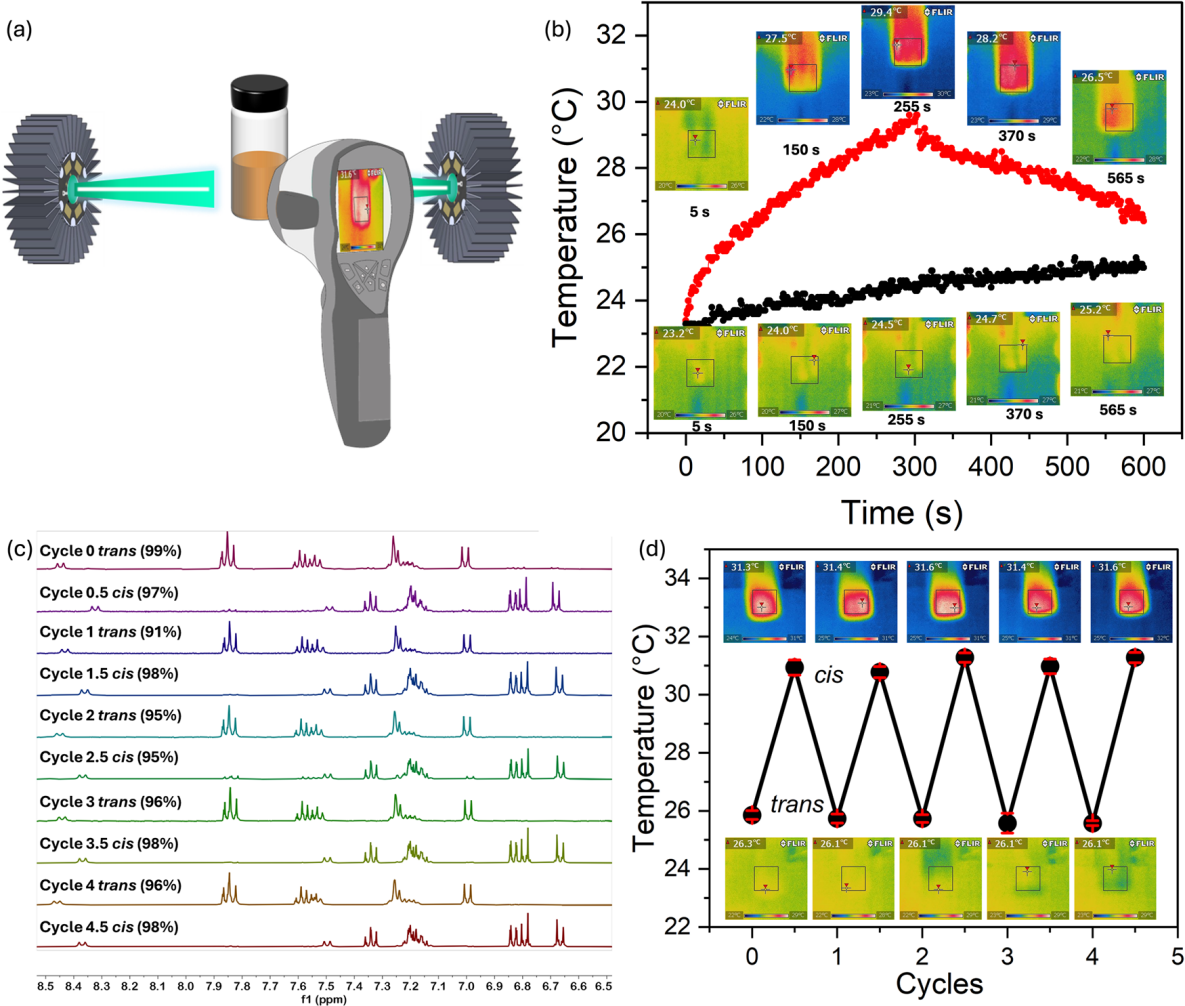
Demonstration of the MIST system. **a** Experimental setup used to monitor heat release. 400 μL of a gel formed from the Azo-FV *cis*-isomer was placed in a 3.5 mL glass vial and irradiated with two 505 nm LEDs. A thermal camera was used to monitor the temperature; **b** Heat release from *cis*-gel of Azo-FV at a concentration of 200 mg mL^−1^ (red) as compared to water (black). The gel was kept in the dark for 1 week before the release experiment was carried out, with the data being very similar to that from a gel where heat release was measured immediately after isomerization. Inset are photographs from the thermal camera showing the raw data at specific time points (expanded versions are available in the Supporting Information to clearly see the scale bars). **c** Partial ^1^H NMR spectra for repeat cycles of irradiation on the same sample showing the repeated transition from *trans* to *cis*; **d** Heat release from the *cis*-gel and *trans*-gel over multiple cycles from the same gel. Note that the absolute temperature released in (**b**) and (**d**) are different due to different ambient conditions in our laboratory.

To further demonstrate that the system’s macroscopic heat release can be effectively harnessed for energy storage applications, a highly concentrated Azo-FV gel (200 mg mL^−1^) was employed in an on-demand heat release experiment. The *cis*-gel was found to be stable over extended periods (note that half-life calculations are not possible due to the high concentrations and resulting absorbance but visually the color of the sample is that of the *cis*-isomer and UV-vis spectroscopy of a small sample removed from the bulk and diluted shows the *cis*-isomer even after 3 months, Fig. S45). On irradiation of the *cis*-gel in a glass vial with two 505 nm LEDs, isomerization to the *trans*-gel occurs (Fig. 5a). Using a thermal camera, we find that the energy released leads to a significant increase in the temperature (up to 5.7 °C, with a theoretical value of 7.2 °C, calculation details in Supporting Information, page S51) of the vial (Fig. 5b and Fig. S46) as compared to a control with just water. Similarly, using the *trans*-solution or *trans*-gel results in a low temperature increase (Figs. S53 and S54). Further, as expected, the energy stored is concentration dependent, with a 2.9 °C increase in temperature when a lower concentration of 100 mg mL^−1^ of Azo-FV was used (Fig. S55 and S56).

Importantly, the system at 200 mg mL^−1^ can be cycled multiple times with no loss of efficiency. The degree of isomerization is essentially unchanged over 5 cycles (Fig. 5c), with very similar heat released (Fig. 5d) in each of the cycles. Note, the absolute heat released here is slightly lower than in the data shown in Fig. 5b); this can be ascribed to a difference in the room temperature when the experiments were carried out. The micellar structures underpinning these gels are similar at each point, although there are slight changes in the scattering (Fig S60). However, the isomerization from *trans*-to-*cis* is unaffected by this (Fig. 5c) allowing the effective use for energy storage and release.

## Conclusions

Here, we have introduced a MIST molecular design strategy for azobenzene-based energy storage systems by coupling the photoresponsive functionality with dipeptide-driven self-assembly. By precisely controlling molecular architecture and phase state, we achieve tunable energy storage lifetimes, culminating in exceptionally long storage durations, and reversible, light-triggered energy release across organic, aqueous, and gel environments. In future studies, density functional theory (DFT) calculations could be employed to elucidate the mechanisms by which self-assembled structures significantly extend the half-life of the *cis*-isomer, as well as to provide insight into the structural transition from the pre-gel to the gel state. It is generally accepted that the half-life of the photoisomer increases with concentration; however, a key limitation in our current system is the solubility of the azobenzene dipeptide. At higher concentrations, the material becomes increasingly viscous, thereby impeding light penetration and affecting the photoconversion process. To address this, future molecular design of MIST compounds will focus on improving solubility, particularly by introducing structural modifications to the azobenzene core to reduce π–π stacking interactions or by incorporating steric hindrance to further stabilize the *cis*-isomer and hinder its thermal back-conversion. Another promising strategy involves exploring solvents with lower polarity and specific heat capacity, such as ethylene glycol, which may improve solubility and enhance system performance. With these optimizations, we anticipate that the heat release performance could be elevated to the average range of current MOST systems. The formation of highly stable, water-compatible materials that retain functionality at high concentrations and enable macroscopic heat release marks a significant advancement toward scalable, deployable energy storage technologies. These findings expand the design landscape for molecular solar thermal materials and establish a versatile platform for developing next-generation energy storage systems that unify responsiveness, durability, and processability within a single molecular framework.

## Methods

### General experimental

All chemicals were used as received. Deionized water (Avidity Pico10T2) was used in all experiments. ^1^H and ^13^C NMR spectra were recorded on a Bruker Avance^III^ HD or Bruker Avance^III^ NEO 400 MHz spectrometer. Chemical shifts (*δ*) are reported in parts per million (ppm) to the residual solvent peak (CDCl_3_: 7.26 ppm for ^1^H and 77.0 ppm for ^13^C; DMSO–*d*_*6*_: 2.50 ppm for ^1^H and 39.52 ppm for ^13^C). Absorption spectra were measured on an Agilent Cary 60 UV–vis spectrophotometer. Thermogravimetric analysis (TGA) was performed on the TA Instruments TGA 5500 under N_2_. Microscope images were taken on a Nikon Eclipse LV100ND with a 5× magnification. Images were collected under cross polarized light at 90° and non-polarized light. Scale bars were added to images using the software, ImageJ. All heat release pictures were carried out using a Teledyne FLIR thermal camera i7. The commercial 365 nm LED (829–0841) was positioned to give approximately 80 mW cm^−2^ and commercial 505 nm LED (358–3121) was positioned to give approximately 20 mW cm^−2^ for the experiments.

### Materials

Full synthetic details and characterization are described in the Supporting Information.

### Solution preparation

The general preparative procedure is as follows: azobenzene dipeptide solutions were prepared by weighing azobenzene dipeptide into a vial. The required volume of water was added to reach a concentration of 5, 10, or 200 mg mL^−1^. Following this, 2 equivalents of sodium hydroxide aqueous solution (2 M) was added. This mixture was stirred continuously for approximately 17 hours at ambient temperature (around 20 °C) using a 25 × 8 mm stirrer bar within a 50 mL centrifuge tube at a speed of 1000 RPM. The pH of the resulting solution was adjusted to 10.5 ± 0.1 using aqueous sodium hydroxide (1 M). The *cis*-isomer solutions were prepared by irradiating the fresh solutions with a 365 nm LED for 10 minutes, and the *re-trans*-isomer solutions were prepared by irradiating the *cis*-isomer solutions with a 505 nm LED for 10 minutes.

### Gel preparation

Gels were prepared from the previously described pre–gelation solutions. Subsequently, 8 μL of an aqueous calcium chloride solution (5.0 M) was added to 2.0 mL of the pre-gelation solution to trigger gelation by depositing the drop on top of the solution. The samples were left to stand for 24 h incubation period at ambient temperature (around 20 °C). The *cis*-isomer gel and *re–trans*-isomer gel can be obtained by adding the calcium chloride trigger to *cis*-isomer solutions and *re–trans*-isomer solutions respectively.

### Rheology

All rheological measurements were carried out using an Anton Paar Physica MCR101 rheometer. Viscosity measurements were performed using a 50 mm cone geometry (CP50) with gap distance between the geometry and the plate set to 0.10 mm and temperature set to 25 °C. Samples were poured onto the rheometer flat plate for measurement and fresh sample used for each run. For the gels, strain and frequency–sweep measurements were conducted in a vane–cup measurement system (Anton Paar ST10–4V–8.8/97.5) and samples were prepared in a plastic Sterlin cup (7 mL volume) and incubated at ambient temperature (around 20 °C) for 24 h before each measurement. Rheology measurements were performed in triplicate, and values averaged with error bars representing the standard deviation between the repeats.

### Small–angle X–ray scattering

Small–angle X–ray scattering (SAXS) measurements were conducted at the Diamond Light Source, Didcot, on the I22 beamline. The beamline operated at an energy of 12.4 keV, with a detector distance of 9.750 m, providing a final Q range of 0.0016 to 0.177 Å⁻¹. Samples were prepared as previously described and transferred into borosilicate glass capillaries (1.55 mm internal diameter) using a 1 mL syringe fitted with a 21 G needle. For all samples, 10 × 100 ms frames exposure were collected and averaged. The raw data was processed using Dawn Science software (version 2.27)^27^ following the standard I22 pipeline^28^. During processing, background subtraction was performed on the raw 2D SAXS data, followed by full azimuthal integration to generate an *I* vs *q* plot. The resulting plots were then fitted to structural models using SasView software (version 5.0.4)^29^.

### Differential scanning calorimetry and thermogravimetric analysis

Differential scanning calorimetry (DSC) measurements were conducted on a TA Instruments DSC 25. Azobenzene dipeptides, (about 100 mg) were dissolved in deuterated acetone (about 7 mL). After irradiation for 10 min using the LED (emitting at 365 nm) and evaporation of the solvent, 94% conversion to *cis*-isomer was confirmed by ^1^H NMR spectroscopy. A pre-weighed amount (~3 mg) of the *cis*-isomer azobenzene dipeptides were sealed and inserted in the DSC at 20 °C. The DSC method involved running two consecutive cycles, each consisting of heating at 10 °C min^−1^ from 20 °C to 150 °C with 50 mL min^−1^ nitrogen and in between cooling (at 10 °C min^−1^ from 150 °C to 20 °C with 50 mL min^−1^ nitrogen), conversion to the *trans*-isomer was confirmed by ^1^H NMR spectroscopy after the DSC measurements. The first heating cycle showed the characteristic exothermic feature associated with back isomerization for Azo-FF and Azo-FV, and the second heating cycle did not show any evidence of any thermal transitions, confirming that the full back isomerization occurred in the first cycle. However, Azo-FI and Azo-FL still have exothermic peaks in the second heating cycle, a part of *cis*-isomers are not converted to *trans*-isomers was confirmed by ^1^H NMR spectroscopy after DSC measurements. Integration of the exothermic peak and normalization respect to the amount of *cis*-iosmer azobenzene dipeptides present in the neat material gave an enthalpy difference ($\Delta {H}_{{storage}}$) between *cis*- and *trans*-iosmer azobenzene dipeptides.

### Heat release experiments

Heat release experiments were measured using a Teledyne FLIR thermal camera i7. The sample was placed between two LEDs (LedEngin Inc 505 nm, Ultraviolet LED, 350 mA), 5 cm away for 10 minutes, with data being collected every second.

For the cycling experiments, the 200 mg mL^−1^ Azo-FV *trans*-gel was placed between two LEDs (LedEngin Inc 365 nm, Ultraviolet LED, 700 mA), 5 cm away for 10 min. A small amount (sampling was performed using a 1.1 × 38 mm needle from TERUMO) was removed and diluted in 3 mL DMSO and DMSO–*d*_*6*_ to collect UV–vis spectra and ^1^H NMR spectra for comparison. Then the sample was placed between two LEDs (LedEngin Inc 505 nm, Ultraviolet LED, 350 mA), 5 cm away for 10 min and a small sample was again collected for UV–vis spectra and ^1^H NMR spectra as above. This was repeated three times in total. Throughout, a Teledyne FLIR thermal camera i7 was used to monitor temperature changes during the entire process.

### Using a solar simulator

An Abet Technologies’ model 11002 SunLite™ Solar Simulator was used to irradiate at a distance of 5 cm from solutions of 3 mg mL^−1^ Azo-FV in DMSO, with UV-vis spectra collected every 2 min. A control experiment was also conducted using a solution of the *cis*-isomer solution pre-formed using direct irradiation with a 365 nm LED. Where noted, we used a THOR LABS 390 nm short-pass filter lens and a 496 nm long-pass filter lens above the sample to block specific wavelengths of light.

## Supplementary information

Supplementary Information

Description of Additional Supplementary Files

Supplementary data 1

## Data Availability

All data underlying the graphs have been uploaded as Supplementary Data. All primary images and other data are available from the authors on request.
