# Supplementary material for: Functionalized azobenzenes for micellar solar thermal energy storage as a next-generation MOST system
Source: Commun Chem. 2025 Nov 24;8:369. doi: 10.1038/s42004-025-01750-5 (PMC12644887; doi:10.1038/s42004-025-01750-5)
Supplement: Supplementary file 1 — Supplementary Information [file 42004_2025_1750_MOESM1_ESM.pdf]

Supplementary Information

Functionalized Azobenzenes for Micellar Solar Thermal Energy Storage as a Next-Generation MOST System

Rui Huang,[1] Alex S. Loch,[1] Alice Pincham,[2] Andrew J. Smith,[3] Annela Seddon,[2] Zhihang Wang,[4,5*] and Dave J. Adams[1*]

1. School of Chemistry, University of Glasgow, Glasgow, G12 8QQ, UK
2. School of Physics, HH Wills Physics Laboratory, University of Bristol, Bristol BS8 1TL, U.K.
3. Diamond Light Source Ltd, Diamond House, Harwell Science and Innovation Campus, Didcot, UK
4. School of Engineering, College of Science and Engineering, University of Derby, Markeaton Street, Derby DE22 3AW, United Kingdom.
5. Department of Materials Science and Metallurgy, University of Cambridge, 27 Charles Babbage Road, Cambridge, CB3 0FS, United Kingdom.

Supplementary Methods

Synthesis

Synthesis of 4-(phenyldiazenyl)phenol (Compound **1**):

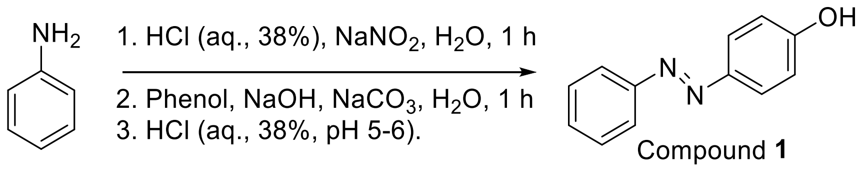

A solution of aniline (1.86 g, 20.0 mmol) in water (50 mL) and aqueous hydrogen chloride (8.5 mL, conc. 38%, 110.0 mmol) was stirred and cooled in an ice bath to which a separate solution of sodium nitrite (2.00 g, 29.0 mmol) in water (50 mL) was added dropwise. The reaction mixture was then added dropwise to a solution of sodium hydroxide (1.04 g, 26.0 mmol), sodium carbonate (9.60 g, 90.0 mmol), and phenol (2.70 g, 28.7 mmol) in water (50 mL) that was cooled in ice bath. The reaction mixture was stirred for 1 hour before the temperature was allowed to raise to room temperature over 30 minutes. The solution was acidified with concentrated aqueous hydrogen chloride (38%) until the pH was between 5-6 at which a brown solid precipitated from the solution. The solid was collected at the filter, washed with water (200 mL), and dried in a vacuum oven at 50°C to give the product as a brown solid. Yield: 8.32 g, 83%. ^{1}H NMR (400 MHz, $CDCl_3$) δ: 7.93–7.87 (4H, m, $\underline{H}_{Ar}$), 7.52–7.42 (3H, m, $\underline{H}_{Ar}$), 6.94 (2H, d, J = 8.3 Hz, $\underline{H}_{Ar}$), 5.32 (1H, bs, O$\underline{H}$). ^{13}C NMR (101 MHz, $CDCl_3$) δ: 158.4, 152.8, 147.3, 130.6, 129.2, 125.1, 122.7, 116.0. HRMS $[M+H]^+$ calculated for $[C_{12}H_{11}N_2O]^+$: 199.0866, found: 199.0872. The data are in agreement with the literature.[S1]

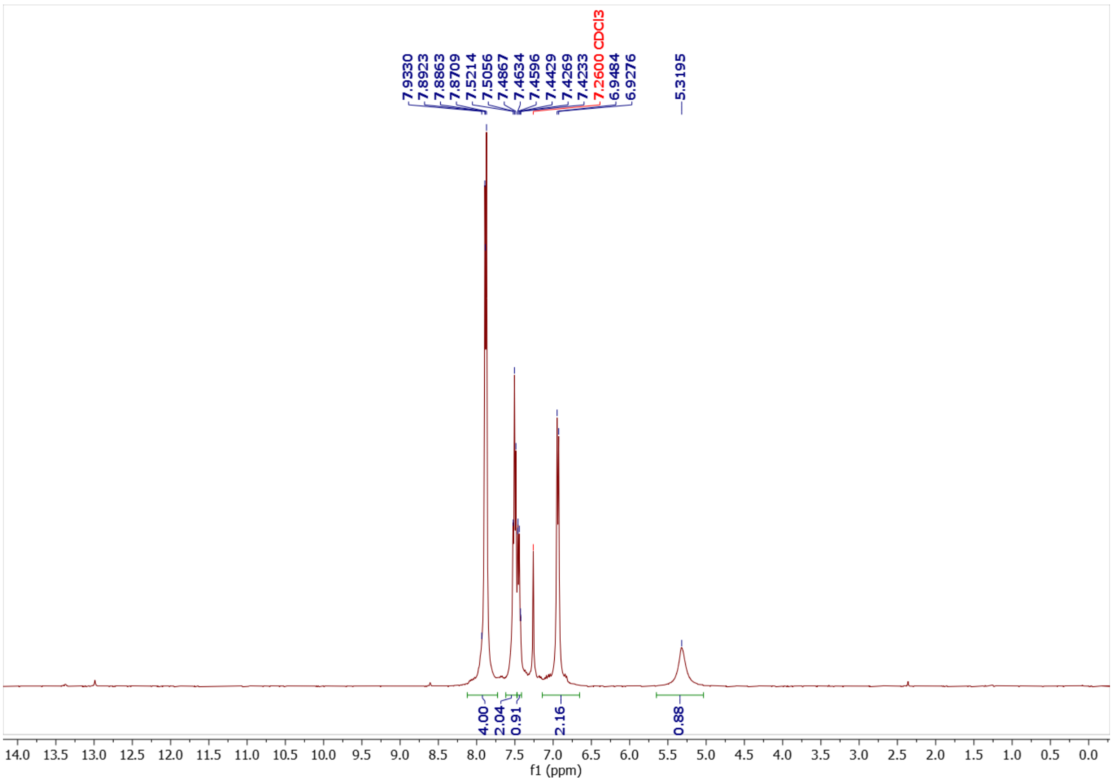

Supplementary Figure 1. ^{1}H NMR spectrum of compound **1** in $CDCl_3$.

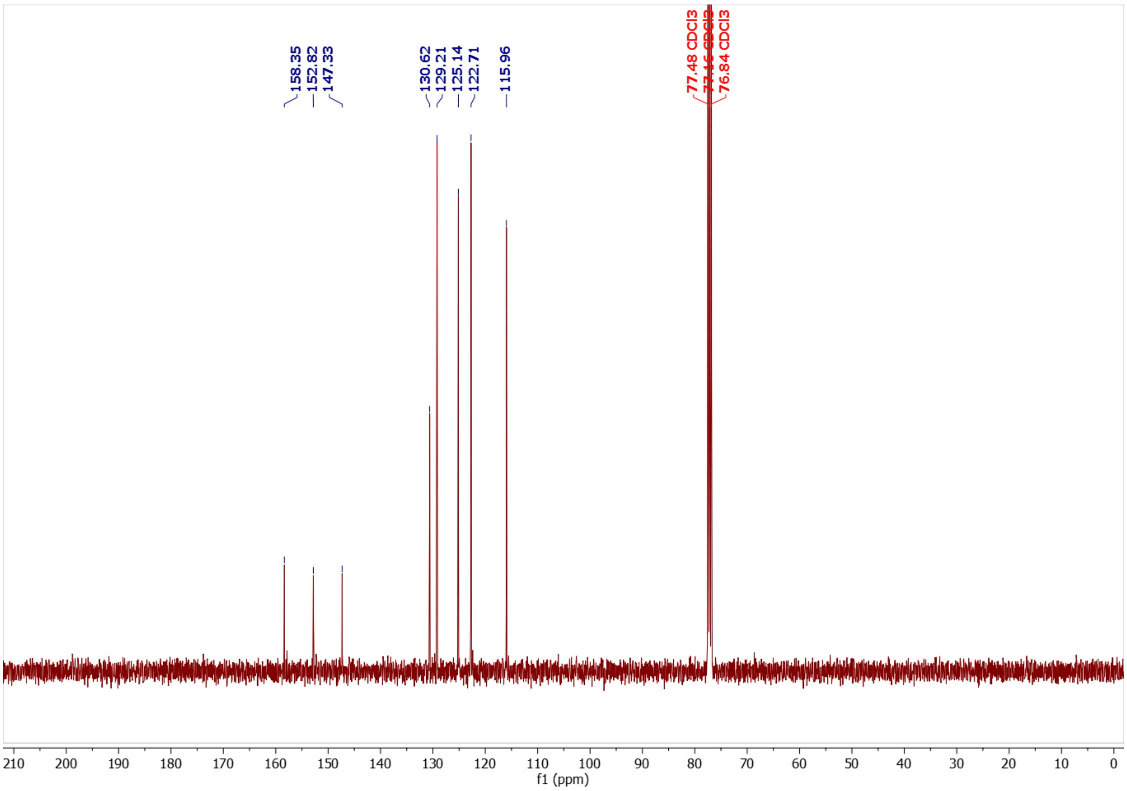

Supplementary Figure 2. ^{13}C NMR spectrum of compound **1** in $CDCl_3$.

Synthesis of *tert*-butyl 2-[4-(phenyldiazenyl)phenoxy]acetate (Compound **2**):

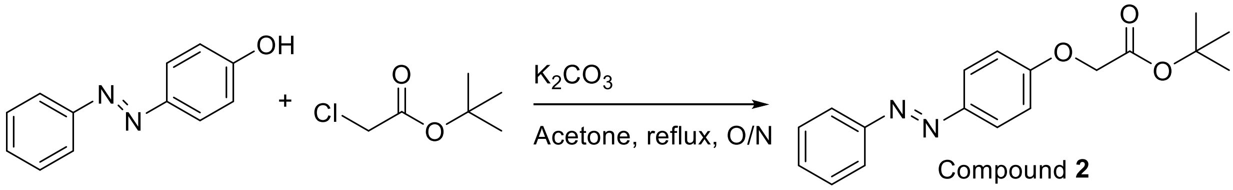

A mixture of **1** (3.96 g, 20 mmol), potassium carbonate (6.95 g, 50 mmol), and *tert*-butyl 2-chloroacetate (3.2 mL, 22 mmol) in acetone (100 mL) was refluxed for 24 h. The mixture was allowed to cool to room temperature, the white solid was filtered off, and the filtrate was evaporated to dryness. The resulting orange oil was dissolved in chloroform (75 mL) and washed with saturated sodium bicarbonate solution (200 mL × 2) and brine (200 mL). The separated organic layer was dried over anhydrous magnesium sulfate, filtered, and evaporated to dryness to yield the product as a yellow-orange solid. Yield: 5.75 g, 92%. ^{1}H NMR (400 MHz, $CDCl_3$) δ: 7.94–7.86 (4H, m, $\underline{H}_{Ar}$), 7.53–7.42 (3H, m, $\underline{H}_{Ar}$), 7.01 (2H, d, J = 9.0 Hz, $\underline{H}_{Ar}$), 4.60 (2H, s, O–C$\underline{H}_2$), 1.50 (9H, s, C$\underline{H}_3$). ^{13}C NMR (101 MHz, $CDCl_3$) δ 167.7, 160.4, 152.9, 147.6, 130.6, 129.2, 124.8, 122.8, 115.0, 82.8, 65.9, 28.2. HRMS $[M+H]^+$ calculated for $[C_{18}H_{21}N_2O_3]^+$: 312.1468, found: 312.1484.

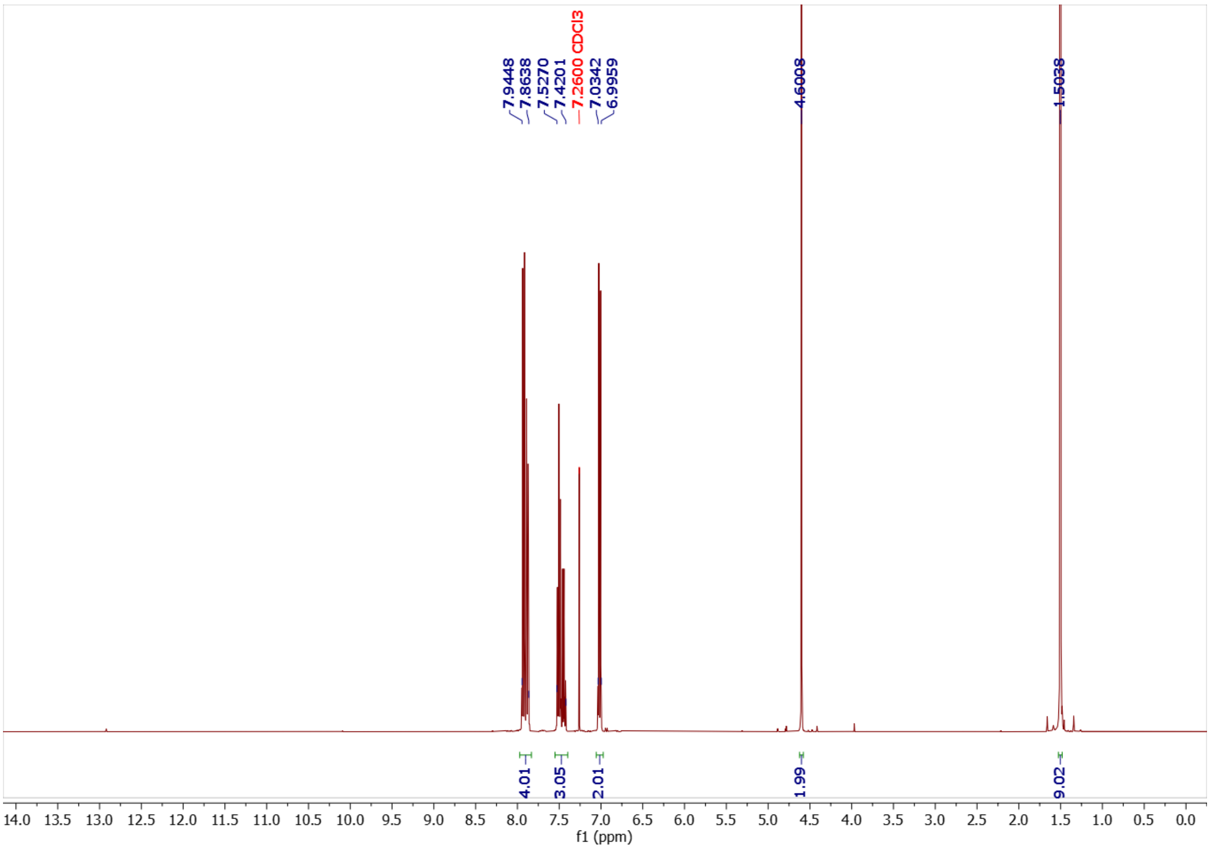

Supplementary Figure 3. ^{1}H NMR spectrum of compound **2** in $CDCl_3$.

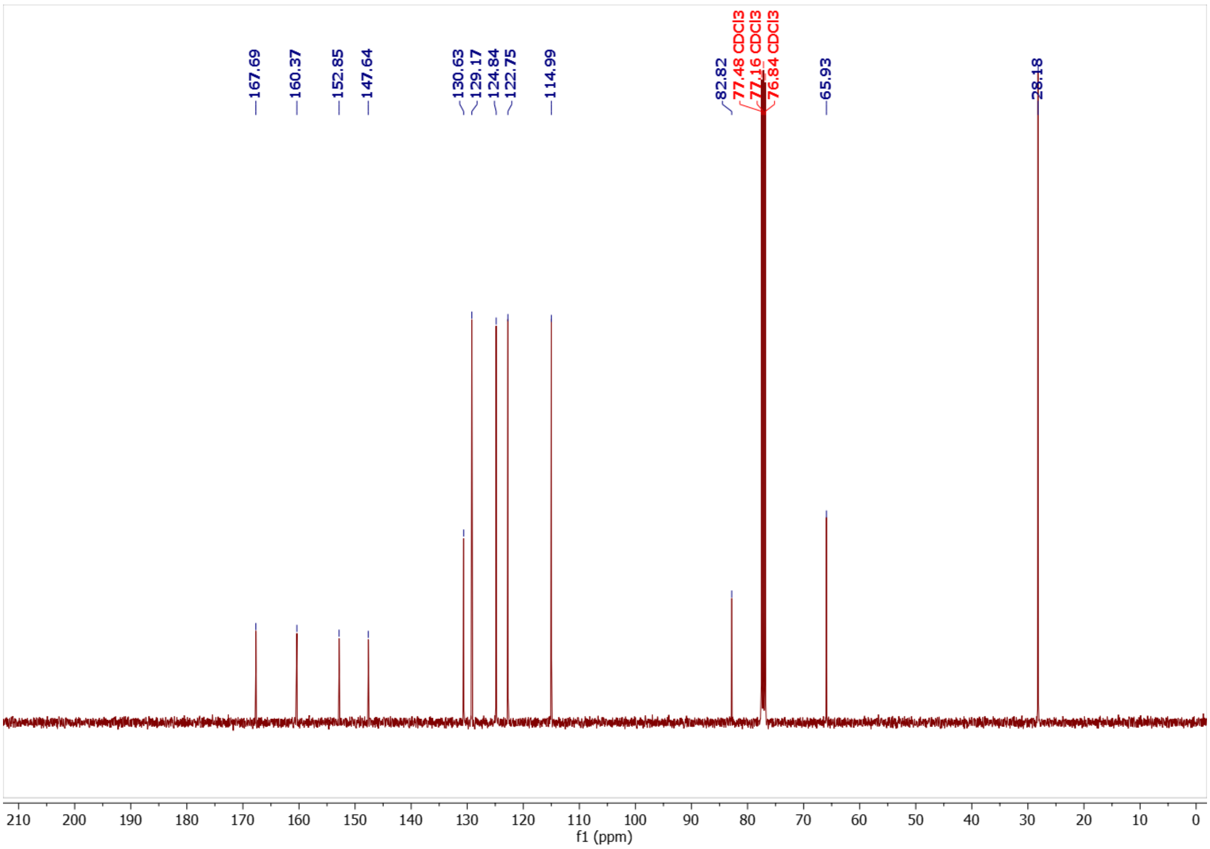

Supplementary Figure 4. ^{13}C NMR spectrum of compound **2** in $CDCl_3$.

Synthesis of 2-(4-(phenyldiazenyl)-phenoxy)-acetic acid (Compound **3**):

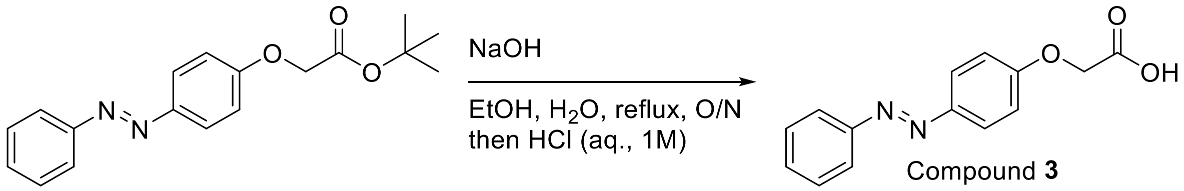

Compound **2** (4.4 g, 14.1 mmol) was dissolved in ethanol (270 mL) and a solution of sodium hydroxide (5.60 g, 141 mmol) in water (30 mL) was added. The mixture was stirred overnight at 80°C. The solution was allowed to cool and was diluted with aqueous hydrogen chloride (1 m. 300 mL) and stirred for another 1 h. The precipitated solid was collected by filtration, washed with water (500 mL), and dried to yield the product as an orange solid. Yield: 3.36 g, 93%. ^{1}H NMR (400 MHz, DMSO–d_6) δ: 13.13 (1H, s, COOH), 7.91–7.83 (4H, m, H_{Ar}), 7.60–7.51 (3H, m, H_{Ar}), 7.12 (2H, d, J = 9.5 Hz, H_{Ar}), 4.82 (2H, s, O–CH_2). ^{13}C NMR (101 MHz, DMSO–d_6) δ: 169.8, 160.5, 152.0, 146.4, 130.9, 129.4, 124.5, 122.3, 115.2, 64.7. HRMS $[M+H]^+$ calculated for $[C_{14}H_{13}N_2O_3]^+$: 257.0921, found: 257.0928.

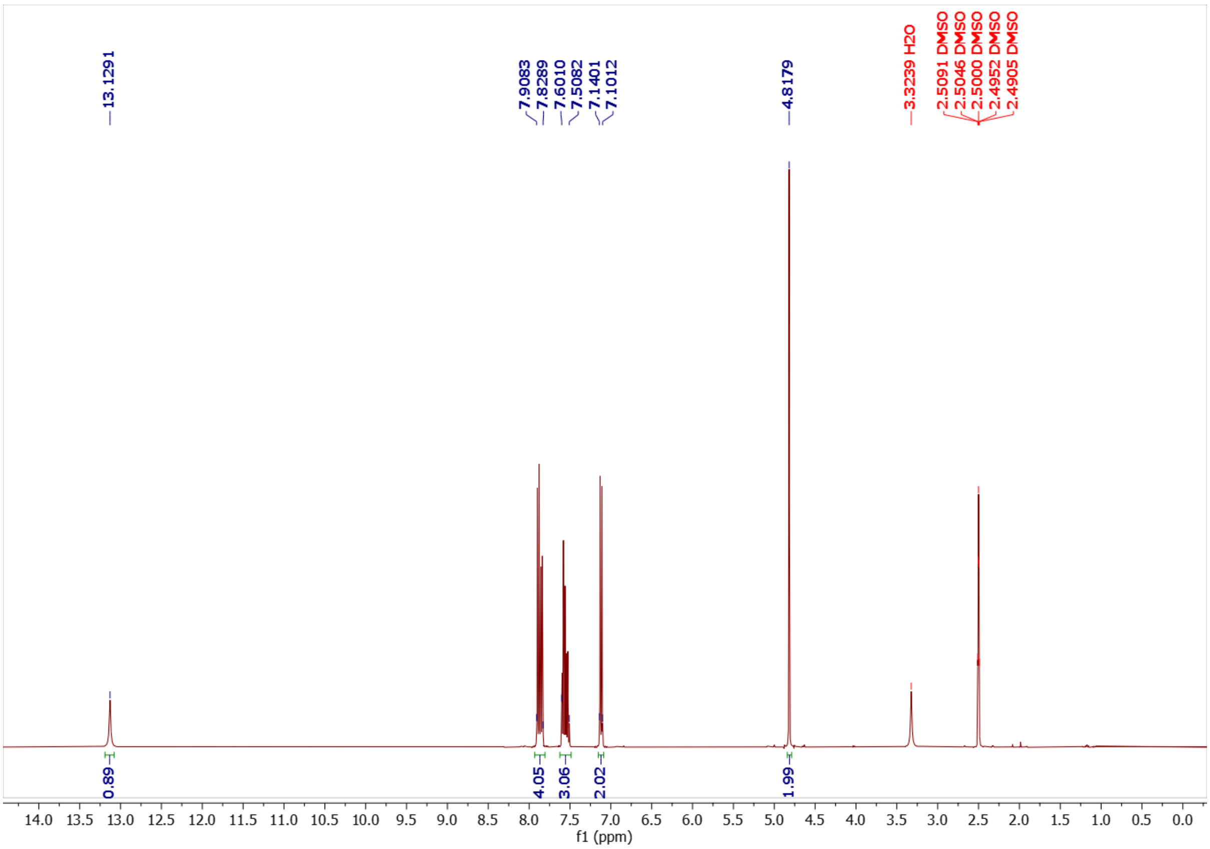

Supplementary Figure 5. ^{1}H NMR spectrum of compound **3** in DMSO–d_6. HOD from the d_6-DMSO can be seen at around 3.3 ppm.

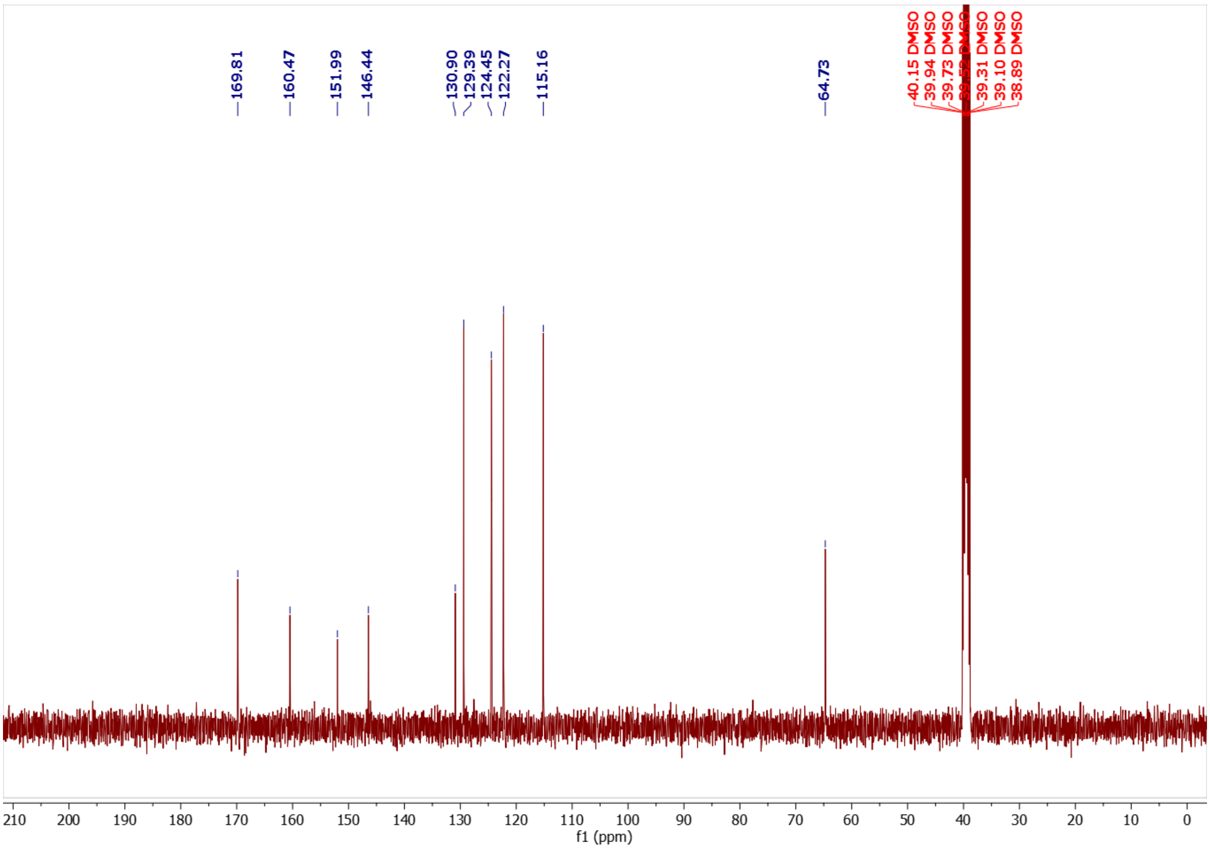

Supplementary Figure 6. ^{13}C NMR spectrum of compound **3** in DMSO–*d*$_6$.

Synthesis of methyl L-phenylalanyl-L-valinate TFA salt (Compound **4**)

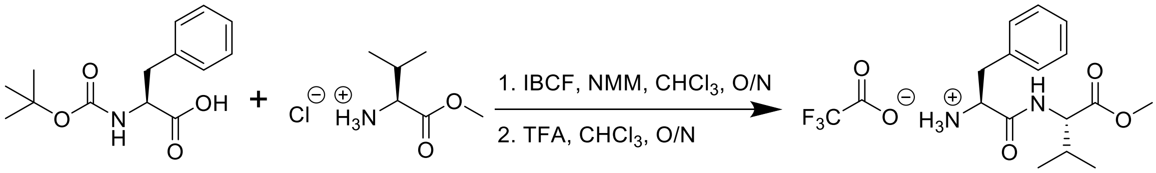

Compound **4**

To a solution of Boc-L-phenylalanine (3.0 g, 11.3 mmol) in chloroform (30 mL) were added isobutyl chloroformate (1.56 mL, 14.7 mmol) and *N*-methyl morpholine (2.60 mL, 22.6 mmol) and the mixture was stirred for 1 h. A separate solution of L-valine methyl ester hydrochloride (1.90 g, 11.3 mmol) and *N*-methyl morpholine (2.60 mL, 22.6 mmol) in chloroform (30 mL) was then added and the mixture was stirred overnight. The now clear solution was diluted with chloroform (50 mL) and washed in turn with aqueous hydrochloric acid (1 M, 200 mL), water (200 mL × 2), and brine (200 mL), and the separated organic layer was dried over anhydrous magnesium sulfate, filtered, and evaporated under reduced pressure to yield an off–white solid. The solid was dissolved in chloroform (40 mL) and trifluoroacetic acid (8.70 mL, 113.1 mmol) and the mixture was stirred overnight. The solution was concentrated under reduced pressure to a viscous oil. The resulting oil was dissolved in chloroform (30 mL) and poured into diethyl ether (400 mL) and stirred overnight. The precipitate was collected by filtration, washed on the filter with a few small portions of diethyl ether (10 mL × 3) and dried to give the product as a white solid. Yield: 3.47 g, 81 %. ^{1}H NMR (400 MHz, DMSO–*d*$_6$) δ: 8.68 (1H, d, J = 8.3 Hz, N<u>H</u>), 8.12 (2H, s, N<u>H</u>$_3^+$), 7.38–7.22 (5H, m, <u>H</u>Ar), 4.22 (1H, dd, J = 8.2, 6.2 Hz, C<u>H</u>), 4.12 (1H, dd, J = 6.8, 6.8 Hz, C<u>H</u>), 3.64 (3H, s, C<u>H</u>$_3$), 3.08 (1H, dd, J = 14.0, 5.8 Hz, C<u>H</u>$_2$), 2.94 (1H, dd, J = 14.0, 7.8 Hz,

C$\underline{H}_2$), 2.09–1.99 (1H, m, C$\underline{H}$), 0.90 (6H, dd, *J* = 6.7, 6.7 Hz, C$\underline{H}_{Leu}$). ^{13}C NMR (101 MHz, DMSO–*d*$_6$) *δ*: 169.8, 160.5, 152.0, 146.4, 130.9, 129.4, 124.5, 122.3, 115.2, 64.7. HRMS [M+H]$^+$ calculated for [$C_{15}H_{24}N_2O_3$]$^+$: 279.1703, found: 279.1703.

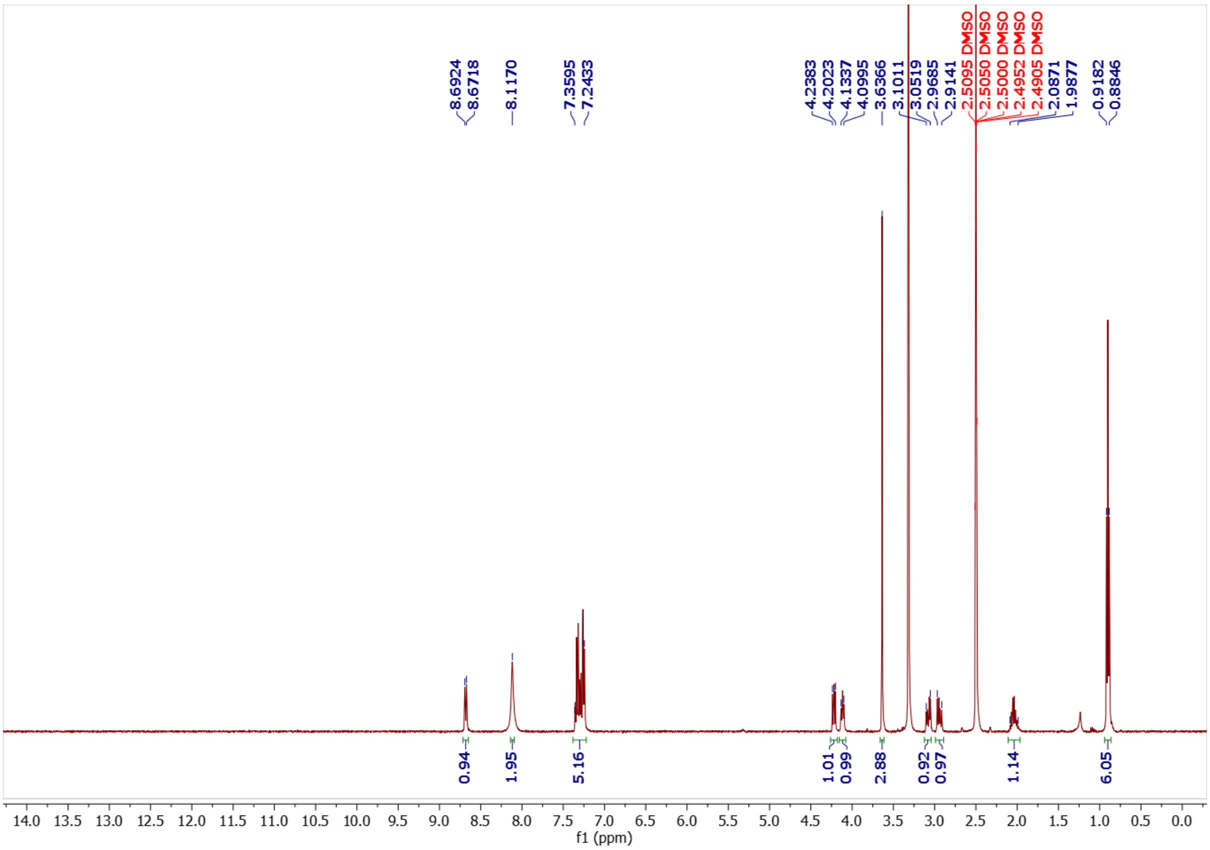

Supplementary Figure 7. ^{1}H NMR spectrum of compound **4** in DMSO–*d*$_6$.

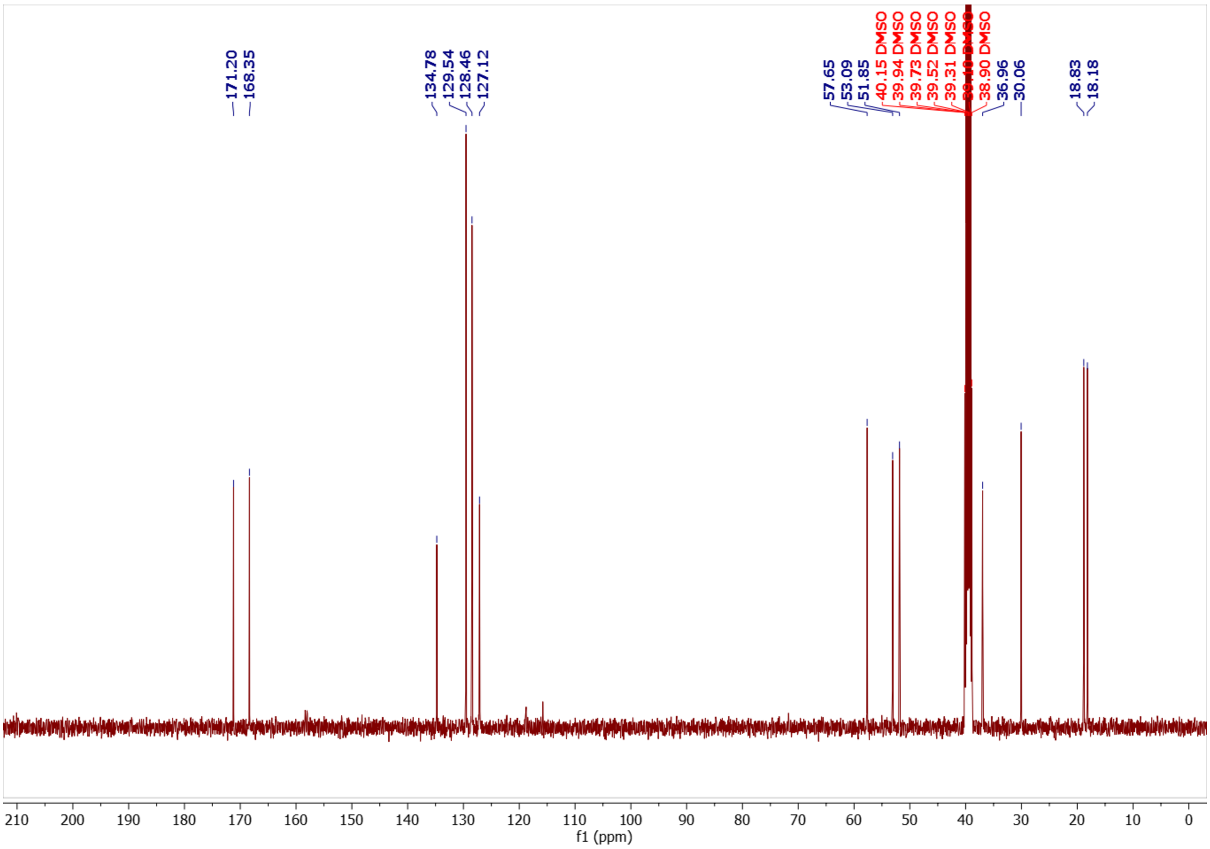

Supplementary Figure 8. ^{13}C NMR spectrum of compound **4** in DMSO–d_6.

Synthesis of methyl [2-(4-{phenyldiazenyl}phenoxy)acetyl]-L-phenylalanyl-L-valine (Compound **5**):

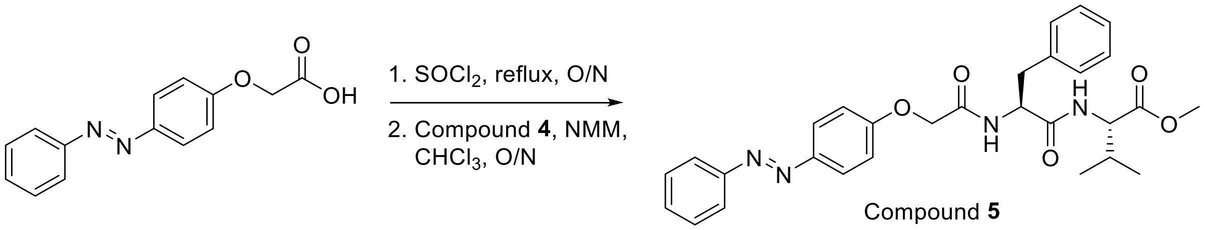

Compound **3** (2.56 g, 10.0 mmol) was refluxed in thionyl chloride (25.0 mL, 343 mmol) for 3 h under nitrogen atmosphere. The excess thionyl chloride was removed via distillation under vacuum to yield a red solid, which was dissolved in chloroform (40 mL) and cooled in an ice bath. A separate solution of compound **4** (4.12 g 10.5 mmol) and *N*-methyl morpholine (1.30 mL, 12.0 mmol) in chloroform (50 mL) was added and the reaction mixture was stirred for 30 minutes. *N*-methyl morpholine (1.30 mL, 12.0 mmol) was added, and the resulting mixture was stirred overnight and allowed to warm to room temperature. The resulting solution was washed with water (200 mL × 2) and brine (200 mL). The separated organic layer was dried over anhydrous magnesium sulfate, which was then removed by filtration, and the solvent removed to give the product as a bright yellow solid. Yield: 4.93 g, 91%. ^{1}H NMR (400 MHz, DMSO–d_6) δ: 8.51 (1H, d, J = 7.5 Hz, NH), 8.27 (1H, d, J = 8.5 Hz, NH), 7.87–7.81 (4H, m, H_{Ar}), 7.61–7.51 (3H, m, H_{Ar}), 7.33–7.18 (5H, m, H_{Ar}), 7.00 (2H, d, J = 9.1 Hz, H_{Ar}), 4.70 (1H, td, J = 9.0, 4.1 Hz, NH–CH), 4.58 (2H, d, J = 2.0 Hz, O–CH_2), 4.40–4.23 (1H, m, NH–CH), 3.63 (3H, s, O–CH_3), 3.07 (1H, dd, J = 14.0, 4.0 Hz, CH_2), 2.86 (1H, dd, J = 14.0, 10.0 Hz, CH_2), 2.10–2.01 (1H, m, CH–CH_3), 0.87 (6H,

dd, *J* = 21.0, 6.5 Hz, CH–C$\underline{H}_3$). ^{13}C NMR (101 MHz, DMSO–d_6) δ: 172.8, 171.1, 166.9, 160.4, 152.0, 146.4, 137.5, 130.9, 129.4, 129.2, 128.1, 126.3, 124.4, 122.3, 115.2, 66.7, 53.2, 51.9, 50.3, 37.5, 24.2, 22.7, 21.3. HRMS [M+Na]$^+$ calculated for [$C_{29}H_{32}N_4NaO_5$]$^+$: 539.2265, found: 539.2279.

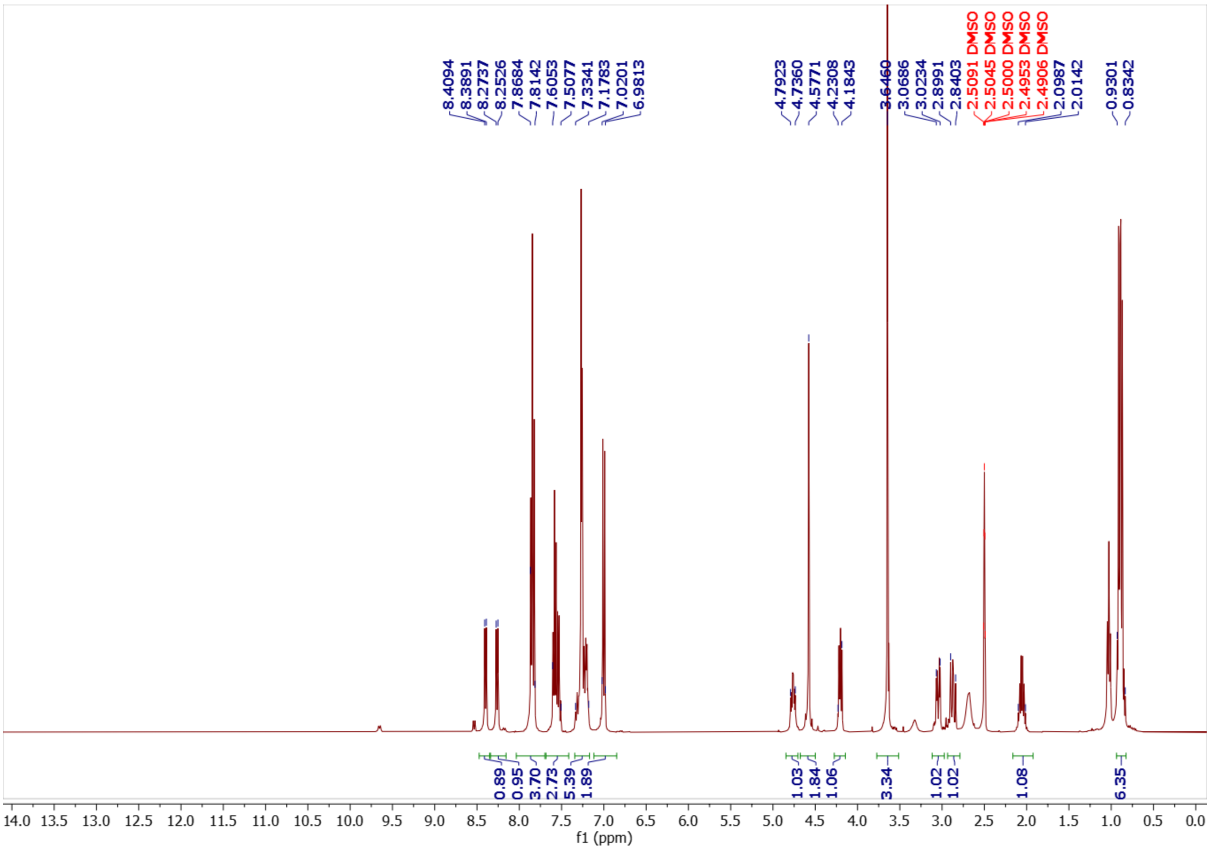

Supplementary Figure 9. ^{1}H NMR spectrum of compound **5** in DMSO–d_6.

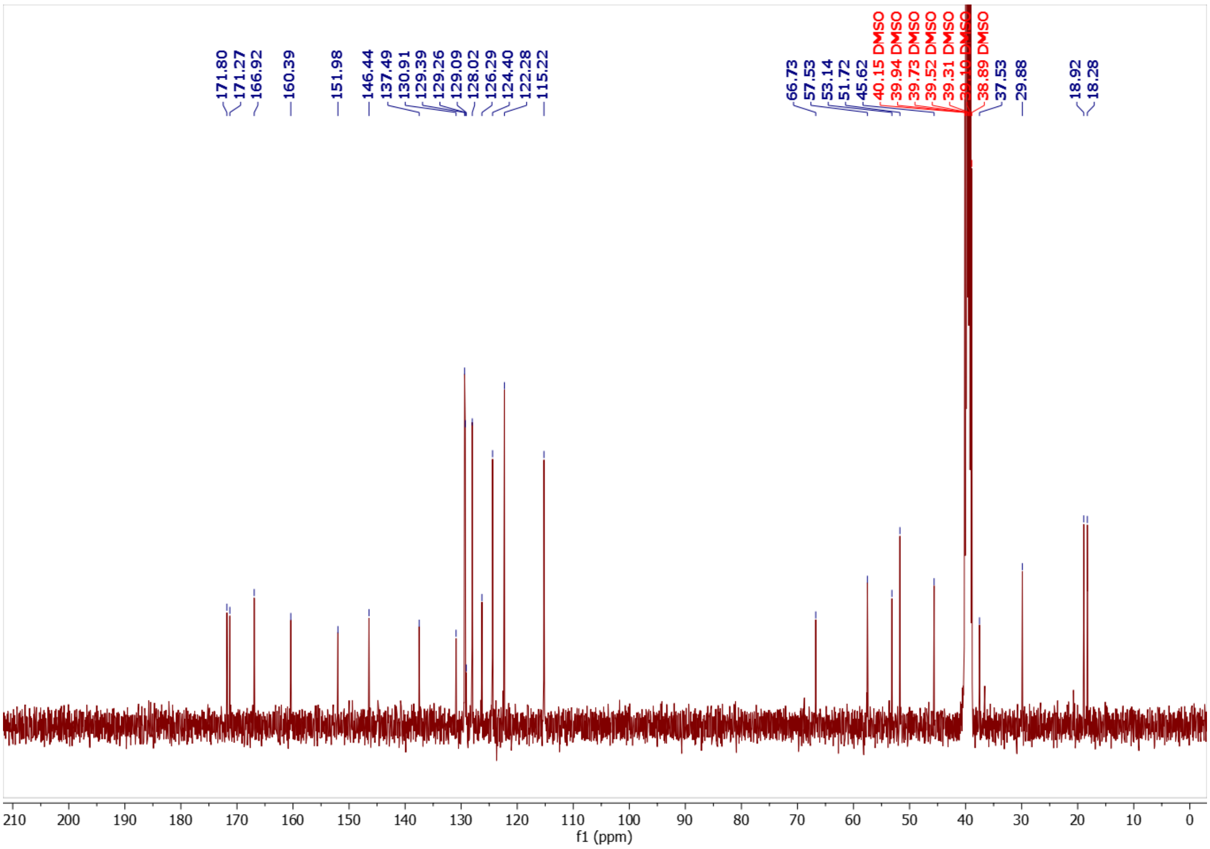

Supplementary Figure 10. ^{13}C NMR spectrum of compound **5** in DMSO–d_6.

Synthesis of [2-(4-{phenyldiazenyl}phenoxy)acetyl]-L-phenylalanyl-L-valine (**Azo-FV**):

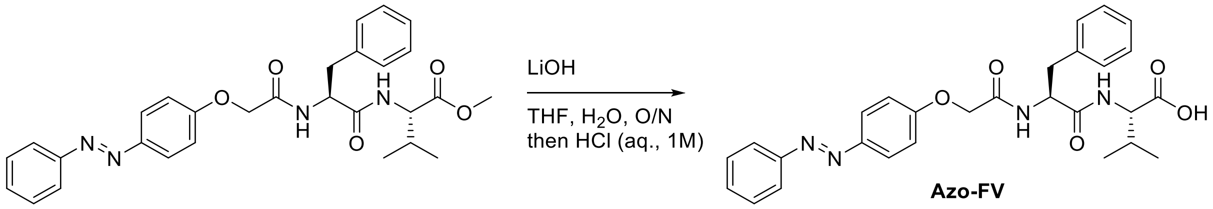

A mixture of compound **5** (2.16 g, 4.19 mmol) and lithium hydroxide (0.4 g, 16.76 mmol) in tetrahydrofuran (30 mL) and water (30 mL) was stirred overnight. The resulting clear solution was poured into aqueous hydrochloric acid (1 M, 100 mL) and stirred for 90 min. The resulting precipitate was collected by filtration, washed with water (300 mL) and stirred in ether overnight, then filtrated and dried in a vacuum oven at 50°C to give the product as an orange solid. Yield: 1.72 g, 82%. ^1H NMR (400 MHz, DMSO–d_6) δ: 12.71 (1H, bs, COOH), 8.27 (2H, d, J = 8.5 Hz, NH), 7.82–7.88 (4H, m, H_{Ar}), 7.61–7.51 (3H, m, H_{Ar}), 7.31–7.18 (5H, m, H_{Ar}), 7.02 (2H, d, J = 9.1 Hz, H_{Ar}), 4.77 (1H, td, J = 9.0, 4.0 Hz, NH–CH), 4.59 (2H, s, O–CH_2), 4.20 (1H, dd, J = 8.5, 6.0 Hz, NH–CH), 3.07 (1H, dd, J = 14.0, 4.0 Hz, CH_2), 2.87 (1H, dd, J = 14.0, 10.0 Hz, CH_2), 2.14–2.06 (1H, m, J = 6.5 Hz, CH–CH_3), 0.90 (6H, dd, J = 7.0, 4.0 Hz, CH_3).^{13}C NMR (101 MHz, DMSO–d_6) δ: 172.9, 171.2, 167.0, 160.4, 152.0, 146.5, 137.6, 130.9, 129.4, 129.3, 128.0, 126.3, 124.4, 122.3, 115.2, 66.8, 57.3, 53.2, 37.6, 29.9, 19.1, 18.1. HRMS $[M+H]^+$ calculated for $[C_{28}H_{31}N_4O_5]^+$: 503.2289, found: 503.2300.

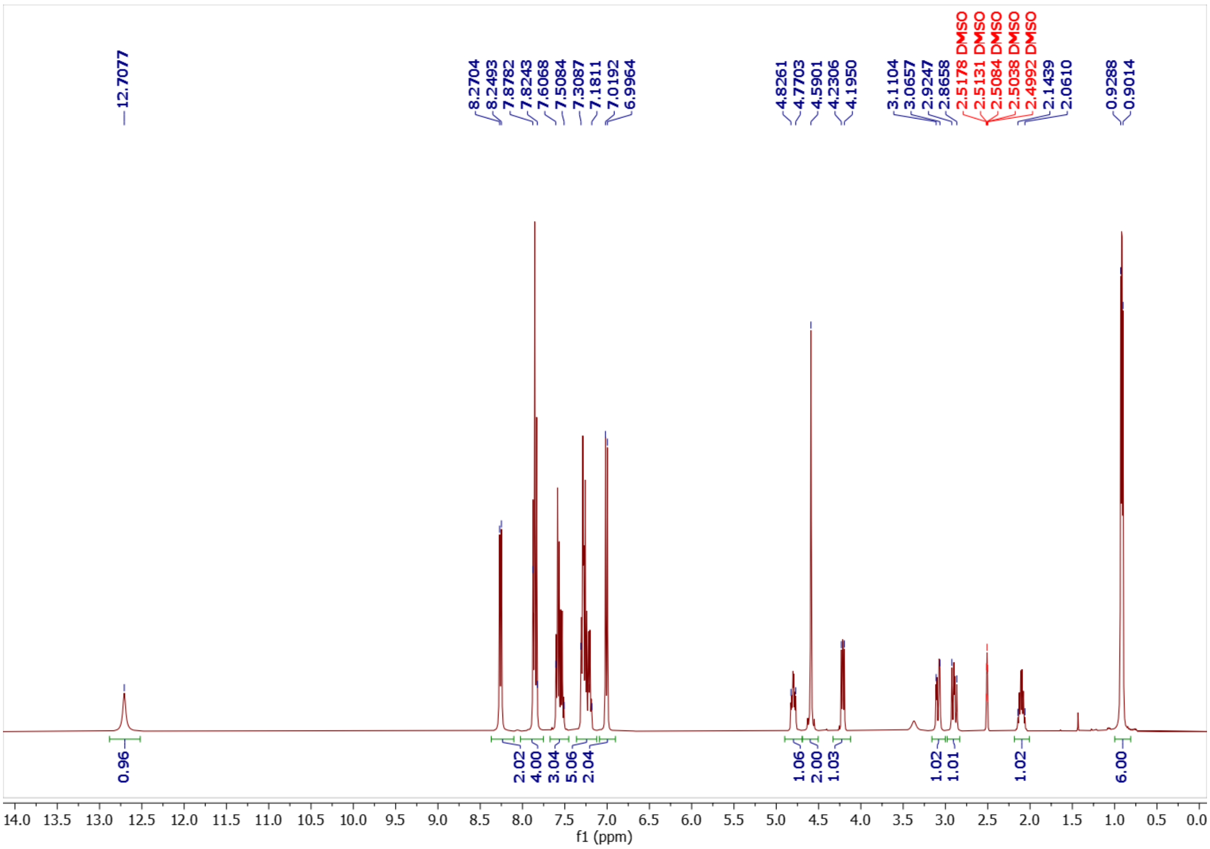

Supplementary Figure 11. ^{1}H NMR spectrum of Azo-FV in DMSO–d_6.

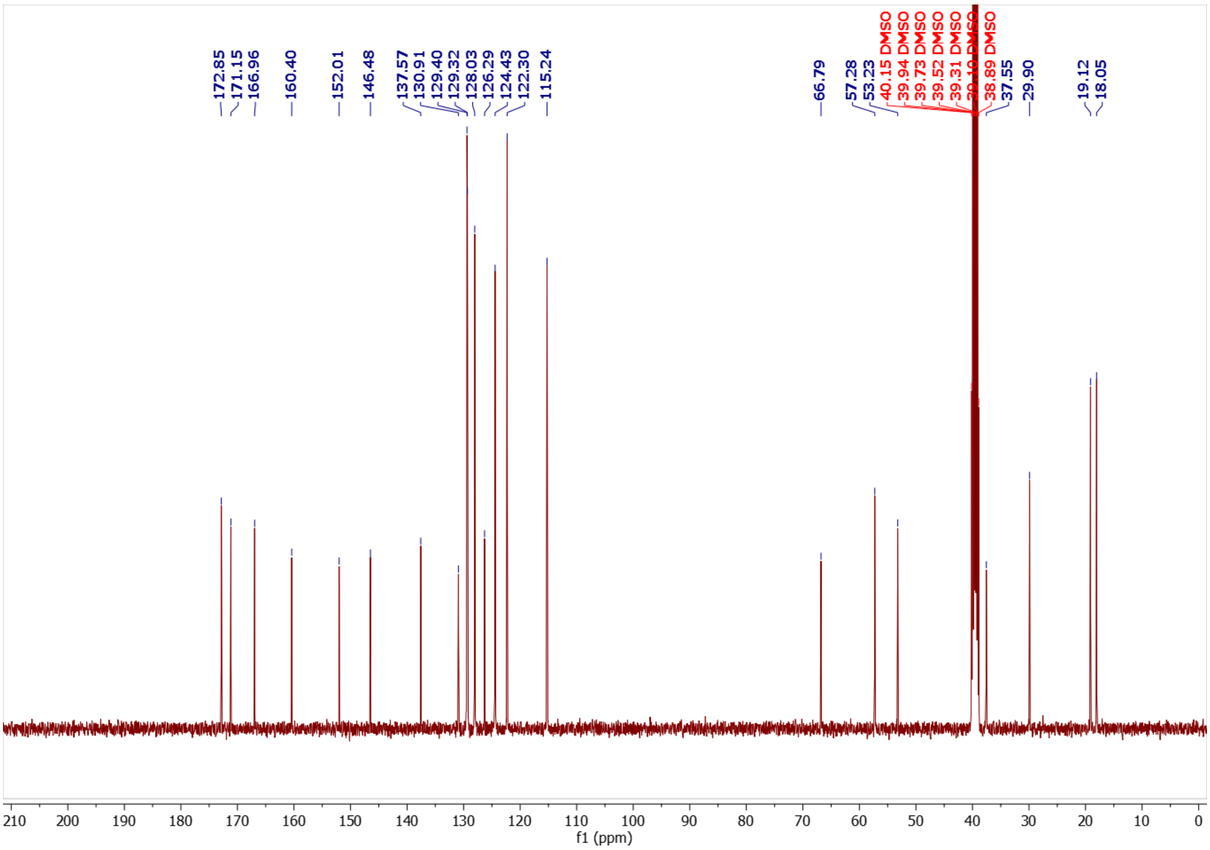

Supplementary Figure 12. ^{13}C NMR spectrum of Azo-FV in DMSO–d_6.

Synthesis of ethyl L-phenylalanyl-L-phenylalanyl TFA salt (Compound **6**).

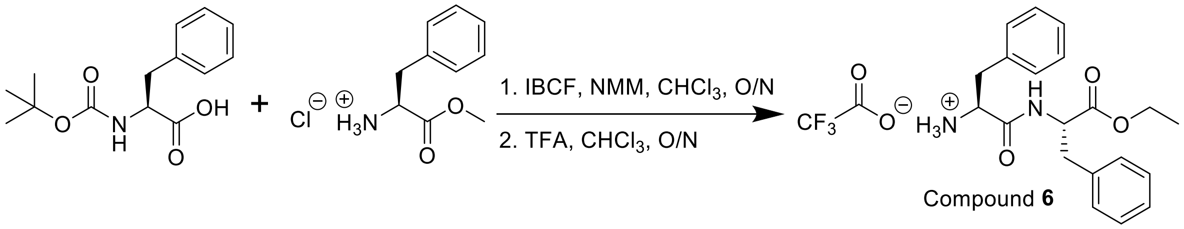

Compound **6**

To a solution of Boc-L-phenylalanine (5.28 g, 19.9 mmol) in chloroform (50 mL) were added isobutyl chloroformate (2.58 mL, 25.87 mmol) and *N*-methyl morpholine (5.40 mL, 39.8 mmol) and the mixture was stirred for 1 h. A separate solution of L-phenylalanyl ethyl ester hydrochloride (5.40 g, 19.9 mmol) and *N*-methyl morpholine (5.40 mL, 39.8 mmol) in chloroform (50 mL) were then added and the mixture was stirred overnight. The now clear solution was diluted with chloroform (70 mL) and washed with aqueous hydrochloric acid (1 M, 200 mL), water (200 mL × 2), and brine (200 mL), dried over anhydrous magnesium sulfate, filtered, and evaporated under reduced pressure to yield an off–white solid. The solid was dissolved in chloroform (40 mL) and trifluoroacetic acid (15 mL, 199 mmol) and the mixture was stirred overnight. The solution was concentrated under reduced pressure to a viscous oil. The oil was dissolved in chloroform (30 mL), poured into diethyl ether (400 mL) and stirred overnight. The precipitate was filtered off, washed in the filter with a few small portions of diethyl ether, and dried to give the product as a white solid. Yield: 6.08 g, 68 %. ^{1}H NMR (400 MHz, DMSO–d_6) δ: 9.07 (1H, d, *J* = 9.1 Hz, N<u>H</u>), 8.2 (3H, bs, N$\underline{H}_3^+$), 7.34–7.22 (10H, m, $\underline{H}_{Ar}$), 4.57 (1H, td, *J* = 8.0, 6.5 Hz, C<u>H</u>), 4.10–4.02 (3H, m, C$\underline{H}_2$ and C<u>H</u>), 3.15–3.92 (4H, m, C$\underline{H}_2$ and C$\underline{H}_2$), 1.11 (3H, td, *J* = 10.0, 7.0 Hz C$\underline{H}_3$), ^{13}C NMR (101 MHz, DMSO–d_6) δ: 170.7, 168.3, 136.7, 134.7, 129.6, 129.1, 128.5, 128.4, 127.2, 126.8, 53.9, 53.1, 36.9, 36.8, 13.9. HRMS [M+H]$^+$ calculated for [$C_{22}H_{26}N_2O_3$]$^+$: 341.1860, found: 341.1862.

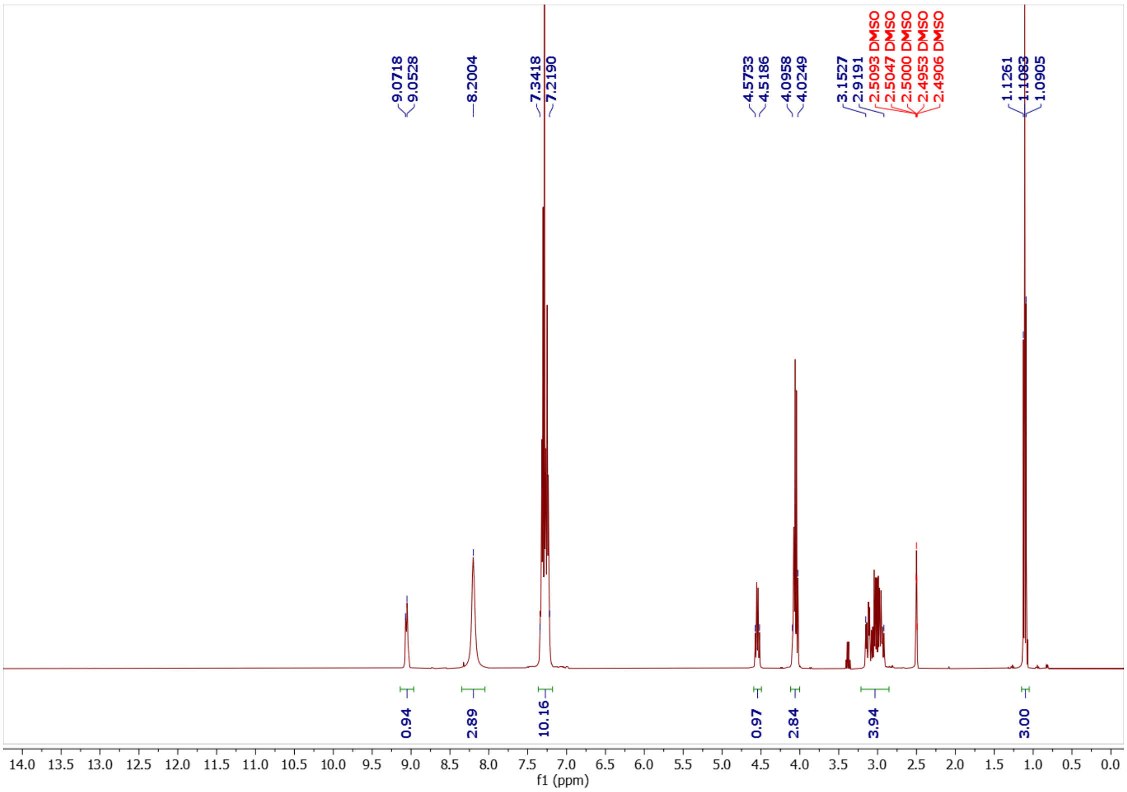

Supplementary Figure 13. ^{1}H NMR spectrum of Compound **6** in DMSO–d_6.

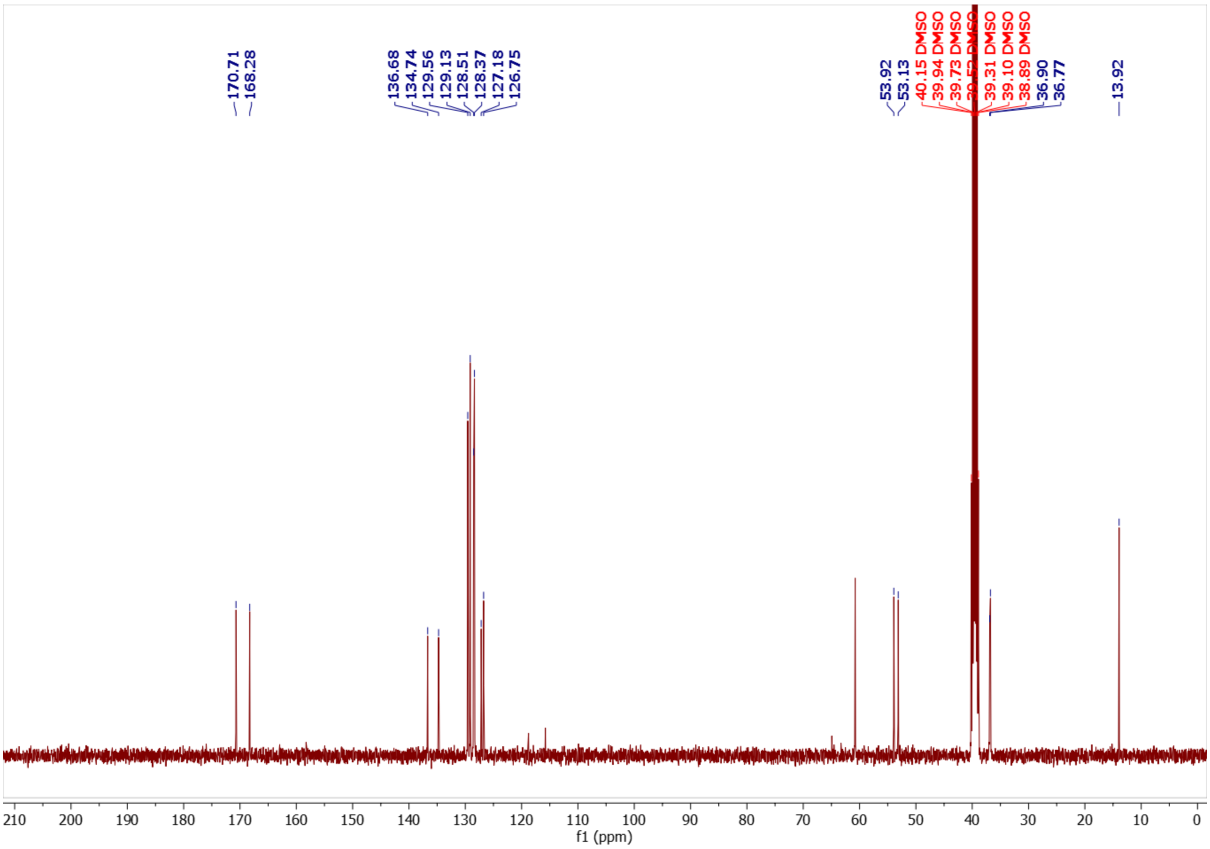

Supplementary Figure 14. ^{13}C NMR spectrum of compound **6** in DMSO–d_6.

Synthesis of ethyl [2-(4-{phenyldiazenyl}phenoxy)acetyl]-L-phenylalanyl-L-phenylalanine (Compound **7**):

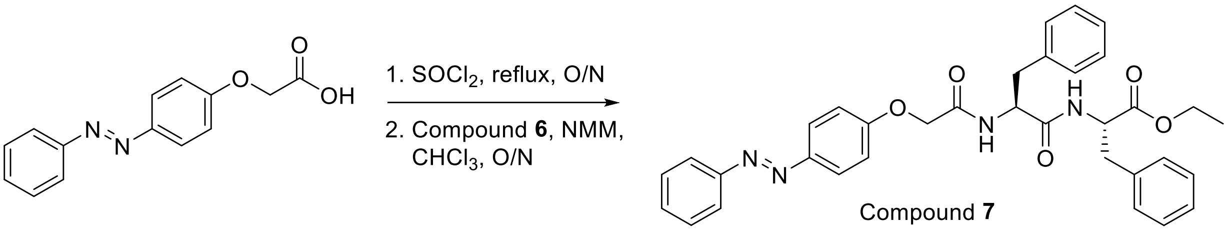

Compound **3** (1.0 g, 3.9 mmol) was refluxed with thionyl chloride (10.0 mL, 51.5 mmol) for 3 h under nitrogen atmosphere. The excess thionyl chloride was removed via distillation under vacuum to yield a red solid, which was dissolved in chloroform (30 mL) and cooled in an ice bath. A separate solution of compound **7** (1.71 g 3.90 mmol) and *N*-methyl morpholine (2.70 mL, 17.6 mmol) in chloroform (30 mL) was added and the reaction mixture was stirred for 30 min. *N*-methyl morpholine (2.70 mL, 17.6 mmol) was added and the resulting mixture was stirred overnight and allowed to warm to room temperature. Then resulting solution was washed with water (200 mL × 2) and brine (200 mL). The separated organic layer was dried over anhydrous magnesium sulfate, which was then removed by filtration, and the solvent removed to give the product as a bright yellow solid. Yield: 1.83 g, 90%. ^{1}H NMR (400 MHz, DMSO–d_6) δ: 8.61 (1H, d, J = 7.5 Hz, N<u>H</u>), 8.23 (1H, d, J = 8.7 Hz, N<u>H</u>), 7.87–7.82 (4H, m, <u>H</u>$_{Ar}$), 7.60–7.51 (3H, m, <u>H</u>$_{Ar}$), 7.29–7.18 (10H, m, <u>H</u>$_{Ar}$), 6.99 (2H, d, J = 11.0 Hz, <u>H</u>$_{Ar}$), 4.66 (1H, td, J = 9.3, 4.2 Hz, NH–C<u>H</u>), 4.58–4.64 (3H, m, O–C<u>H</u>$_2$–C=O and NH–C<u>H</u>), 4.03 (2H, q, J = 7.0 Hz C<u>H</u>$_2$–CH$_3$), 3.07–2.94 (3H, m, J = 14.0, 4.0 Hz, CH–C<u>H</u>$_2$), 2.82 (1H, dd, J = 13.8, 9.9 Hz, CH–C<u>H</u>$_2$), 1.12–1.08 (3H, td,

J = 7.1, 2.0 Hz C<u>H</u>$_3$). ^{13}C NMR (101 MHz, DMSO–d_6) δ: 171.2, 171.0, 166.8, 160.4, 152.0, 146.4, 137.5, 137.0, 130.9, 129.4, 129.2, 129.1, 128.2, 128.0, 126.5, 126.3, 124.4, 122.3, 115.2, 66.7, 60.5, 53.7, 53.2, 37.5, 36.7, 13.9. HRMS [M+H]$^+$ calculated for [$C_{34}H_{35}N_4O_5$]$^+$: 579.2602, found: 579.2613.

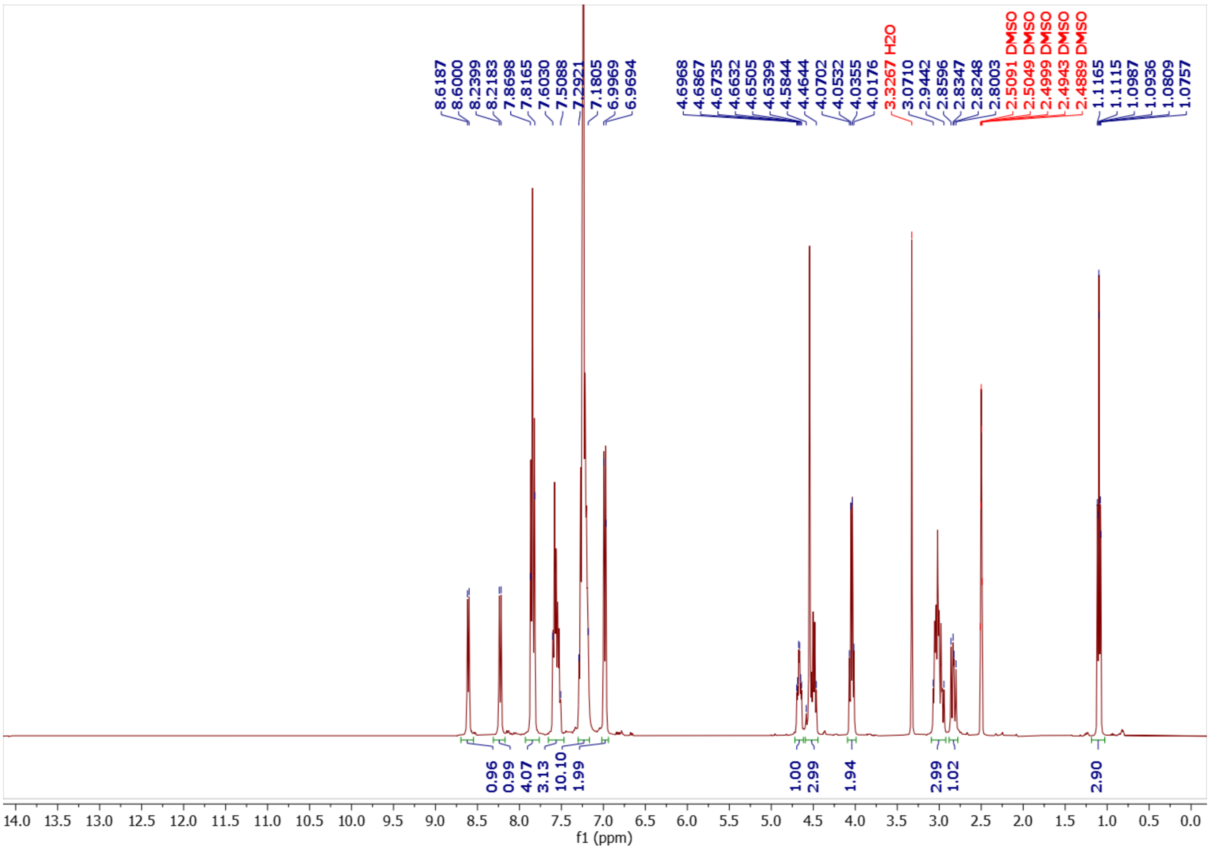

Supplementary Figure 15. ^{1}H NMR spectrum of compound **7** in DMSO–d_6.

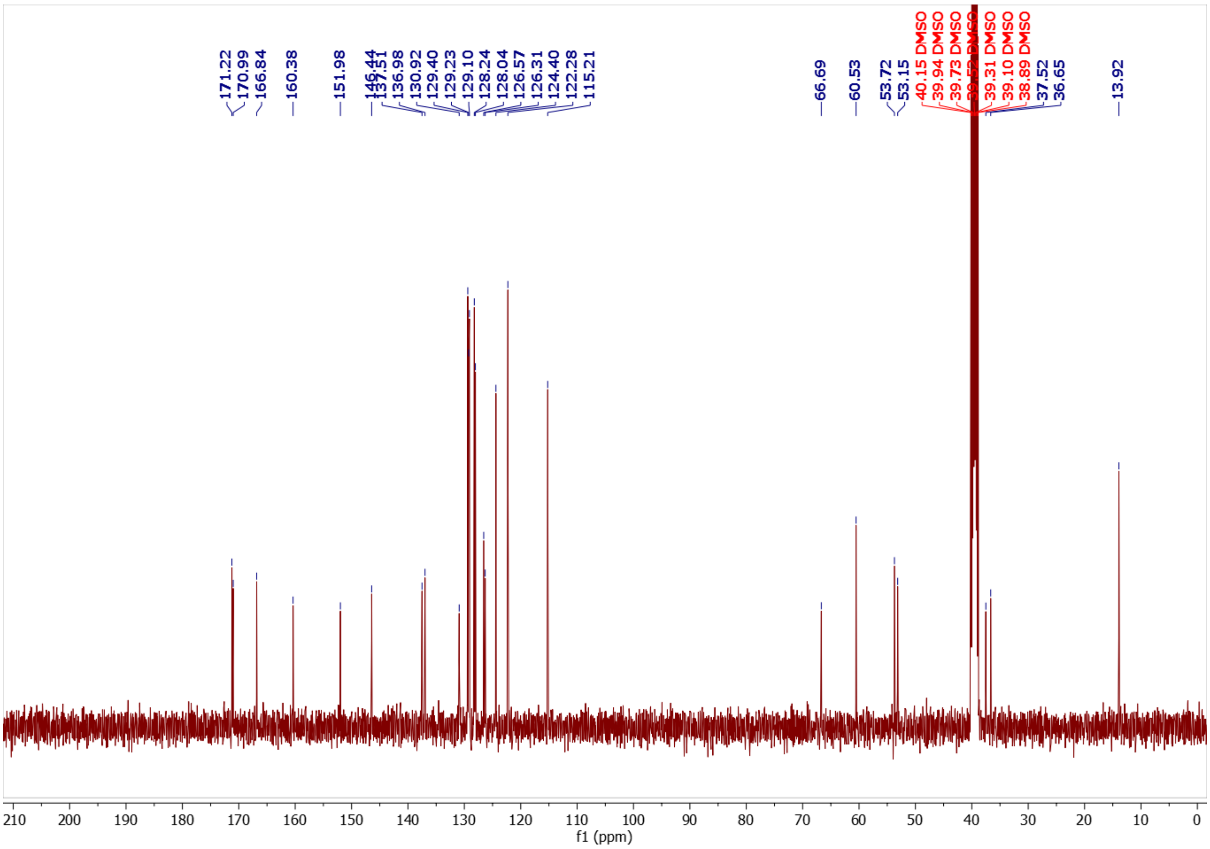

Supplementary Figure 16. ^{13}C NMR spectrum of compound **7** in DMSO–d_6.

Synthesis of [2-(4-{phenyldiazenyl}phenoxy)acetyl]-L-phenylalanyl-L-phenylalanine (**Azo-FF**):

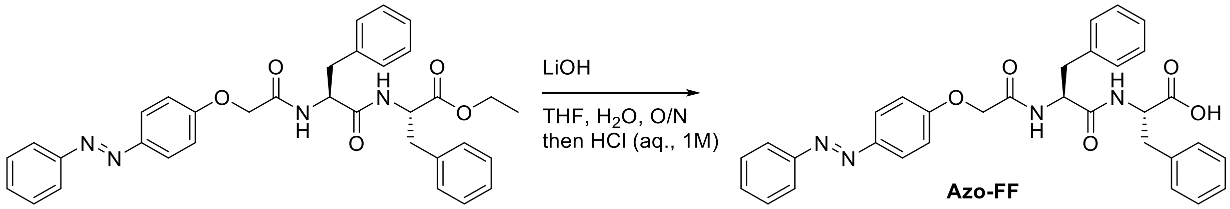

A mixture of compound **7** (2.16 g, 4.19 mmol) and lithium hydroxide (0.40 g, 16.8 mmol) in tetrahydrofuran (30 mL) and water (30 mL) was stirred overnight. The resulting clear solution was poured into aqueous hydrochloric acid (1 M, 100 mL) and stirred for 90 min. The resulting precipitate was collected by filtration, washed with water (300 mL) and stirred in ether overnight, then filtrated and dried in a vacuum oven at 50°C to give the product as an orange solid. Yield: 1.72 g, 82%. ^{1}H NMR (400 MHz, DMSO–d_6) δ: 12.80 (1H, bs, COOH), 8.43 (1H, d, J = 8.0 Hz, NH), 8.19 (1H, d, J = 8.5 Hz, NH), 7.81–7.87 (4H, m, H_{Ar}), 7.61–7.51 (3H, m, H_{Ar}), 7.29–7.17 (10H, m, J = 8.0 Hz, H_{Ar}), 6.99–6.96(2H, m, H_{Ar}), 4.65 (1H, td, J = 9.3, 4.1 Hz, NH–CH), 4.54 (2H, s, O–CH_2), 4.50–4.45 (1H, m, NH–CH), 3.12–3.01 (2H, m, CH_2), 2.94 (1H, dd, J = 13.9, 8.8 Hz, CH_2), 2.82 (1H, dd, J = 13.8, 9.9 Hz, CH_2). ^{13}C NMR (101 MHz, DMSO–d_6) δ: 172.7, 170.9, 166.8, 160.4, 152.0, 146.5, 137.6, 137.4, 130.9, 129.4, 129.3, 129.1, 128.2, 128.0, 126.5, 126.3, 124.4, 122.3, 115.2, 66.7, 53.5, 53.2, 37.5, 36.7. HRMS [M+H]$^+$ calculated for $[C_{28}H_{31}N_4O_5]^+$: 551.2289, found: 551.2297.

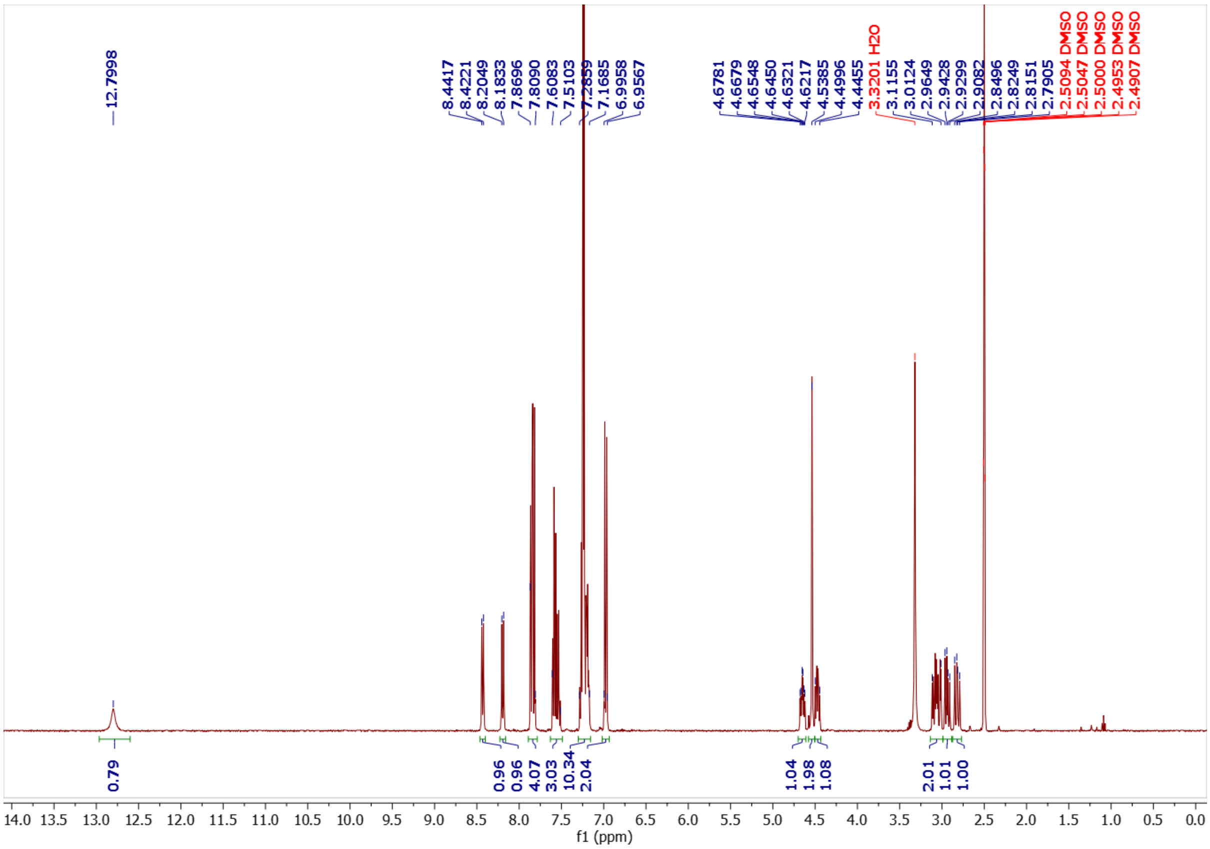

Supplementary Figure 17. ^{1}H NMR spectrum of Azo-FF in DMSO–d_6.

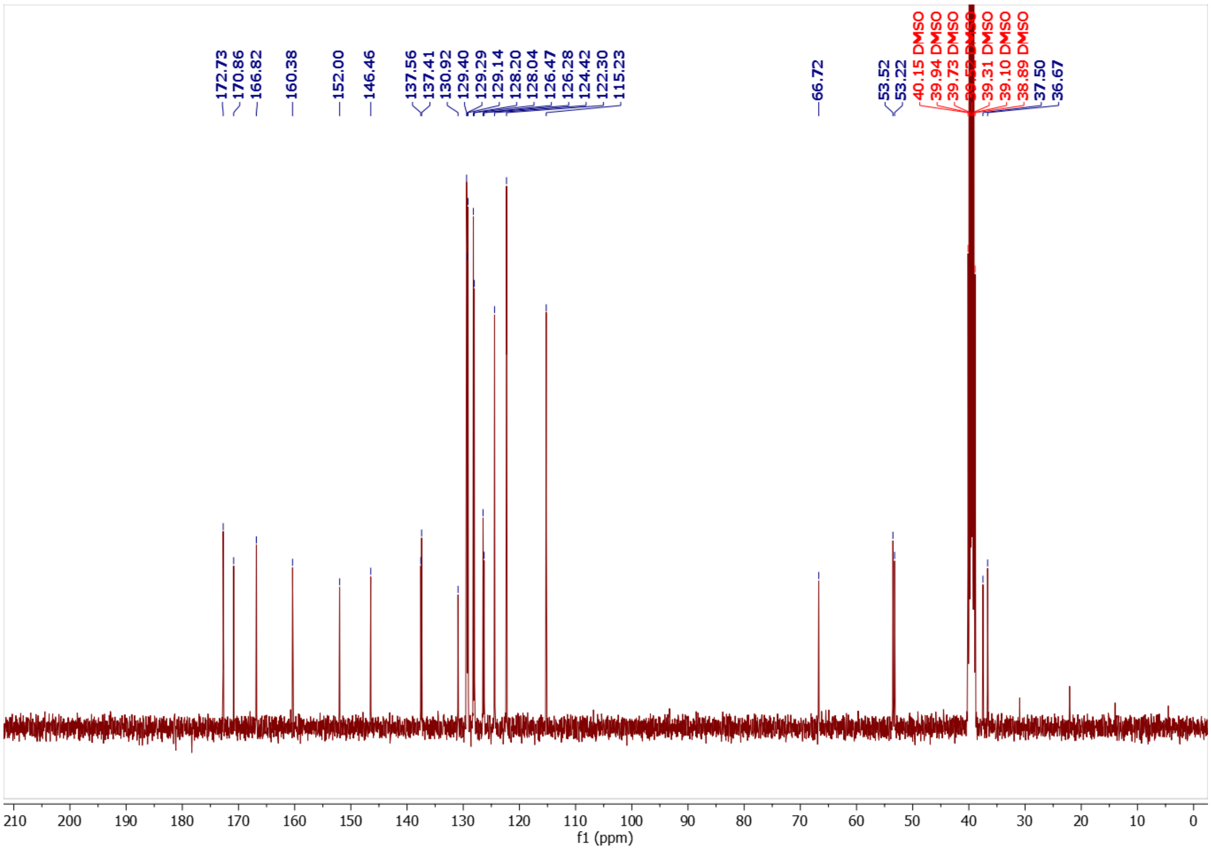

Supplementary Figure 18. ^{13}C NMR spectrum of Azo-FF in DMSO–d_6.

Synthesis of methyl L-phenylalanyl-L-leucinate TFA salt (Compound **8**):

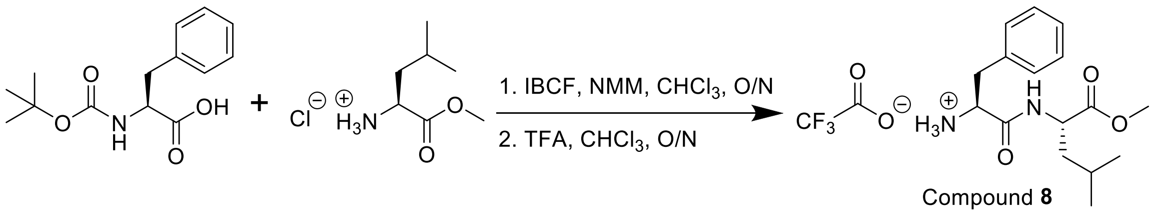

To a solution of Boc–L–phenylalanine (3.0 g, 11.3 mmol) in chloroform (30 mL) were added isobutyl chloroformate (1.56 mL, 14.7 mmol) and *N*-methyl morpholine (2.60 mL, 22.6 mmol) and the mixture was stirred for 1 h. A separate solution of L-leucine methyl ester hydrochloride (2.05 g, 11.3 mmol) and *N*-methyl morpholine (2.60 mL, 22.6 mmol) in chloroform (30 mL) were then added and the mixture was stirred overnight. The now clear solution was diluted with chloroform (50 mL) and washed with aqueous hydrochloric acid (1 M, 200 mL), water (200 mL × 2), and brine (200 mL), dried over anhydrous magnesium sulfate, filtered, and evaporated under reduced pressure to yield an off–white solid. The solid was dissolved in chloroform (40 mL) and trifluoroacetic acid (8.70 mL, 113 mmol) and the mixture was stirred overnight. The solution was concentrated under reduced pressure to a viscous oil. The oil was dissolved in chloroform (30 mL), poured into diethyl ether (400 mL), and stirred overnight. The precipitate was filtered off, washed in the filter with a few small portions of diethyl ether and dried to give the product as a white solid. Yield: 3.47 g, 81%. ^{1}H NMR (400 MHz, DMSO–d_6) δ: 8.89 (1H, d, J = 8.3 Hz, N$\underline{H}$), 8.22 (3H, bs, N$\underline{H}_3^+$), 7.35–7.26 (5H, m, $\underline{H}_{Ar}$), 4.34 (1H, ddd, J = 8.9, 8.2, 6.2 Hz, C$\underline{H}$), 4.07 (1H, dd, J = 8.2, 6.3 Hz, C$\underline{H}$), 3.62 (3H, s, O–C$\underline{H}_3$), 3.11 (1H, dd, J = 14.0, 5.8 Hz, C$\underline{H}_2$), 2.95 (1H, dd, J = 14.0, 7.8 Hz, C$\underline{H}_2$), 1.68–1.48 (3H, m, C$\underline{H}_2$–C$\underline{H}$–CH_3), 0.90 (6H, dd, J = 16.7, 6.4 Hz, C$\underline{H}_3$). ^{13}C NMR (101 MHz, DMSO–d_6) δ: 172.4, 168.4, 135.0, 129.7, 128.7, 127.4, 53.4, 52.3, 50.6, 37.1, 24.2, 22.9. 21.5. HRMS $[M+H]^+$ calculated for $[C_{16}H_{26}N_2O_3]^+$: 293.1860, found: 293.1869.

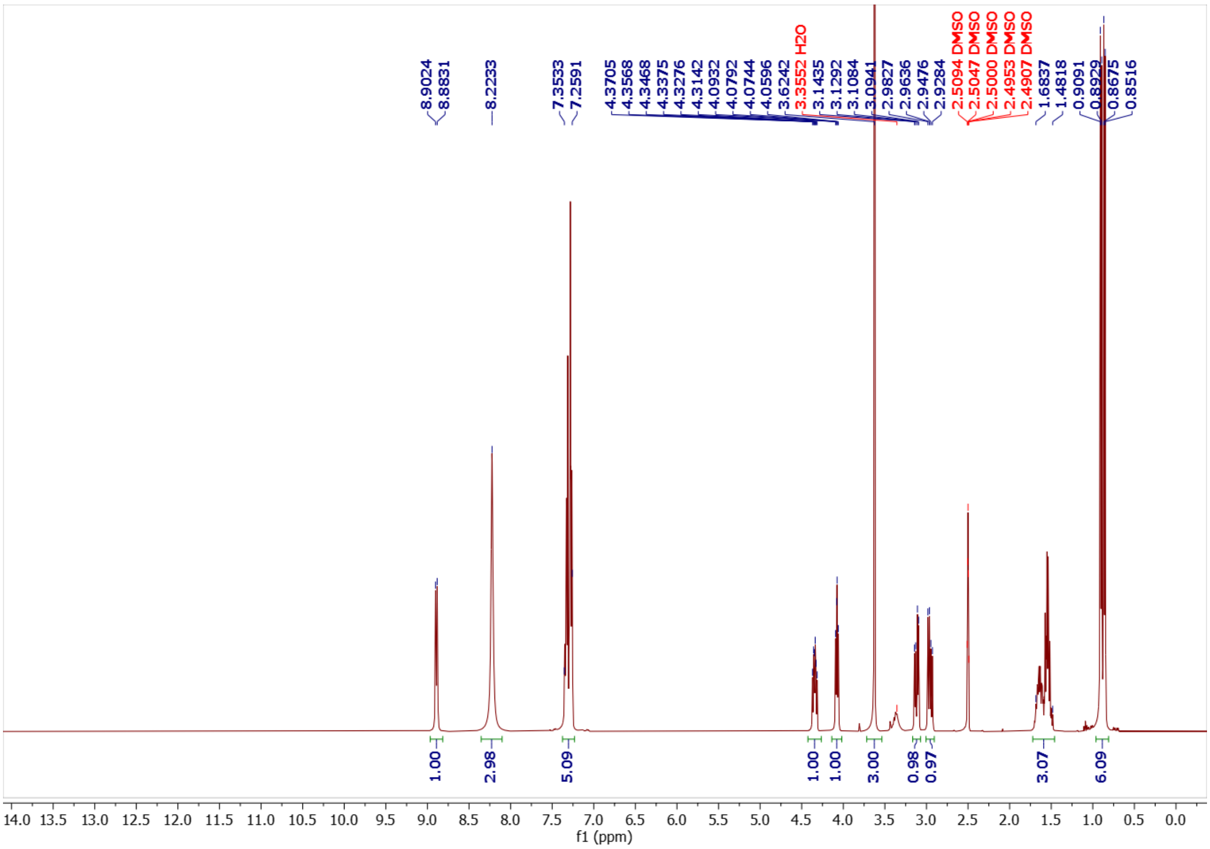

Supplementary Figure 19. ^{1}H NMR spectrum of compound **10** in DMSO–*d*$_6$.

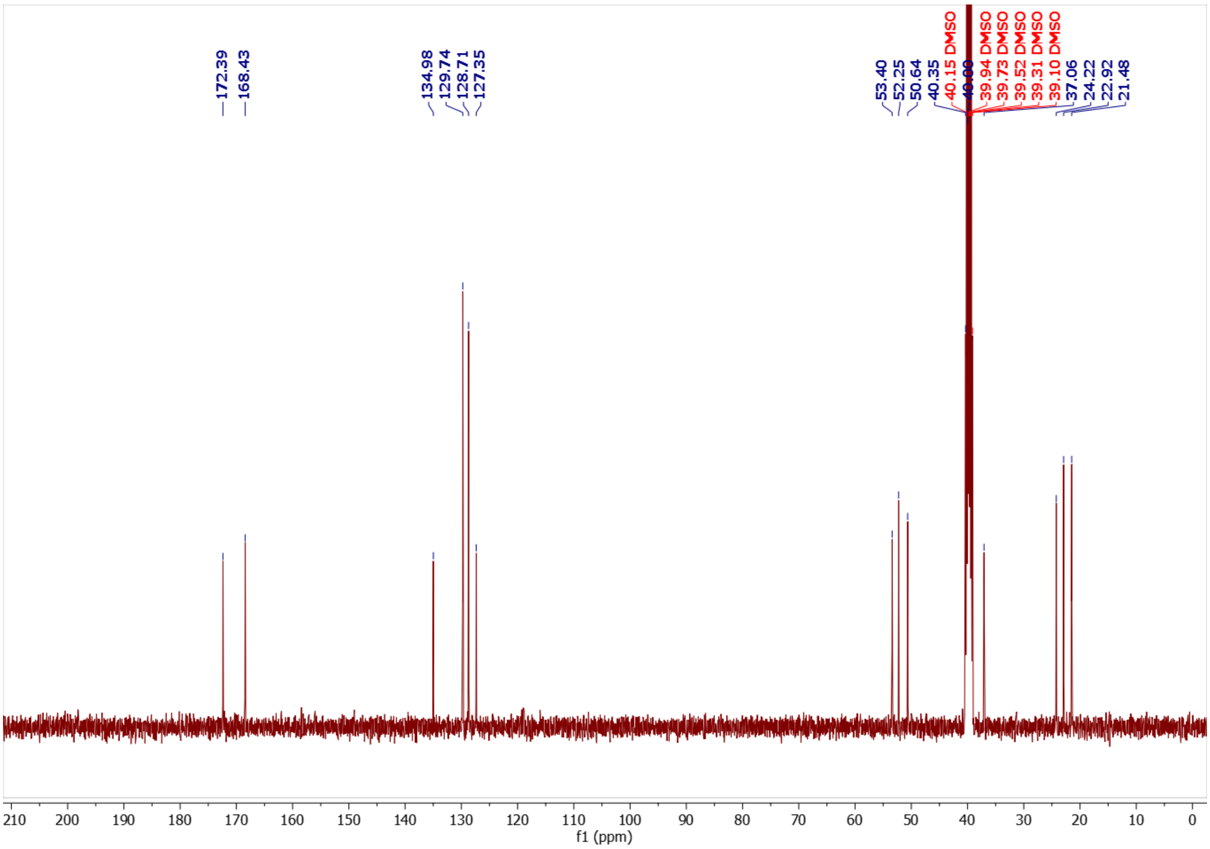

Supplementary Figure 20. ^{13}C NMR spectrum of compound **8** in DMSO–*d*$_6$.

Synthesis of methyl [2-(4-{phenyldiazenyl}phenoxy)acetyl]-L-phenylalanyl-L-leucinate (Compound **9**):

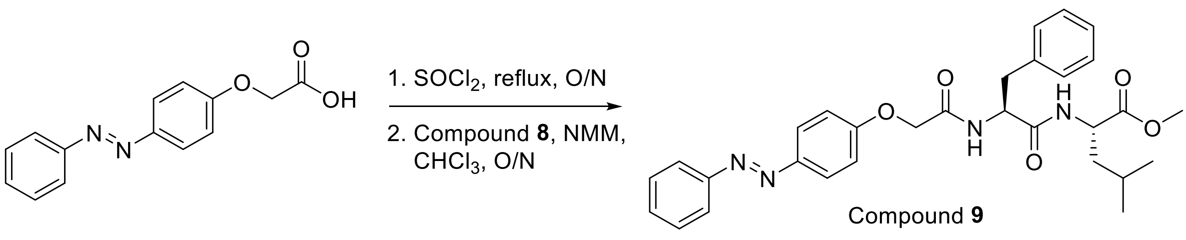

Compound **3** (2.56 g, 10.0 mmol) was refluxed with thionyl chloride (25.0 mL, 343 mmol) for 3 h under nitrogen atmosphere. The excess thionyl chloride was removed via distillation under vacuum to yield a red solid, which was dissolved in chloroform (40 mL) and cooled in an ice bath. A separate solution of compound **8** (4.24 g 10.5 mmol) and *N*-methyl morpholine (1.30 mL, 12.0 mmol) in chloroform (50 mL) was added and the reaction mixture was stirred for 30 min. *N*-methyl morpholine (1.30 mL, 12.0 mmol) was added, and the resulting mixture was stirred overnight and allowed to warm to room temperature. The resulting solution was washed with water (200 mL × 2) and brine (200 mL). The separated organic layer was dried over anhydrous magnesium sulfate, which was then removed by filtration, and the solvent removed in vacuo to give the product as a bright yellow solid. Yield: 4.82 g, 91%. ^{1}H NMR (400 MHz, DMSO–d_6) δ: 8.50 (1H, d, J = 7.5 Hz, NH), 8.26 (1H, d, J = 8.5 Hz, NH), 7.87–7.81 (4H, m, H_{Ar}), 7.61–7.51 (3H, m, H_{Ar}), 7.27–7.18 (5H, m, H_{Ar}), 7.00 (2H, d, J = 9.0 Hz, H_{Ar}), 4.67 (1H, td, J = 9.2, 4.2 Hz, NH–CH), 4.58 (2H, d, J = 2.0 Hz, O–CH_2), 4.40–4.23 (1H, m, NH–CH), 3.63 (3H, s, O–CH_3), 3.07 (1H, dd, J = 14.0, 4.0 Hz, CH_2), 2.86 (1H, dd, J = 14.0, 10.0 Hz, CH_2), 1.66–1.48 (3H, m, CH and CH_2), 0.90 (3H, d, J = 6.5 Hz, CH_3), 0.84 (3H, d, J = 6.5 Hz, CH_3). ^{13}C NMR (101 MHz, DMSO–d_6) δ: 172.8, 171.1, 166.9, 160.4, 152.0, 146.4, 137.5, 130.9, 129.4, 129.2, 128.1, 126.3, 124.4, 122.3, 115.2, 66.7, 53.2, 51.9, 50.3, 37.5, 24.2, 22.7, 21.3. HRMS $[M+Na]^+$ calculated for $[C_{29}H_{32}N_4NaO_5]^+$: 533.2421, found: 553.2434.

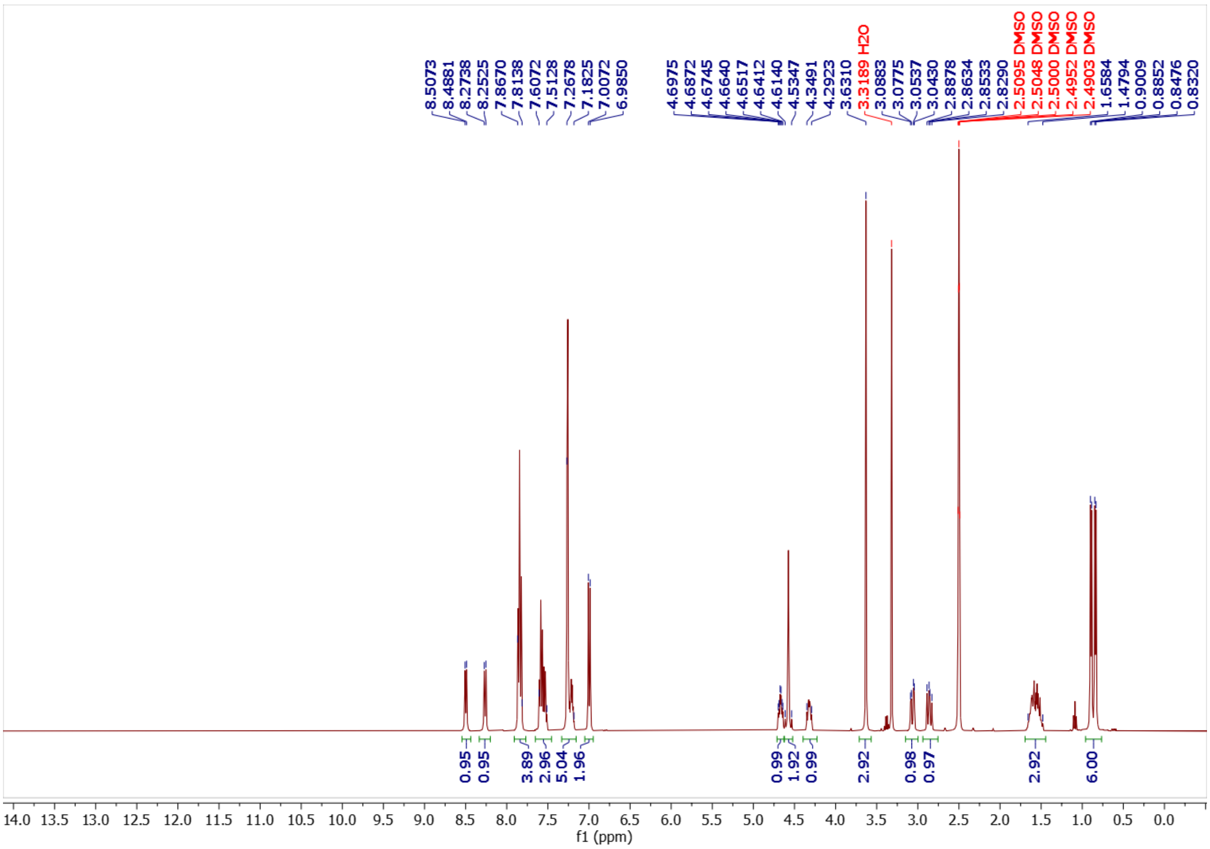

Supplementary Figure 21. ^{1}H NMR spectrum of compound **9** in DMSO–d_6.

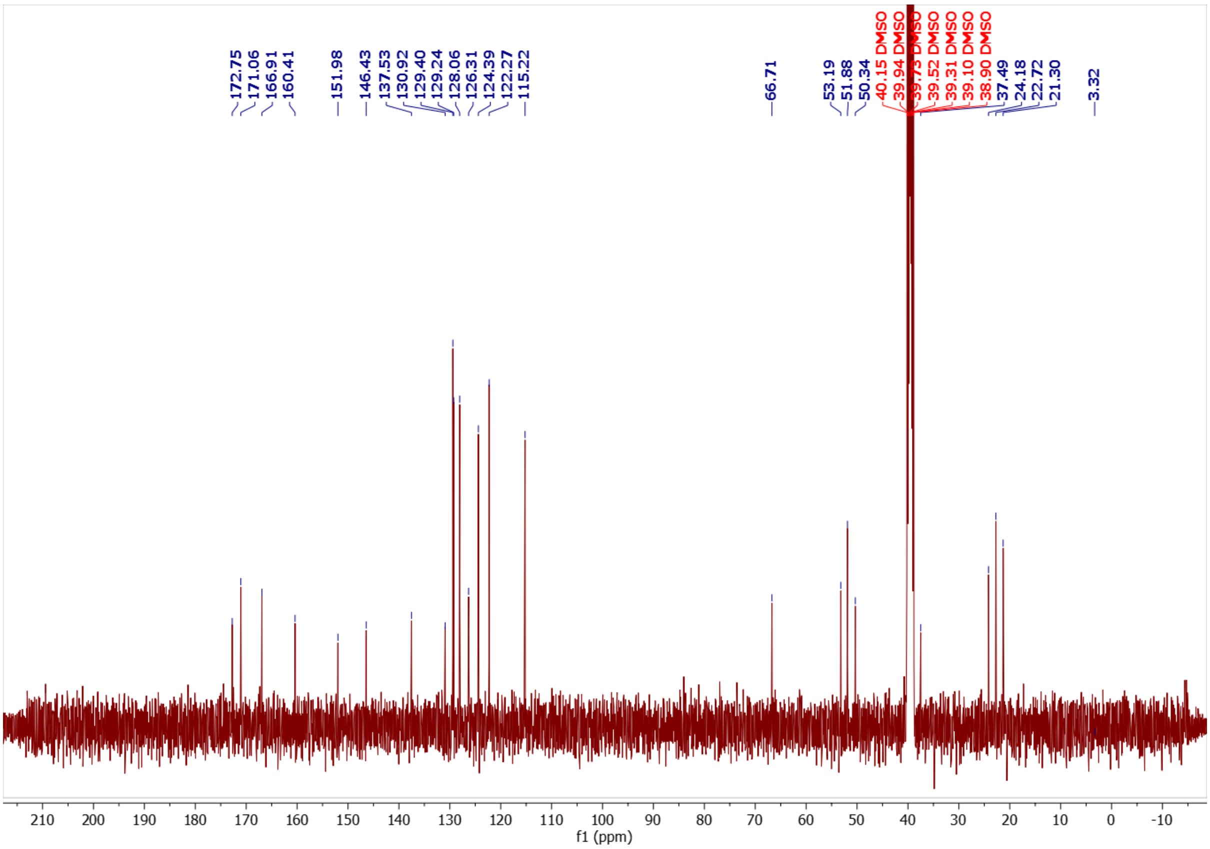

Supplementary Figure 22. ^{13}C NMR spectrum of compound **9** in DMSO–d_6.

Synthesis of [2-(4-{phenyldiazenyl}phenoxy)acetyl]-L-phenylalanyl-L-leucine (**Azo-FL**):

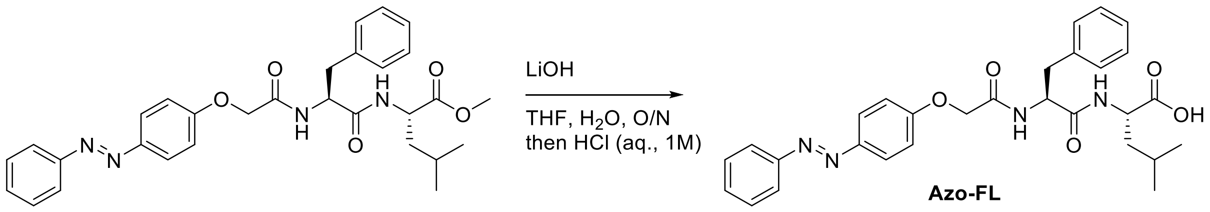

A mixture of compound **9** (3.71 g, 7.0 mmol) and lithium hydroxide (0.67 g, 28.0 mmol) in tetrahydrofuran (30 mL) and water (30 mL) was stirred overnight. The resulting clear solution was poured into aqueous hydrochloric acid (1 M, 250 mL) and stirred for 90 min. The resulting precipitate was collected by filtration, washed with water (300 mL) and stirred in ether overnight, then filtrated and dried in a vacuum oven at 50°C to give the product as an orange solid. Yield: 3.22 g, 89%. ^{1}H NMR (400 MHz, DMSO–d_6) δ: 12.63 (1H, bs, COOH), 8.38 (1H, d, J = 8.0 Hz, NH), 8.24 (1H, d, J = 8.6 Hz, NH), 7.87–7.82 (4H, m, H_{Ar}), 7.60–7.51 (3H, m, H_{Ar}), 7.29–7.18 (5H, m, H_{Ar}), 6.99 (2H, d, J = 8.9 Hz, H_{Ar}), 4.68 (1H, td, J = 9.3, 4.0 Hz, NH–CH), 4.58 (2H, s, O–CH_2), 4.30–4.24 (1H, m, NH–CH), 3.09 (1H, dd, J = 14.0, 4.0 Hz, CH_2), 2.86 (1H, dd, J = 14.0, 10.0 Hz, CH_2), 1.68–1.60 (1H, m, CH), 1.58–1.53 (2H, m, CH_2), 0.90 (3H, d, J = 6.5 Hz, CH_3), 0.85 (3H, d, J = 6.5 Hz, CH_3). ^{13}C NMR (101 MHz, DMSO–d_6) δ: 173.9, 170.9, 166.9, 160.4, 152.0, 146.4, 137.6, 130.9, 129.4, 129.3, 128.0, 126.3, 124.4, 122.3, 115.2, 66.7, 53.2, 50.3, 37.5, 24.3, 22.8, 21.3. HRMS $[M+H]^+$ calculated for $[C_{29}H_{33}N_4O_5]^+$: 517.2445, found: 517.2456.

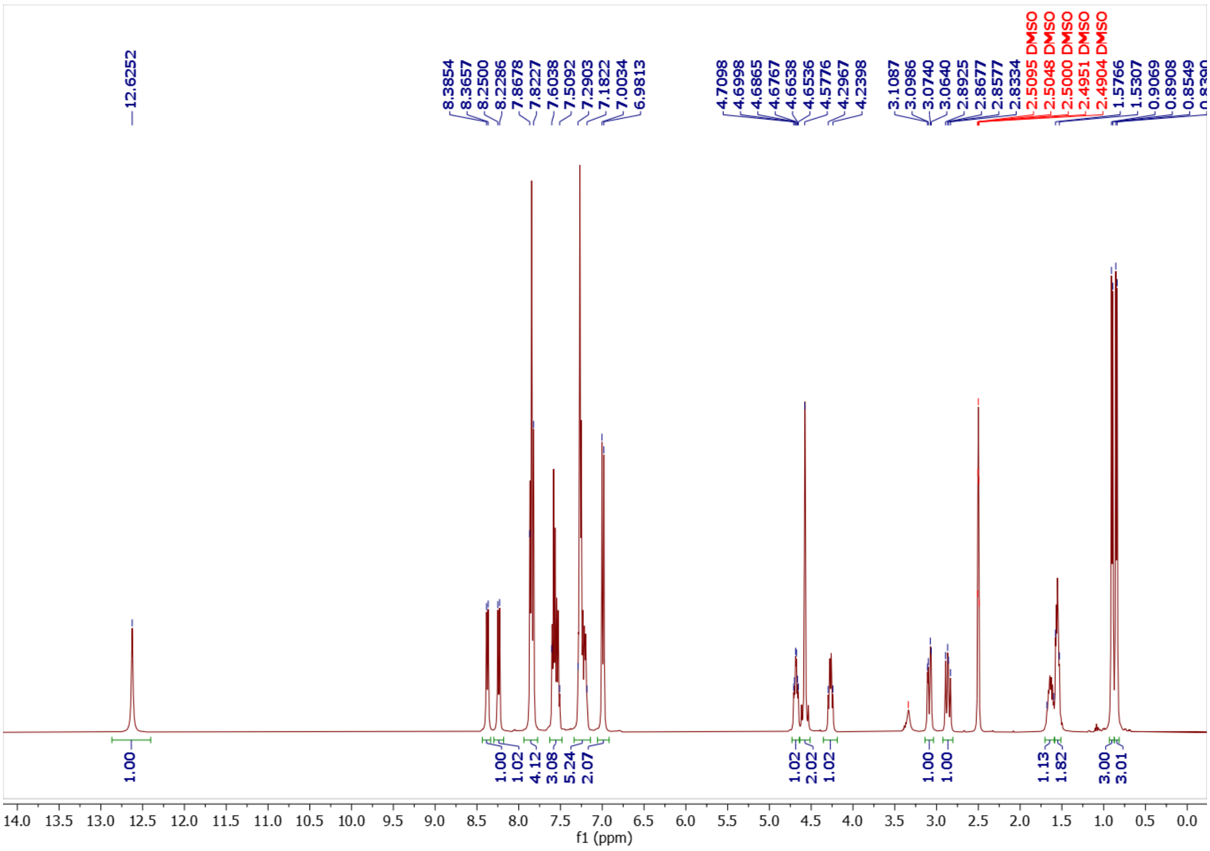

Supplementary Figure 23. ^{1}H NMR spectrum of Azo-FL in DMSO–d_6.

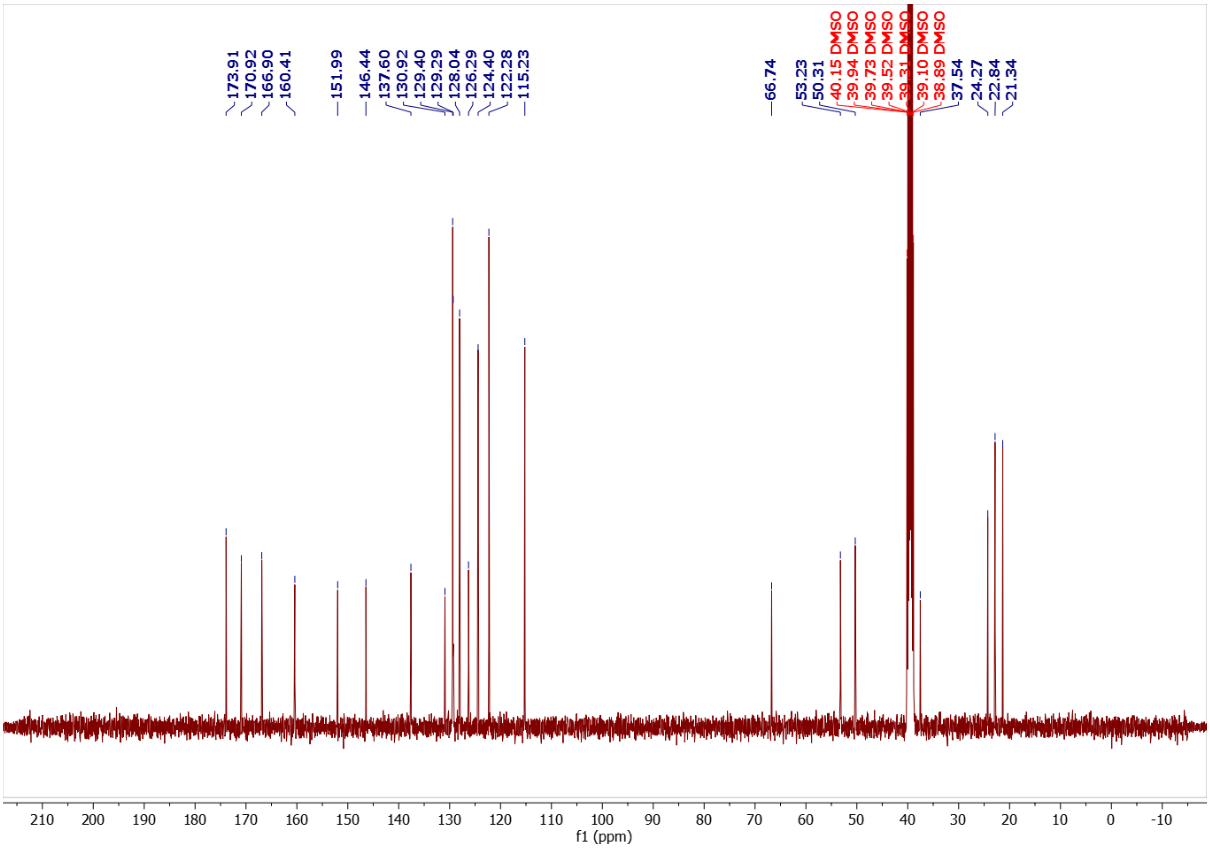

Supplementary Figure 24. ^{13}C NMR spectrum of Azo-FL in DMSO–*d*$_6$.

Synthesis of methyl L-phenylalanyl-L-isoleucinate TFA salt (Compound **10**):

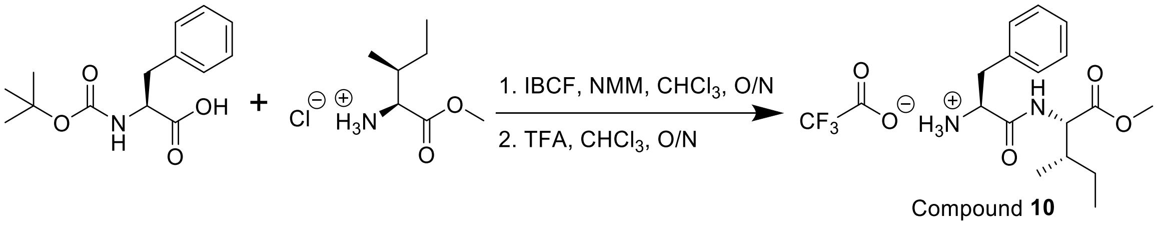

Compound **10**

To a solution of Boc-L-phenylalanine (3.0 g, 11.3 mmol) in chloroform (30 mL) were added isobutyl chloroformate (1.56 mL, 14.7 mmol) and *N*-methyl morpholine (2.60 mL, 22.6 mmol) and the mixture was stirred for 1 h. A separate solution of L-isoleucine methyl ester hydrochloride (2.05 g, 11.3 mmol) and *N*-methyl morpholine (2.60 mL, 22.6 mmol) in chloroform (30 mL) was added and the mixture was stirred overnight. The now clear solution was diluted with chloroform (50 mL) and washed with aqueous hydrochloric acid (1 M, 200 mL), water (200 mL × 2), and brine (200 mL), dried over anhydrous magnesium sulfate, filtered, and evaporated under reduced pressure to yield an off–white solid. The solid was dissolved in chloroform (40 mL) was trifluoroacetic acid (8.70 mL, 113 mmol) and the mixture was stirred overnight. The solution was concentrated under reduced pressure to a viscous oil. The oil was dissolved in chloroform (30 mL), poured into diethyl ether (400 mL) and stirred overnight. The precipitate was filtered off, washed in the filter with a few small portions of diethyl ether and dried to give the product as a white solid. Yield: 3.47 g, 81%. ^{1}H NMR (400 MHz, DMSO–*d*$_6$) δ: 8.74 (1H, d, J = 8.3 Hz, N$\underline{H}$), 8.23 (3H, bs, N$\underline{H}_3^+$), 7.34–7.23 (5H, m, $\underline{H}_{Ar}$), 4.25 (1H, t, J = 7.5 Hz, C$\underline{H}$), 4.14 (1H, t, J = 7.2 Hz, C$\underline{H}$), 3.62 (3H, s, O–C$\underline{H}_3$), 3.07 (1H, dd, J = 14.3, 6.3 Hz, C$\underline{H}_2$), 2.96 (1H, dd, J = 14.1, 6.8 Hz, C$\underline{H}_2$), 1.48–1.38 (1H, m, C$\underline{H}_2$), 1.12–0.91 (1H, m, C$\underline{H}_2$), 0.91–0.83 (6H, m, C$\underline{H}_3$). ^{13}C NMR (101 MHz, DMSO–

d$_6$) *δ*: 171.2, 168.2, 134.8, 129.5, 128.5, 127.1, 56.5, 53.1, 51.8, 37.0, 36.5, 24.7, 15.3, 11.1. HRMS [M+H]$^+$ calculated for $[C_{16}H_{26}N_2O_3]^+$: 293.1860, found: 293.1867

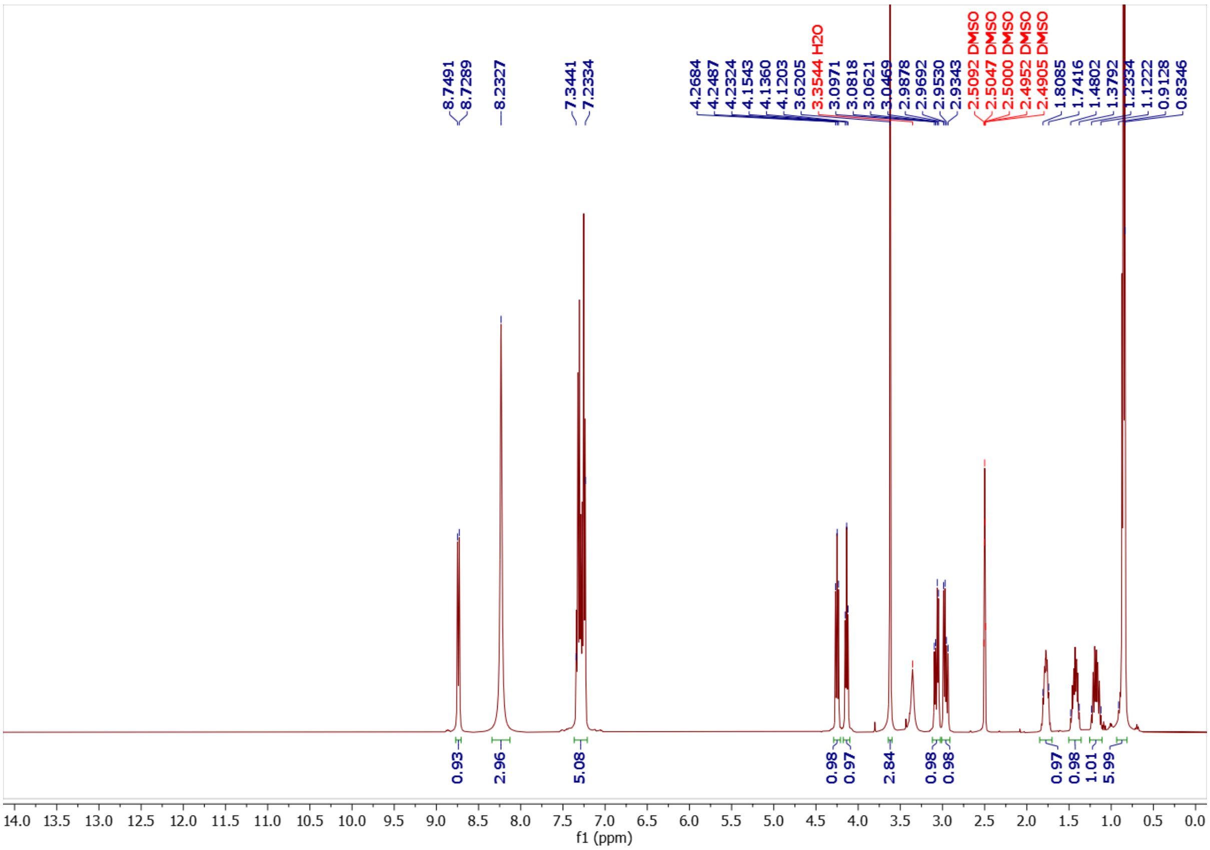

Supplementary Figure 25. ^{1}H NMR spectrum of compound **10** in DMSO–*d*$_6$.

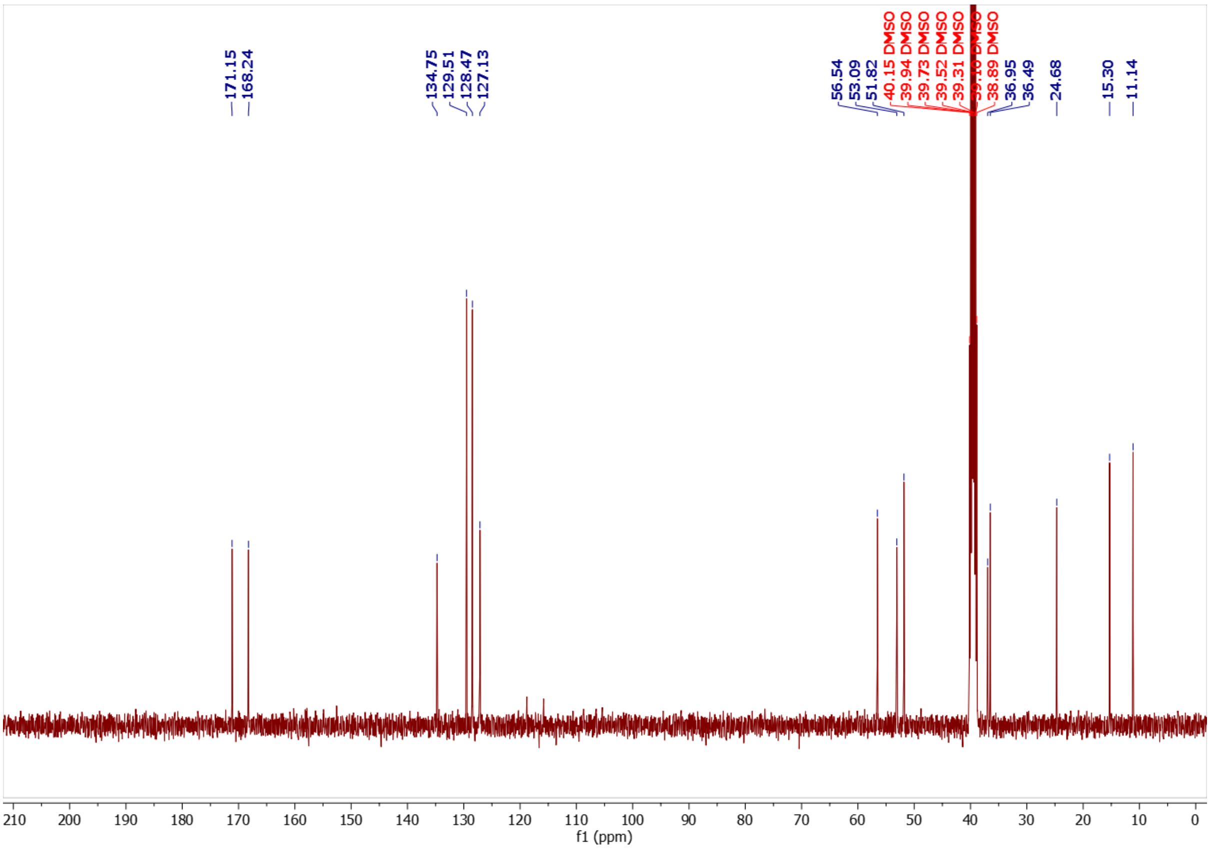

Supplementary Figure 26. ^{13}C NMR spectrum of compound **10** in DMSO–d_6.

Synthesis of methyl [2-(4-{phenyldiazenyl}phenoxy)acetyl])-L-phenylalanyl-L-leucinate (Compound **11**):

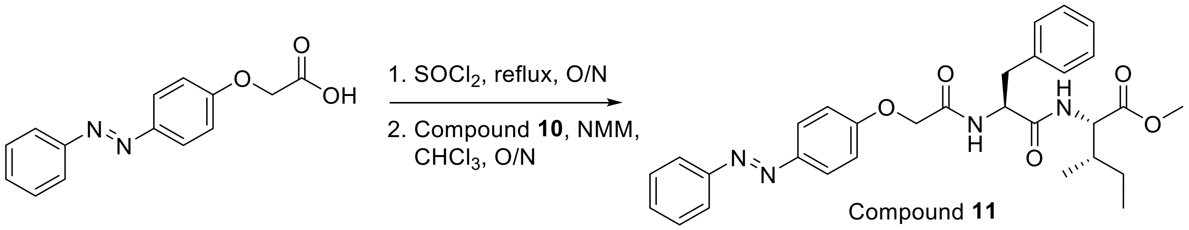

Compound **3** (1.256 g, 4.92 mmol) was refluxed in thionyl chloride (13.0 mL, 178 mmol) for 3 h under nitrogen atmosphere. The excess thionyl chloride was removed via distillation under vacuum to yield a red solid, which was dissolved in chloroform (40 mL) and cooled in an ice bath. A separate solution of compound **10** (2.0 g 4.92 mmol) and *N*-methyl morpholine (3.4 mL, 22.1 mmol) in chloroform (50 mL) was added and the reaction mixture was stirred for 30 min. *N*-methyl morpholine (3.40 mL, 22.1 mmol) was added and the resulting mixture was stirred overnight at room temperature. The resulting solution was washed with water (200 mL × 2) and brine (200 mL). The separated organic layer was dried over anhydrous magnesium sulfate, which was then removed by filtration, and the solvent removed in vacuo to give the product as a bright yellow solid. Yield: 1.74 g, 67%. ^{1}H NMR (400 MHz, DMSO–d_6) δ: 8.40 (1H, d, J = 8.0 Hz, N<u>H</u>), 8.25 (1H, d, J = 8.5 Hz, N<u>H</u>), 7.87–7.81 (4H, m, $\underline{H}_{Ar}$), 7.61–7.51 (3H, m, $\underline{H}_{Ar}$), 7.27–7.18 (5H, m, $\underline{H}_{Ar}$), 7.00 (2H, dd, J = 9.0, 2.0 Hz, $\underline{H}_{Ar}$), 4.76 (1H, td, J = 9.3, 4.4 Hz, NH–C<u>H</u>), 4.58 (2H, s, O–C$\underline{H}_2$), 4.23 (1H, dd, J = 8.0, 6.5 Hz, NH–C<u>H</u>), 3.64 (3H, s, O–C$\underline{H}_3$), 3.03 (1H, dd, J = 13.8, 4.4 Hz, C$\underline{H}_2$), 2.86 (1H, dd, J = 13.8, 9.7 Hz, C$\underline{H}_2$) 1.83–1.74 (1H, m, NH–CH–C<u>H</u>), 1.46–1.36 (1H, m,

C$\underline{H}_2$–CH_3), 1.24–1.13 (1H, m, C$\underline{H}_2$–CH_3), 0.86–0.82 (6H, m, C$\underline{H}_3$). ^{13}C NMR (101 MHz, DMSO–d_6) δ: 171.8, 171.2, 166.9, 160.4, 152.0, 146.4, 137.5, 130.9, 129.4, 129.3, 128.0, 126.3, 124.4, 122.3, 115.2, 66.7, 56.5, 53.1, 51.7, 37.5, 36.3, 24.8, 15.4, 11.1. HRMS [M+Na]$^+$ calculated for [$C_{30}H_{34}N_4NaO_5$]$^+$: 553.2421, found: 553.2422.

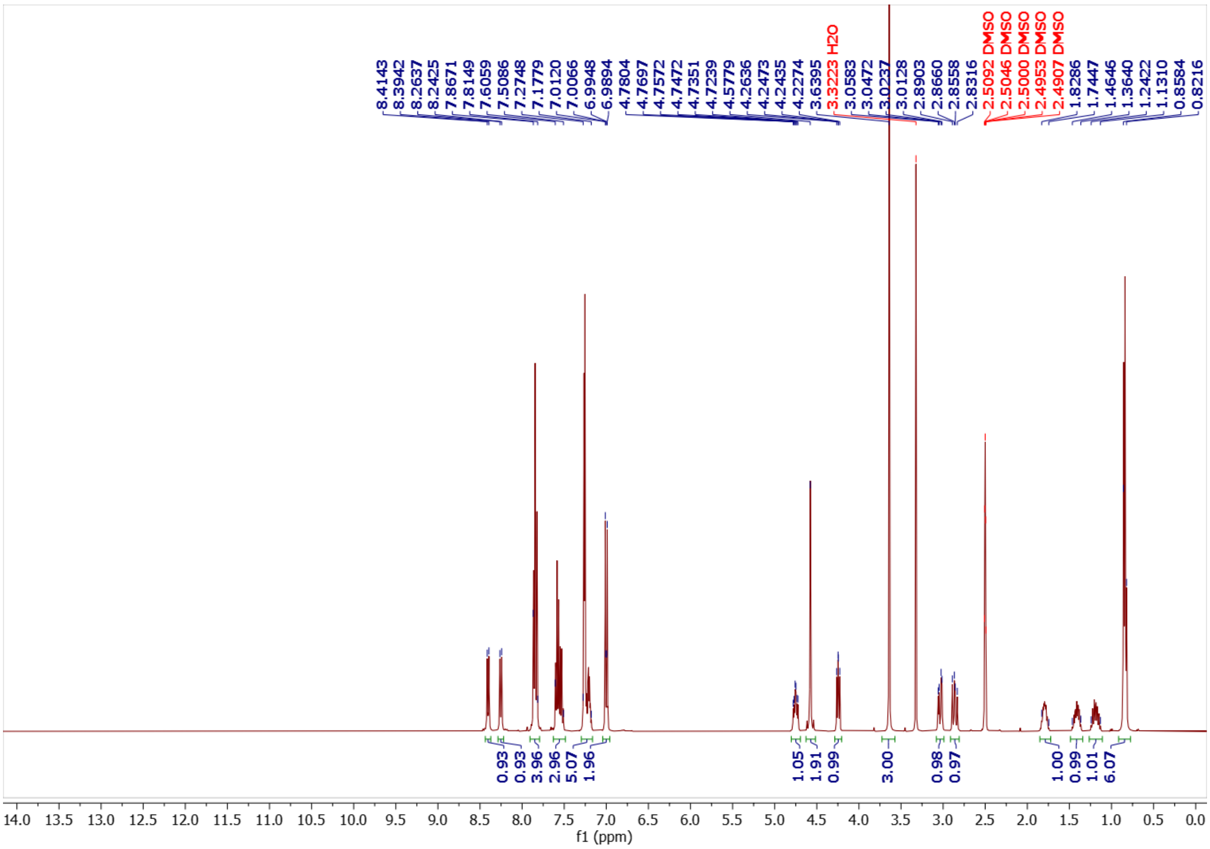

Supplementary Figure 27. ^{1}H NMR spectrum of compound **11** in DMSO–d_6.

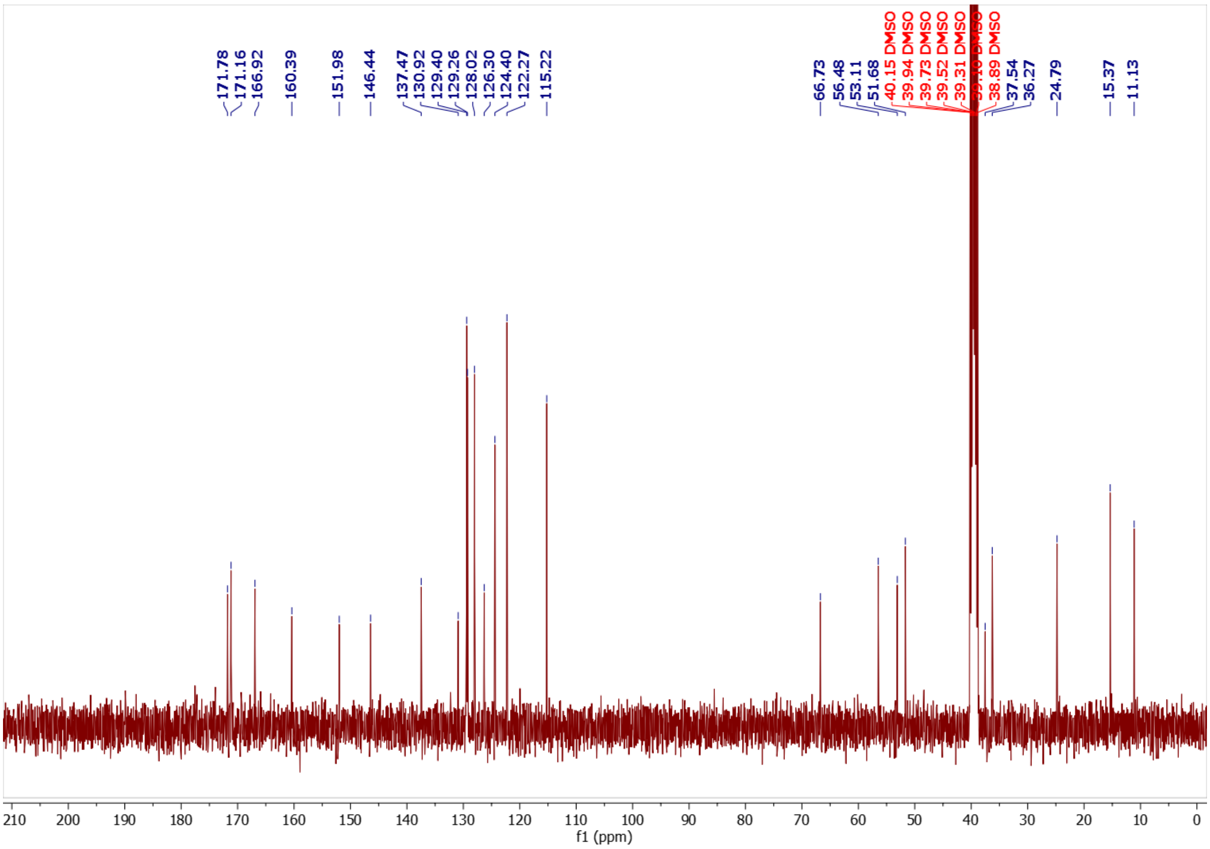

Supplementary Figure 28. ^{13}C NMR spectrum of compound **11** in DMSO–d_6.

Synthesis of [2-(4-{phenyldiazenyl}phenoxy)acetyl]-L-phenylalanyl-L-isoleucine (**Azo-FI**):

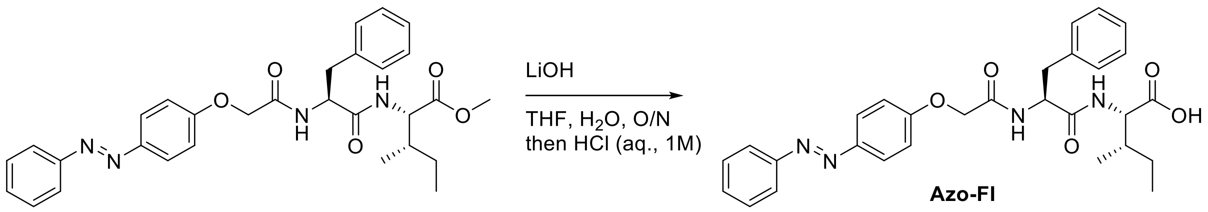

To a mixture of compound **11** (1.70 g, 3.20 mmol) and lithium hydroxide (0.31 g, 12.8 mmol) in tetrahydrofuran (30 mL) and water (30 mL) was stirred overnight. The resulting clear solution was poured into aqueous hydrochloric acid (1 M, 200 mL) and stirred for 90 min. The resulting precipitate was filtered off, washed with water (300 mL) and stirred in ether overnight, then filtrated and dried in a vacuum oven at 50°C to give the product as an orange solid. Yield: 1.52 g, 92%. ^{1}H NMR (400 MHz, DMSO–d_6) δ: 12.68 (1H, bs, COOH), 8.25 (2H, d, J = 8.5 Hz, NH), 7.87–7.81 (4H, m, H_{Ar}), 7.60–7.51 (3H, m, H_{Ar}), 7.29–7.18 (5H, m, H_{Ar}), 6.97 (2H, dd, J = 9.0, 2.0 Hz, H_{Ar}), 4.77 (1H, td, J = 13.2, 6.5 Hz, NH–CH), 4.58 (2H, s, O–CH_2), 4.23 (1H, dd, J = 8.5, 6.0 Hz, NH–CH), 2.89 (1H, dd, J = 13.5, 4.0 Hz, CH_2), 2.69 (1H, dd, J = 13.5, 10.0 Hz, CH_2), 1.86–1.73 (1H, m, NH–CH–CH), 1.48–1.38 (1H, m, CH_2–CH_3), 1.25–1.14 (1H, m, CH_2–CH_3), 0.92–0.83 (6H, m, CH_3). ^{13}C NMR (101 MHz, DMSO–d_6) δ: 172.8, 171.0, 166.9, 160.4, 152.0, 146.5, 137.6, 130.9, 129.4, 129.3, 128.0, 126.3, 124.4, 122.3, 115.2, 66.8, 56.4, 53.2, 37.5, 36.4, 24.7, 15.5, 11.3. HRMS $[M+H]^+$ calculated for $[C_{29}H_{33}N_4O_5]^+$: 517.2445, found: 517.2460.

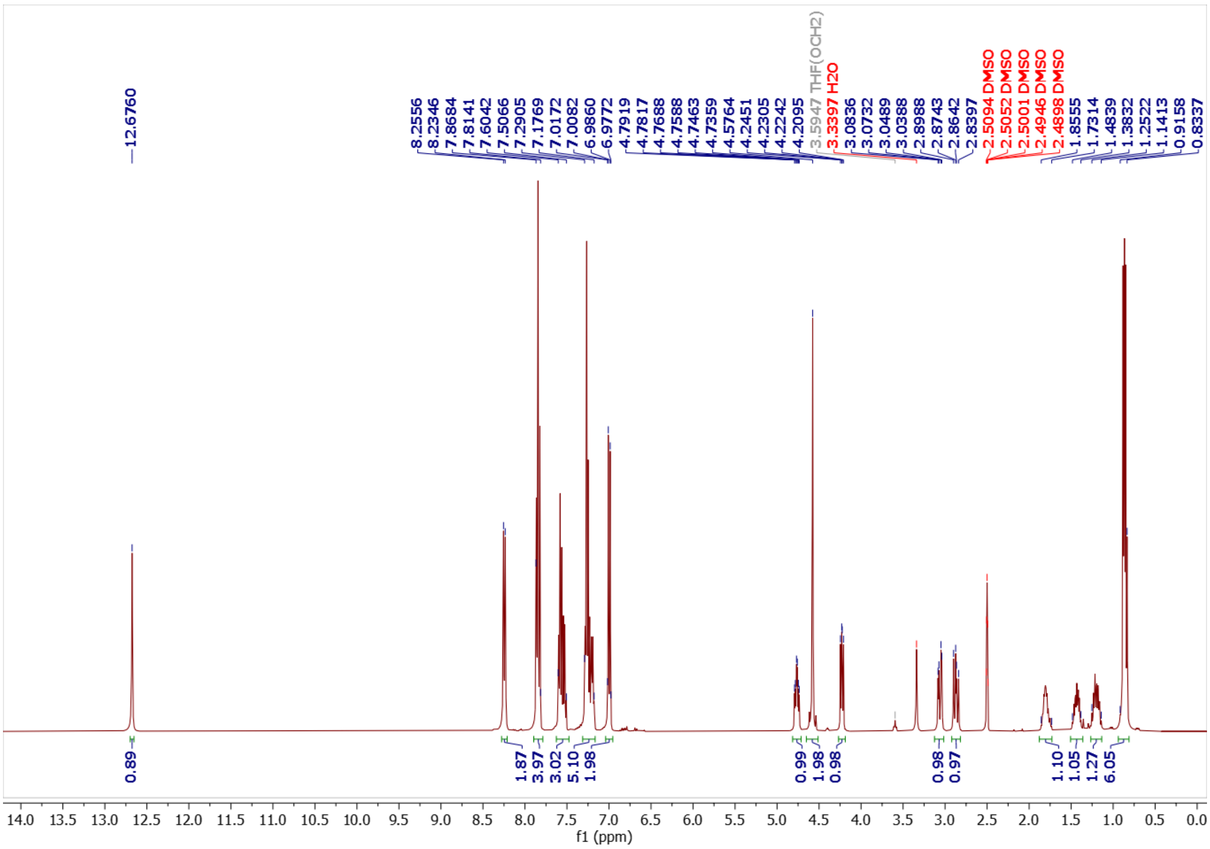

Supplementary Figure 29. ^{1}H NMR spectrum of Azo-FI in DMSO–d_6.

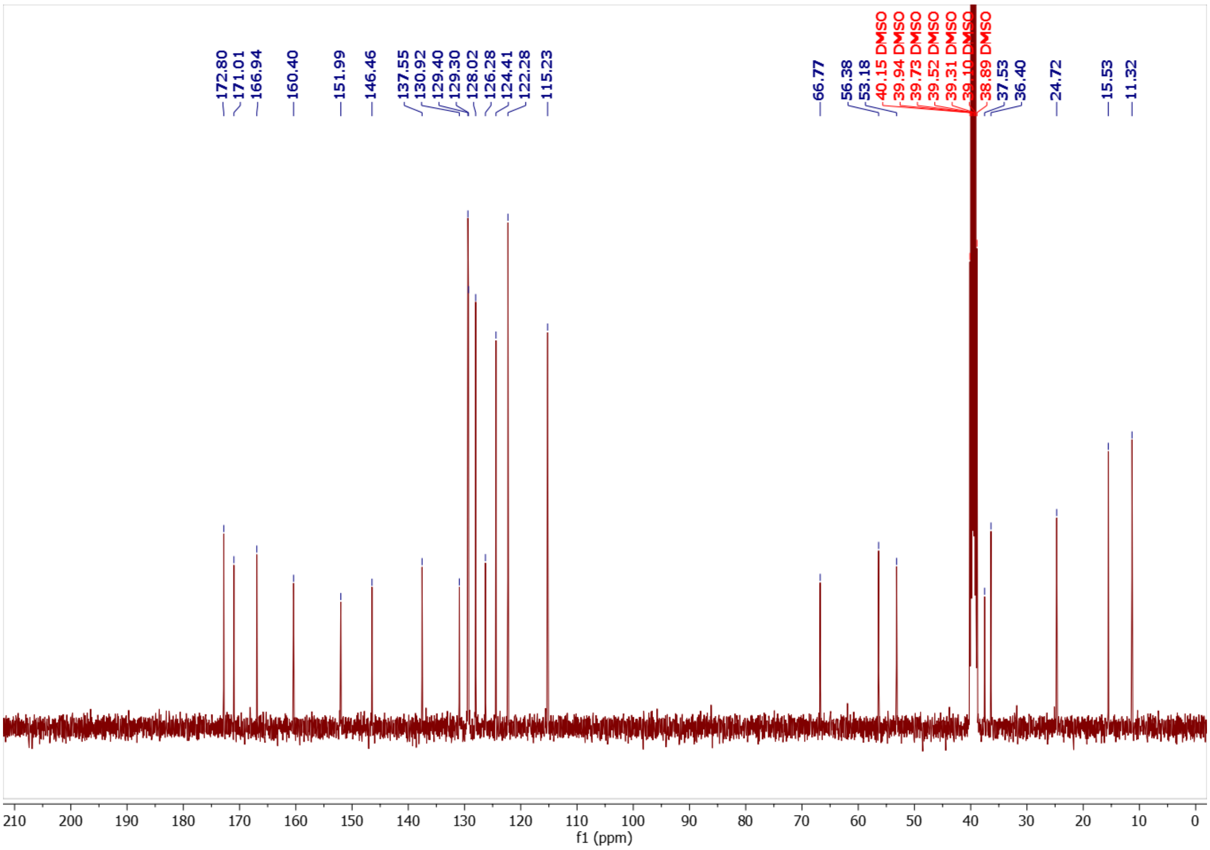

Supplementary Figure 30. ^{13}C NMR spectrum of Azo-FI in DMSO–d_6.

Supplementary Data

Polarised microscopy and WAXS data

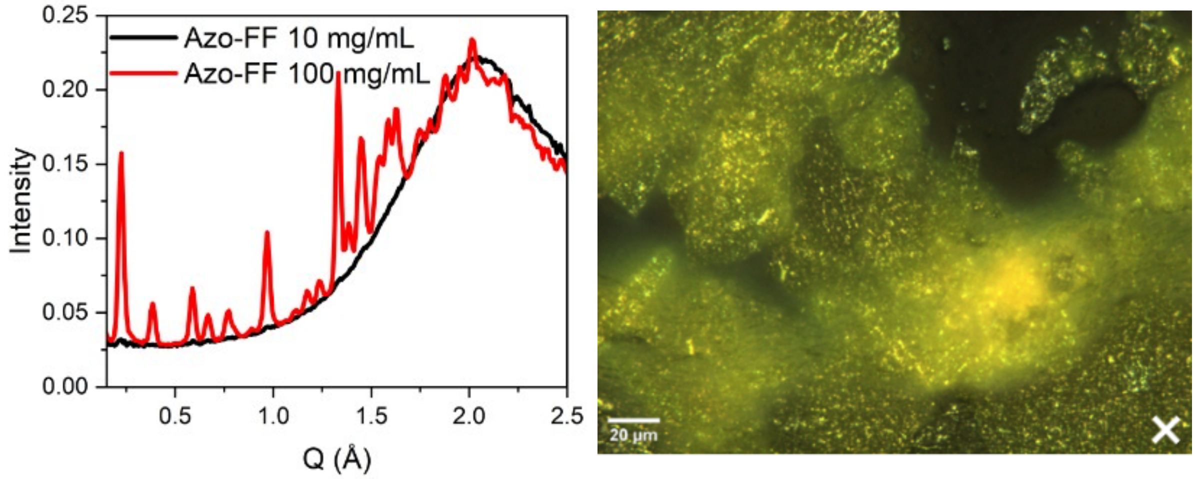

Supplementary Figure 31. (Left) WAXS data for Azo-FF at a concentration of 10 mg/mL and 100 mg/mL. Whilst weak, the same peaks are present in the 10 mg/mL sample as in the 100 mg/mL. (Right) polarised microscope image of the sample at 100 mg/mL. The arrows show the direction of the polarisers.

UV-vis Spectra

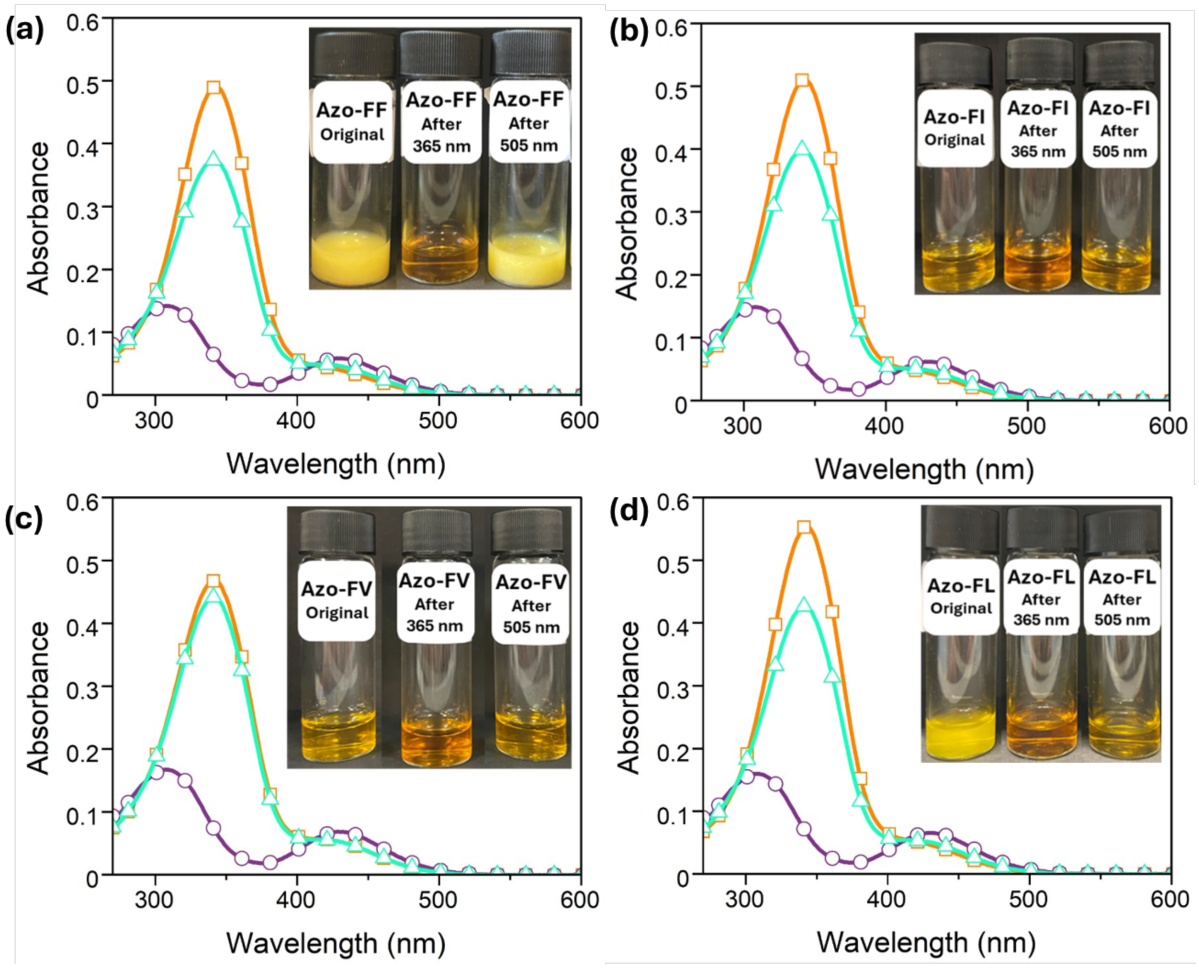

Supplementary Figure 32. UV–vis spectra for solutions of Azo-FF (a), Azo-FI (b), Azo-FV (c) and Azo-FL(d) as the initial *trans*-isomer (orange), after isomerization to the *cis*-isomer with 365 nm LED irradiation (purple) and after reversal back to the *trans*-isomer from 505 nm LED irradiation (pale blue). The inset shows photographs of the solutions. Due to high absorbance, UV-Vis spectra were recorded using thin cuvettes (optical pathlength: 0.1 ± 0.005 mm), thereby improving spectral accuracy and removing issues with scattering. All isomerizations were carried out by placing two LEDs 5 cm away from the sample and irradiating for 10 minutes. All data were collected at pH 10.5 at a concentration of 10 mg/mL.

Reversibility experiments

Change cycles were carried out switching irradiation with 365 nm or 505 nm LEDs. We placed two LEDs 5 cm away from the sample for alternate radiation for 10 minutes. This was repeated three times in total, and changes in the peak intensity at 340 nm were recorded.

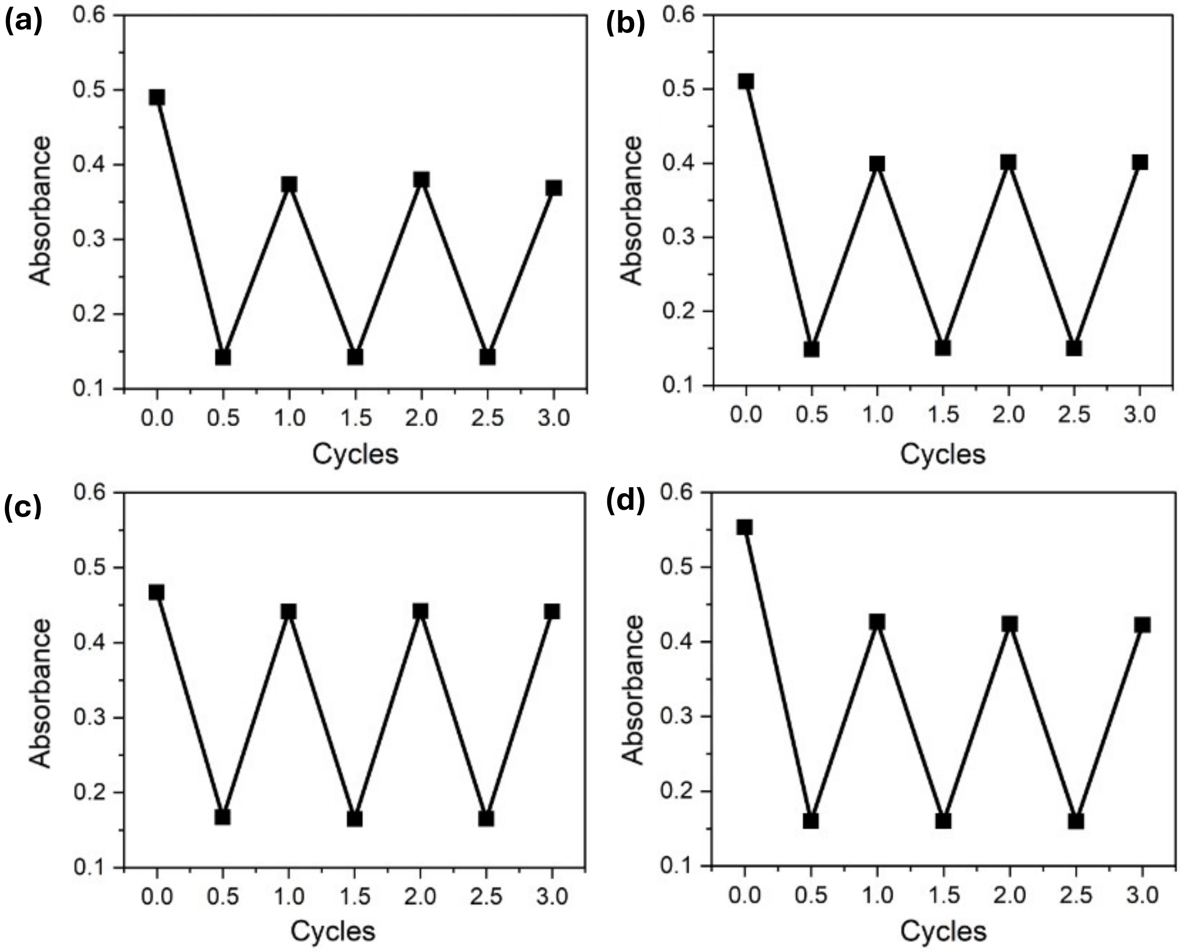

Supplementary Figure 33. Change in absorbance at 340 nm over multiple *trans*-to-*cis*-to-*trans* isomerizations for, Azo-FF (a), Azo-FI (b), Azo-FV (c) and Azo-FL (d). All isomerizations were carried out by placing two LEDs 5 cm away from the sample and irradiating for 10 minutes. All data were collected at pH 10.5 at a concentration of 10 mg/mL.

Shear viscosity

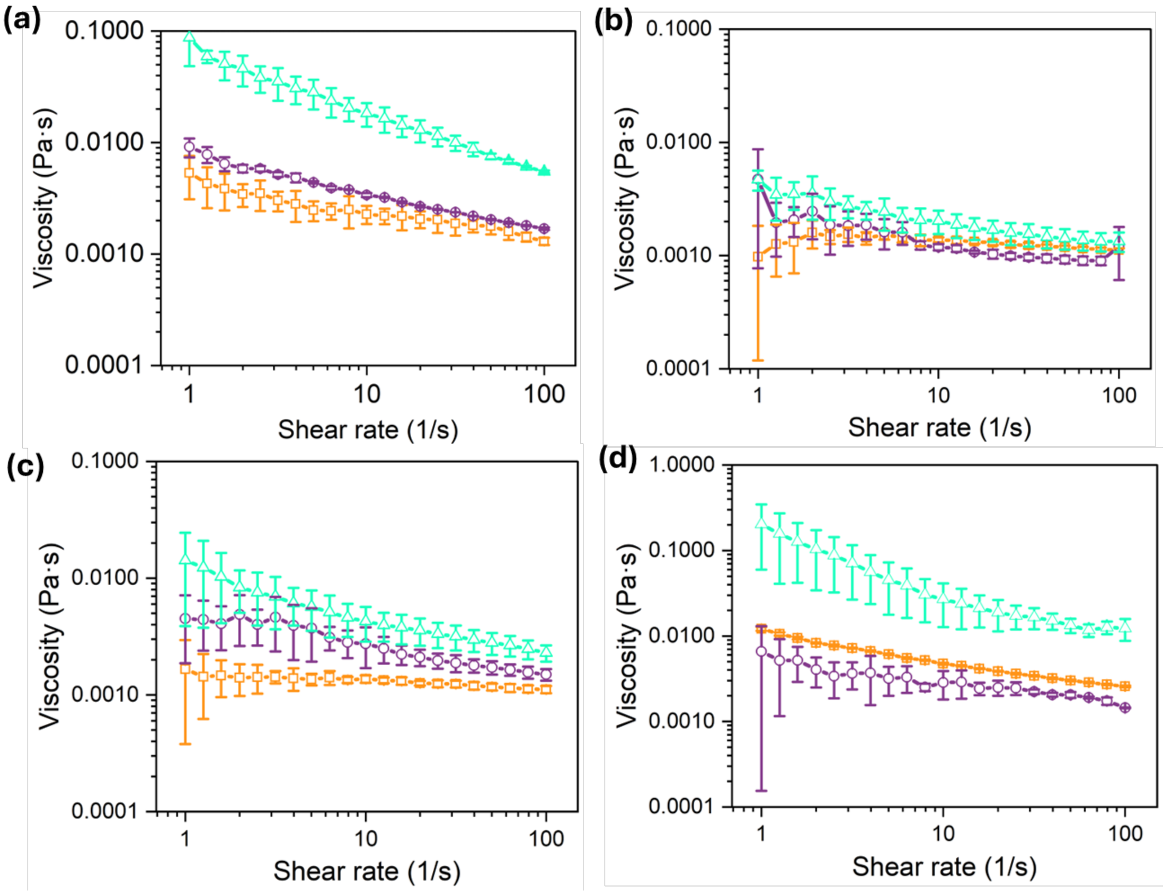

Supplementary Figure 34. Change in viscosity for solutions of Azo-FF (a), Azo-FI (b), Azo-FV (c) and Azo-FL (d) in water at pH 10.5 at a concentration of 10 mg/mL, as the initial *trans*-isomer (orange), after isomerization to the *cis*-isomer after 365 nm irradiation (purple), and after reversal back to the *trans*-isomer after 505 nm irradiation (pale blue). All isomerizations were carried out by placing two LEDs 5 cm away from the sample and irradiating for 10 minutes.

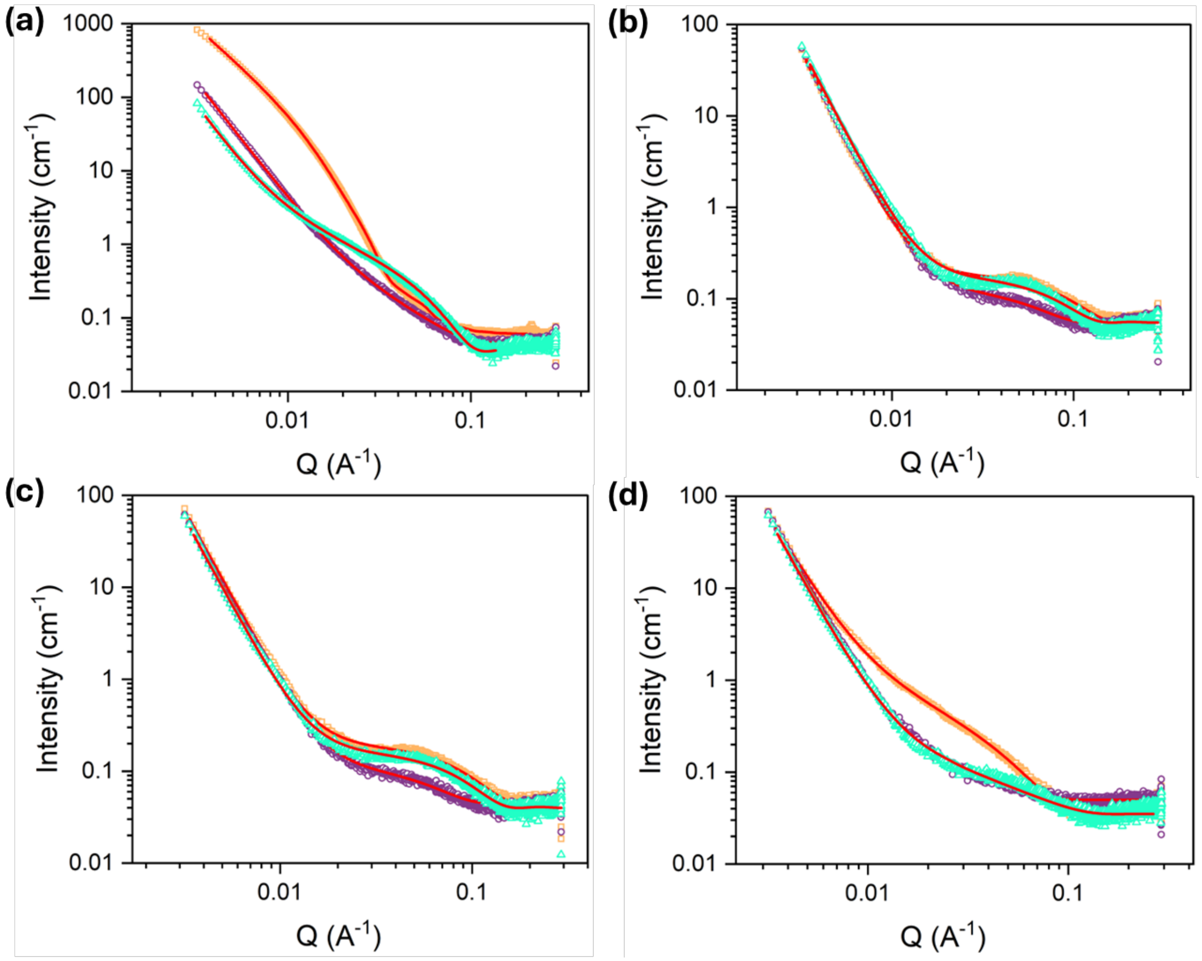

Supplementary Figure 35. SAXS data for solutions of Azo-FF (a), Azo-FI (b), Azo-FV (c) and Azo-FL (d) as the initial *trans*-isomer (orange), after isomerization to the *cis*-isomer after 365 nm irradiation (purple), and after reversal back to the *trans*-isomer after 505 nm irradiation (pale blue). The fits to the data are shown as red lines. The fit parameters are described below in Tables S1-S4. All isomerizations were carried out by placing two LEDs 5 cm away from the sample and irradiating for 10 minutes. All data were collected at pH 10.5 at a concentration of 10 mg/mL.

Supplementary Table 1. Fitting parameters of Azo-FF in water at pH 10.5 at concentration of 10 mg/mL for SAXS data.

Sample	Azo-FF-10 mg pre-gel fresh	Azo-FF-10 mg pre-gel after 365 nm	Azo-FF-10 mg pre gel after 505 nm
Model	Power law + flexible cylinder elliptical	Power law + flexible cylinder	Power law + cylinder
Scale	0.00064728	2.85E-04	0.00108
Scale error	2.12E-06	6.70E-06	2.61E-06
Background cm^{-1}	0.06	0.045	0.035
Background error	-	-	-
Radius / Å	91.081	29.278	31.627
Radius error	0.11245	0.2278	0.053985
Axis radius / Å	2.35	-	29.095
Axis radius error	0.0042963	-	0.10478
Length / Å	3179.1	5000	5048.8
Length error	55.131	-	48.644
Kuhn length/ Å	239.01	290.38	407.36
Kuhn length error	2.2572	117.92	38.512
Power law scale	2.72E-06	1.25E-06	1.11E-07
Power law scale error	4.06E-08	1.56E-07	3.90E-09
Power law	3.3227	3.2414	3.5288
Power law error	0.0035592	2.10E-02	0.006581
Sld	13.902	13.902	13.902
Sld solvent	9.469	9.469	9.469

Supplementary Table 2. Fitting parameters of Azo-FI in water at pH 10.5 at concentration of 10 mg/mL for SAXS data.

Sample	Azo-FI-10 mg pre-gel fresh	Azo-FI-10 mg pre-gel after 365 nm	Azo-FI-10 mg pre-gel after 505 nm
Model	Power law + sphere	Power law + sphere	Power law + sphere
Scale	0.001099	0.000174	0.000672
Scale error	7.61E-06	3.21E-06	5.92E-06
Background cm^{-1}	0.055	0.055	0.055
Background error	-	-	-
Radius / Å	24.047	35.818	27.713
Radius error	0.075388	0.31475	0.11167
Power law scale	6.66E-09	1.54E-08	1.63E-08
Power law scale error	2.89E-10	6.07E-10	6.32E-10
Power law	3.9525	3.8131	3.8151
Power law error	0.008119	0.007406	0.007346
Sld	14	14	14
Sld solvent	9.469	9.469	9.469

Supplementary Table 3. Fitting parameters of Azo-FV in water at pH 10.5 at concentration of 10 mg/mL for SAXS data.

Sample	Azo-FV-10 mg pre-gel fresh	Azo-FV-10 mg pre-gel after 365 nm	Azo-FV-10 mg pre-gel after 505 nm
Model	Power law + sphere	Power law + sphere	Power law + sphere
Scale	0.001201	0.00016509	0.000873
Scale error	7.10E-06	3.12E-06	6.35E-06
Background cm^{-1}	0.045	0.055	0.04
Background error	-	-	-
Radius / Å	24.36	35.605	25.613
Radius error	0.065695	0.32271	0.085447
Power law scale	1.85E-08	2.37E-08	1.46E-08
Power law scale error	5.18E-10	7.85E-10	5.36E-10
Power law	3.8332	3.7567	3.8379
Power law error	0.005277	0.006232	0.006955
Sld	13.971	13.971	13.971
Sld solvent	9.469	9.469	9.469

Supplementary Table 4. Fitting parameters of Azo-FL in water at pH 10.5 at a concentration of 10 mg/mL for SAXS data.

Sample	Azo-FL-10 mg pre-gel fresh	Azo-FL-10 mg pre-gel after 365 nm	Azo-FL-10 mg pre-gel after 505 nm
Model	Power law + cylinder	Power law + cylinder	Power law + cylinder
Scale	0.000411	9.99E-05	0.000246
Scale error	1.93E-06	2.58E-06	5.24E-06
Background cm^{-1}	0.045	0.05	0.035
Background error	-	-	-
Radius / Å	35.31	30.513	22.142
Radius error	0.11688	0.52159	0.29387
Length / Å	4667.4	4852.8	445.51
Length error	120.54	498.29	54.075
Power law scale	3.00E-08	8.96E-09	7.78E-09
Power law scale error	1.26E-09	3.78E-10	4.41E-10
Power law	3.7278	3.9473	3.9569
Power law error	0.00778	0.007794	0.010325
Sld	14	14	14
Sld solvent	9.469	9.469	9.469

Supplementary Table 5. Fitting parameters of Azo-FV *trans*-gel, *cis*-gel, and *re-trans*-gel for SAXS data.

Sample	Azo-FV *trans*-gel	Azo-FV *cis*-gel	Azo-FV *re-trans*-gel
Model	Power law + cylinder elliptical	Power law + cylinder elliptical	Power law + cylinder elliptical
Scale	0.0023509	0.0015788	0.0016032
Scale error	4.62E-06	6.10E-06	3.69E-06
Background cm^{-1}	0.05	0.052	0.047
Background error	-	-	-
Radius / Å	26.428	32.459	27.705
Radius error	0.081457	0.071963	0.10102
Axis radius / Å	1.9184	3.1719	2.0497
Axis radius error	0.010542	0.014075	0.012919
Length / Å	5000	5000	5000
Length error	-	-	-
Power law scale	5.34E-07	2.12E-06	9.62E-08
Power law scale error	3.29E-08	1.69E-07	5.80E-09
Power law	3.1954	3.0263	3.5135
Power law error	0.011389	0.014227	0.011077
Sld	13.7	13.7	13.7
Sld solvent	9.469	9.469	9.469

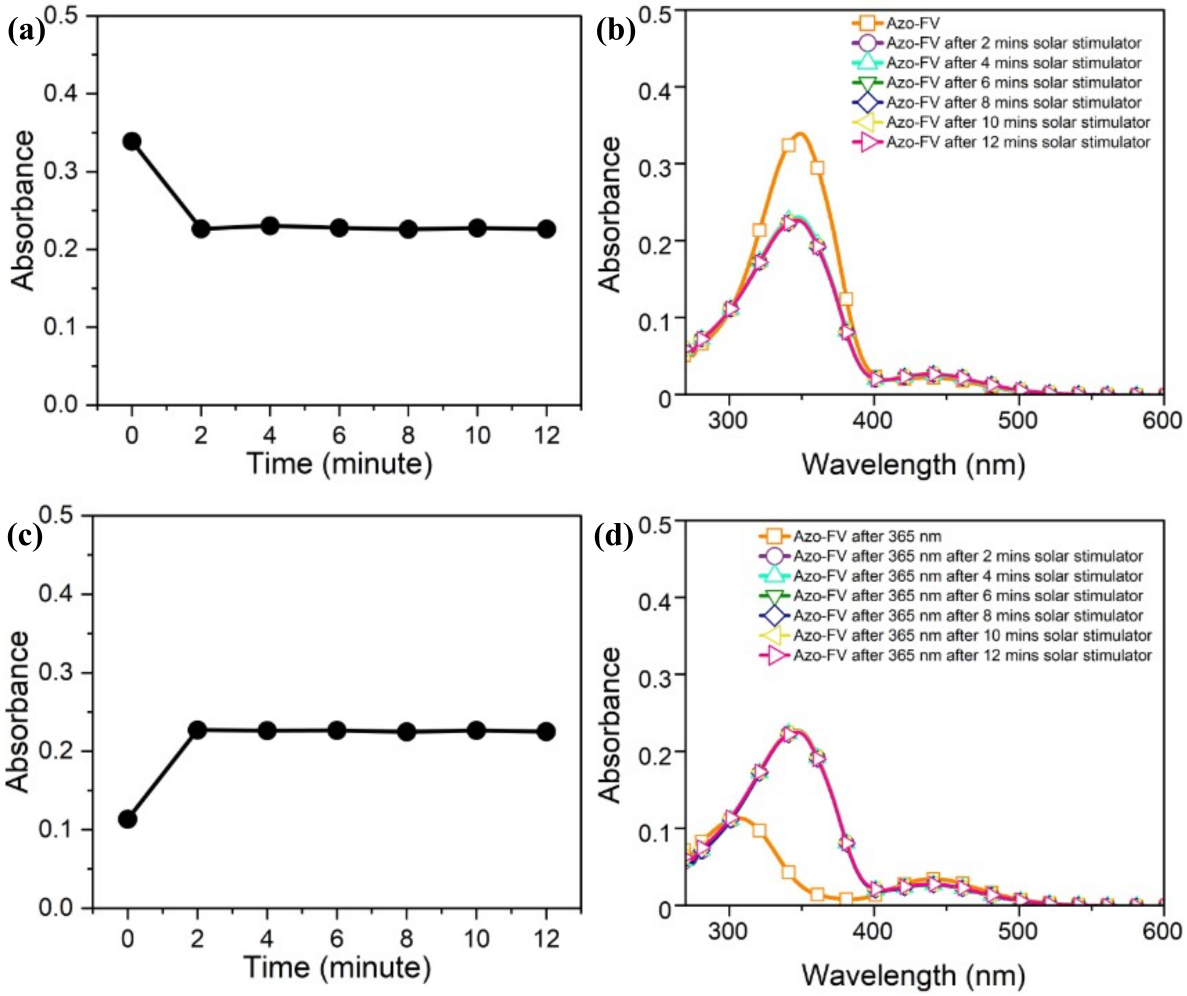

Supplementary Figure 36. UV–vis spectra for solutions of Azo-FV at a concentration of 3 mg/mL in DMSO irradiated using a solar simulator. (a) shows the change in absorbance at the maximum value between 270 and 400 nm with irradiation time and (b) shows the raw data after irradiation for specific times for the solution initially as the *trans*-isomer. (c) shows the change in absorbance at the maximum value between 270 and 400 nm with irradiation time and (b) shows the raw data after irradiation for specific times for the solution initially as the *cis*-isomer.

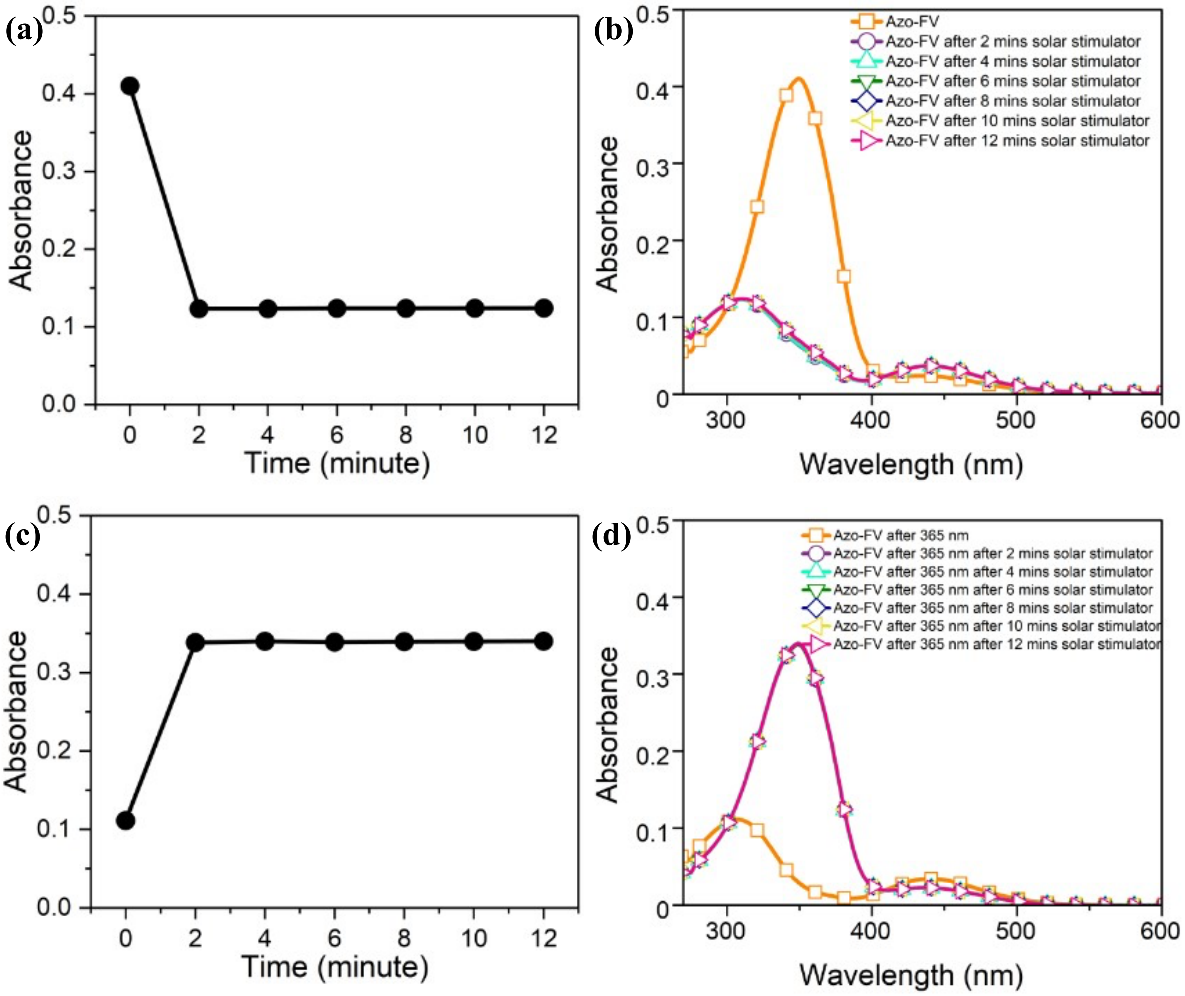

Supplementary Figure 37. UV–vis spectra for solutions of Azo-FV at a concentration of 3 mg/mL in DMSO irradiated using a solar simulator. (a) shows the change in absorbance at the maximum value between 270 and 400 nm with irradiation time and (b) shows the raw data after irradiation for specific times for the solution initially as the *trans*-isomer. Irradiation was carried out with a THOR LABS 390 nm short-pass filter lens above the sample. (c) shows the change in absorbance at the maximum value between 270 and 400 nm with irradiation time and (b) shows the raw data after irradiation for specific times for the solution initially as the *cis*-isomer. Irradiation was carried out with a THOR LABS 496 nm long-pass filter lens above the sample.

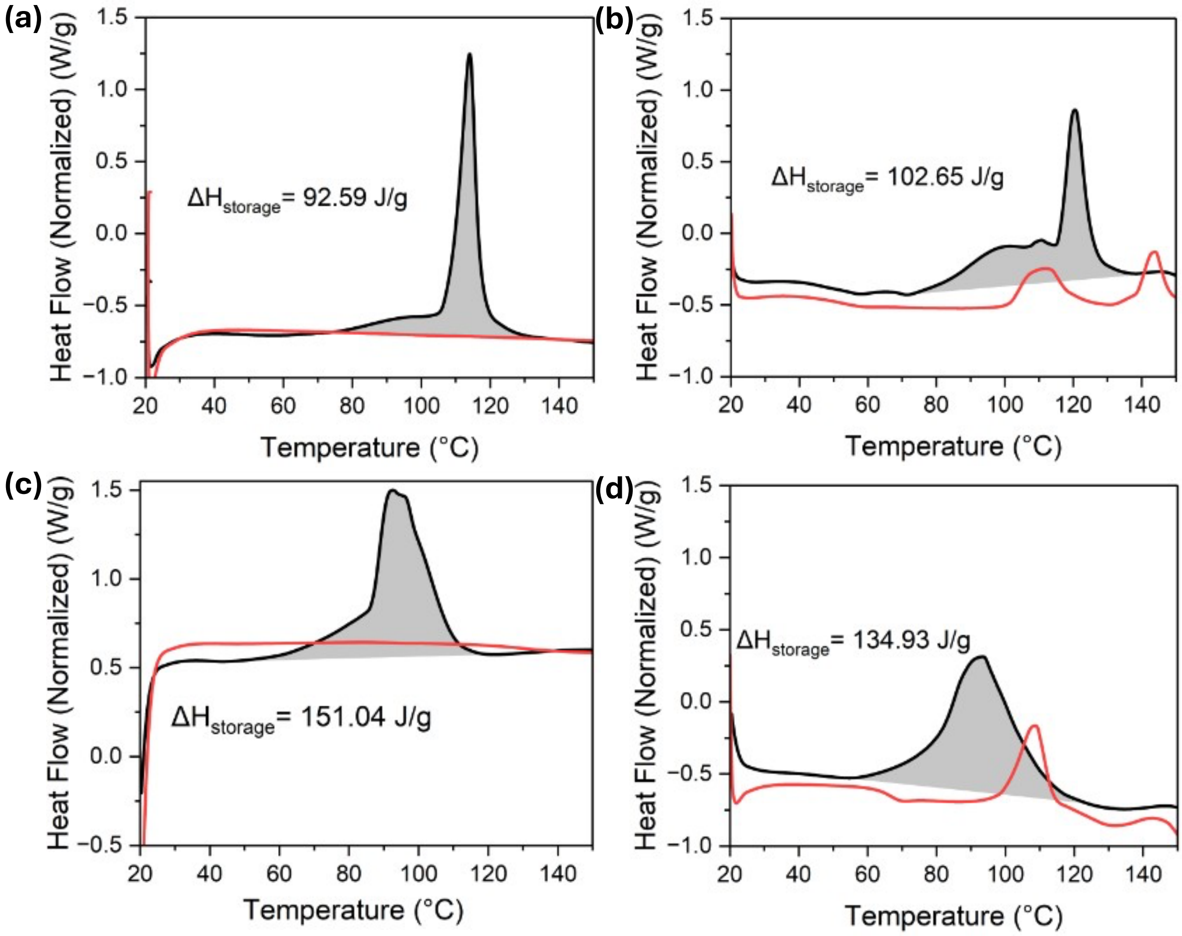

Supplementary Figure 38. DSC thermograms of Azo-FF (a), Azo-FI (b), Azo-FV (c) and Azo-FL(d). The first heating cycle (black line) is accompanied by the typical exothermic peak associated to the back isomerization. The second heating cycle, shown as a red line, demonstrates that due to specific heat capacity difference of each sample, at a heating rate of 10 °C min^{-1}, samples Azo-FI and Azo-FL are unable to fully release their stored energy as latent heat based on the NMR data after measurement (Figure S36). Note the data in (c) are the same as in Figure 3a.

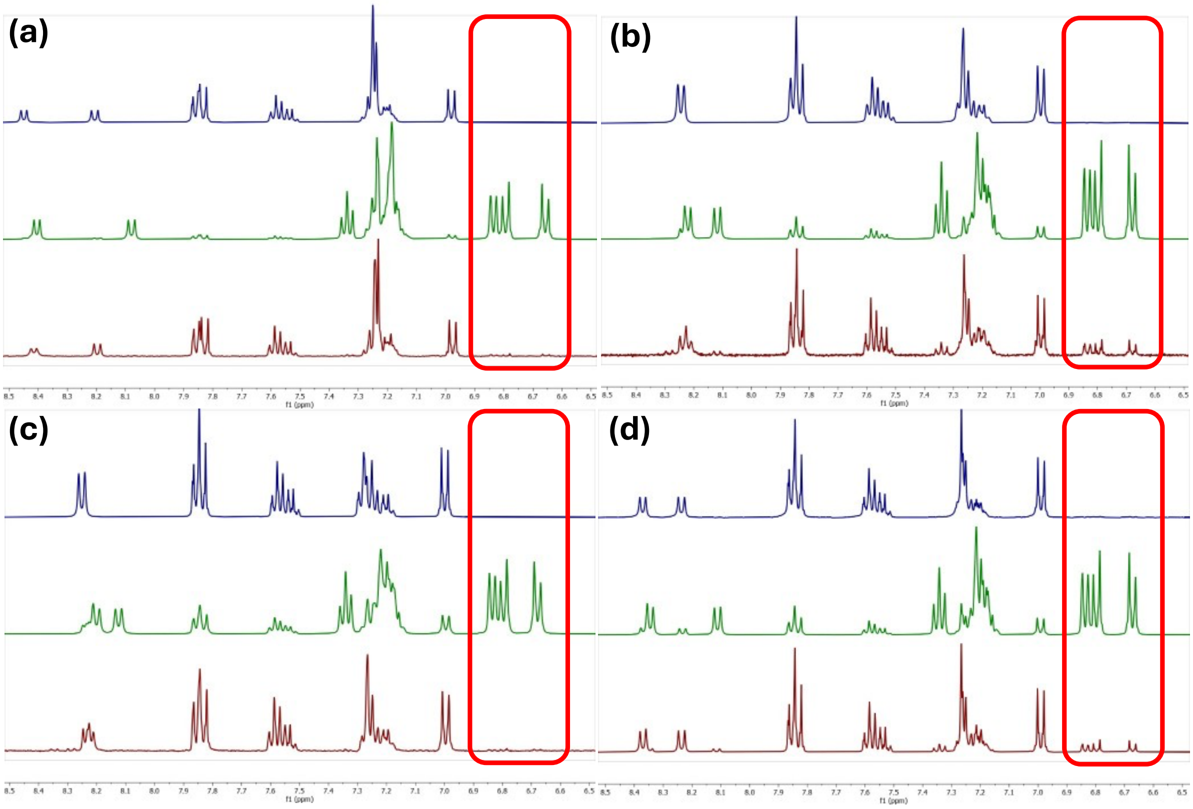

Supplementary Figure 39. ^{1}H NMR spectrum of Azo-FF (a), Azo-FI (b), Azo-FV (c) and Azo-FL (d) between *δ*: 8.5–6.5 in DMSO–d_6. as the initial *trans*-isomer (top), isomerization to the *cis*-isomer with 365 nm LED irradiation before DSC (middle) and after DSC (bottom). The red box indicates the characteristic peaks of the *cis*-isomers.

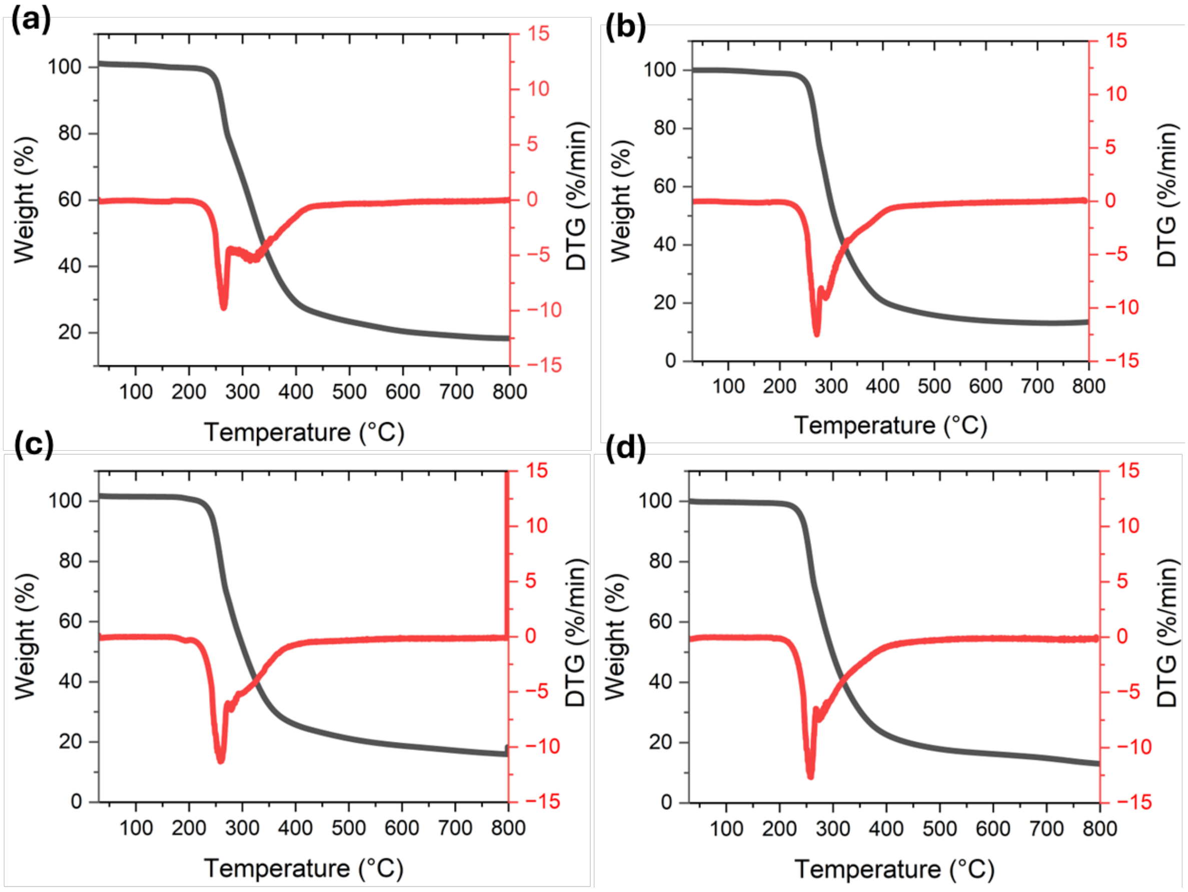

Supplementary Figure 40. Thermogravimetric analysis (TGA, black) and derivative thermogravimetry (DTG, red) curves of Azo-FF (a), Azo-FI (b), Azo-FV (c) and Azo-FL (d) at a heating rate of 10°C/min.

Supplementary Note 1 - Thermal kinetics[S2]

Temperature control was achieved using an Agilent Technologies GARY single cell Peltier accessory temperature controller with ethylene glycol cooling. The UV–vis absorbance spectrum in the native state was first taken at 25°C. The sample was irradiated for 10 minutes under UV light (LedEngin Inc 365 nm, Ultraviolet LED, 700 mA). Spectra were then taken every 30 minutes for 34 measurements at temperatures of 30, 40 and 50°C.

The thermal back–conversion of the photoisomer to the parent molecule can be described by a pseudo first–order kinetic equation:

$$[A(\lambda)](t) = [A_0(\lambda)]e^{-kt} \quad \text{Eq. S1}$$

where $[A(\lambda)](t)$, $[A_0(\lambda)]$ are the actual and initial concentrations, respectively; k is the reaction rate constant at a specific temperature in s^{-1} and t is the reaction time in s.

The reaction rate constant depends on the thermal activation barrier of the back conversion. This energy barrier can be quantitatively described using the Eyring equation.

$$k = \frac{k_B T}{h} e^{-\frac{\Delta G^{\ddagger}}{RT}} \quad \text{Eq. S2}$$

Where k_B is Boltzmann's constant in J K^{-1}, h is Planck's constant in J s, and $\Delta G^{\ddagger}$refers to the Gibbs free energy in J mol^{-1}. In addition, the Gibbs energy can be written in relation to enthalpy and entropy:

$$\Delta G^{\ddagger} = \Delta H^{\ddagger}_{therm} - T\Delta S^{\ddagger} \quad \text{Eq. S3}$$

Where $\Delta H^{\ddagger}_{therm}$ represents the activation enthalpy of the thermal back–conversion and $\Delta S^{\ddagger}$ is the entropy. Consequently, equation S3 can also be expressed as:

$$ln\frac{k}{T} = ln\frac{k_B}{h} - \frac{\Delta H^{\ddagger}_{therm}}{RT} + \frac{\Delta S^{\ddagger}}{R} \quad \text{Eq. S4}$$

At a specific temperature, $ln\frac{k}{T}$ is a linear equation dependent on $\frac{1}{T}$, and, therefore, k at any given temperature can be determined.

The half–life of the photoisomer at a specific temperature corresponds to a moment where $[A(\lambda)]$ equals $[A_0(\lambda)]/2$. From equation S1, it can then be written as:

$$t_{1/2} = \frac{\ln(2)}{k} \quad \text{Eq. S5}$$

Supplementary Data

Kinetic studies on converted Azo-FV

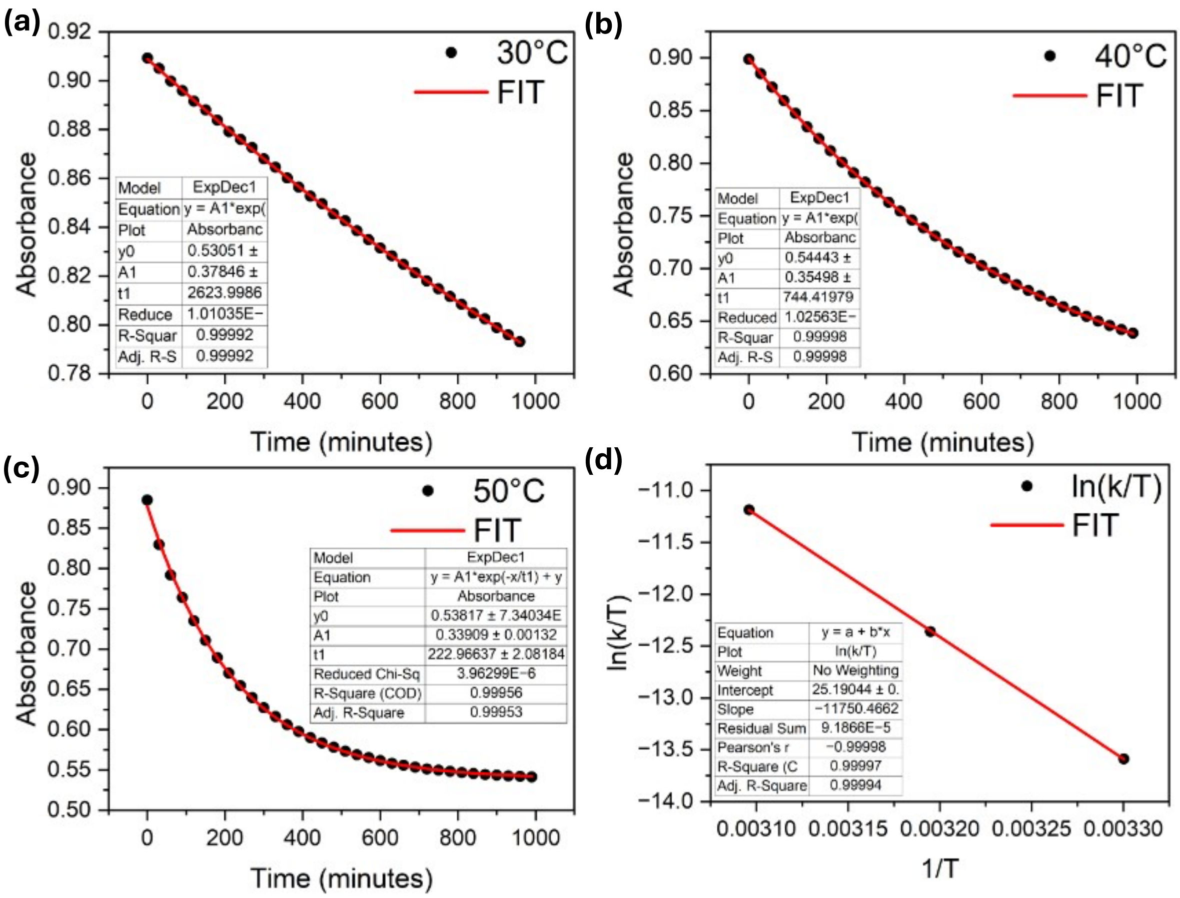

Supplementary Figure 41. Thermal back-conversion kinetics of Azo-FV at a concentration of 3 mg/mL in DMSO. (a) Thermal back-conversion kinetics at 30°C. (b) Thermal back-conversion kinetics at 40°C. (c) Thermal back-conversion kinetics at 50°C. (d) Eyring plot of the Azo-FV at a concentration of 3 mg/mL in DMSO. All isomerizations were carried out by placing two LEDs 5 cm away from the sample and irradiating for 10 minutes. All data were collected at a concentration of 3 mg/mL.

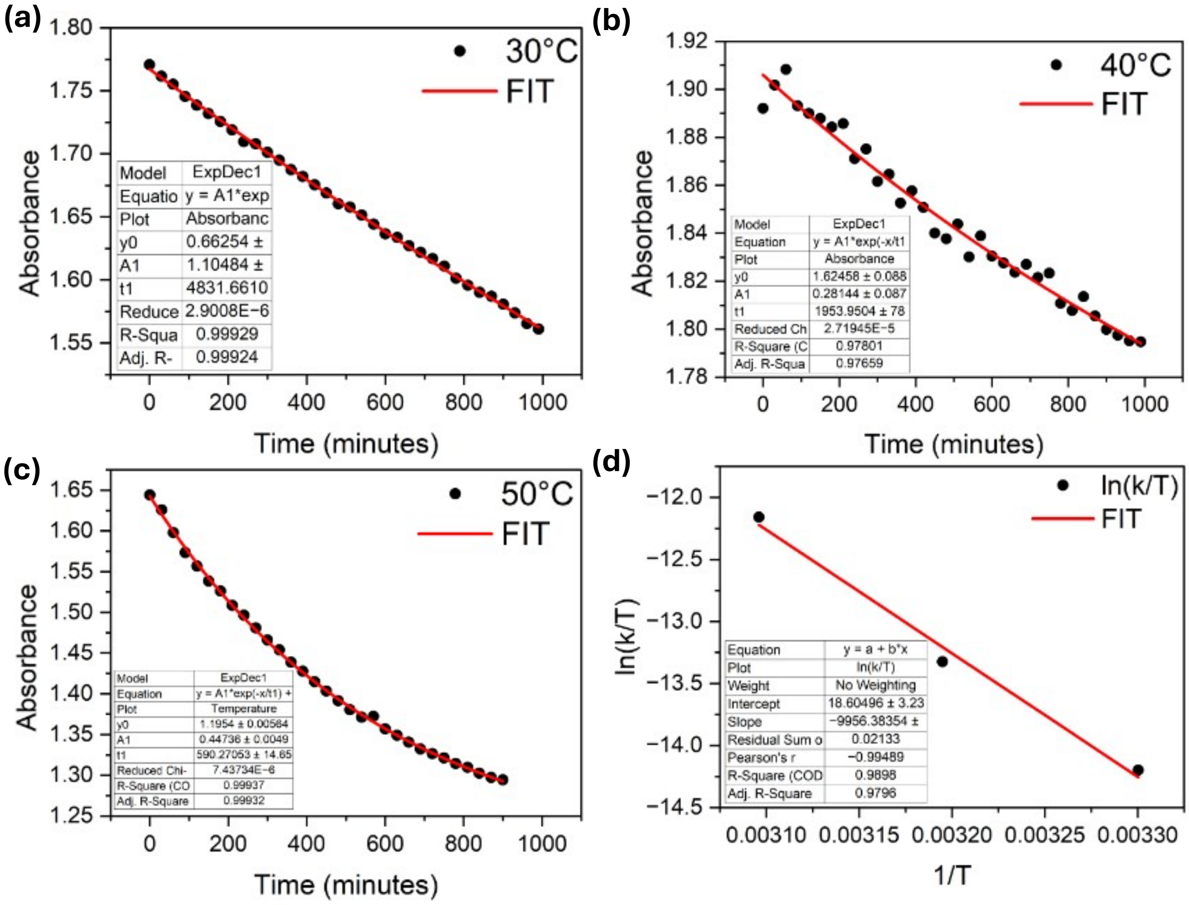

Supplementary Figure 42. Thermal back-conversion kinetics of Azo-FV at a concentration of 3 mg/mL in water at high pH. (a) Thermal back-conversion kinetics at 30°C. (b) Thermal back-conversion kinetics at 40°C. (c) Thermal back-conversion kinetics at 50°C. (d) Eyring plot of the Azo-FV at a concentration of 3 mg/mL in water at high pH.

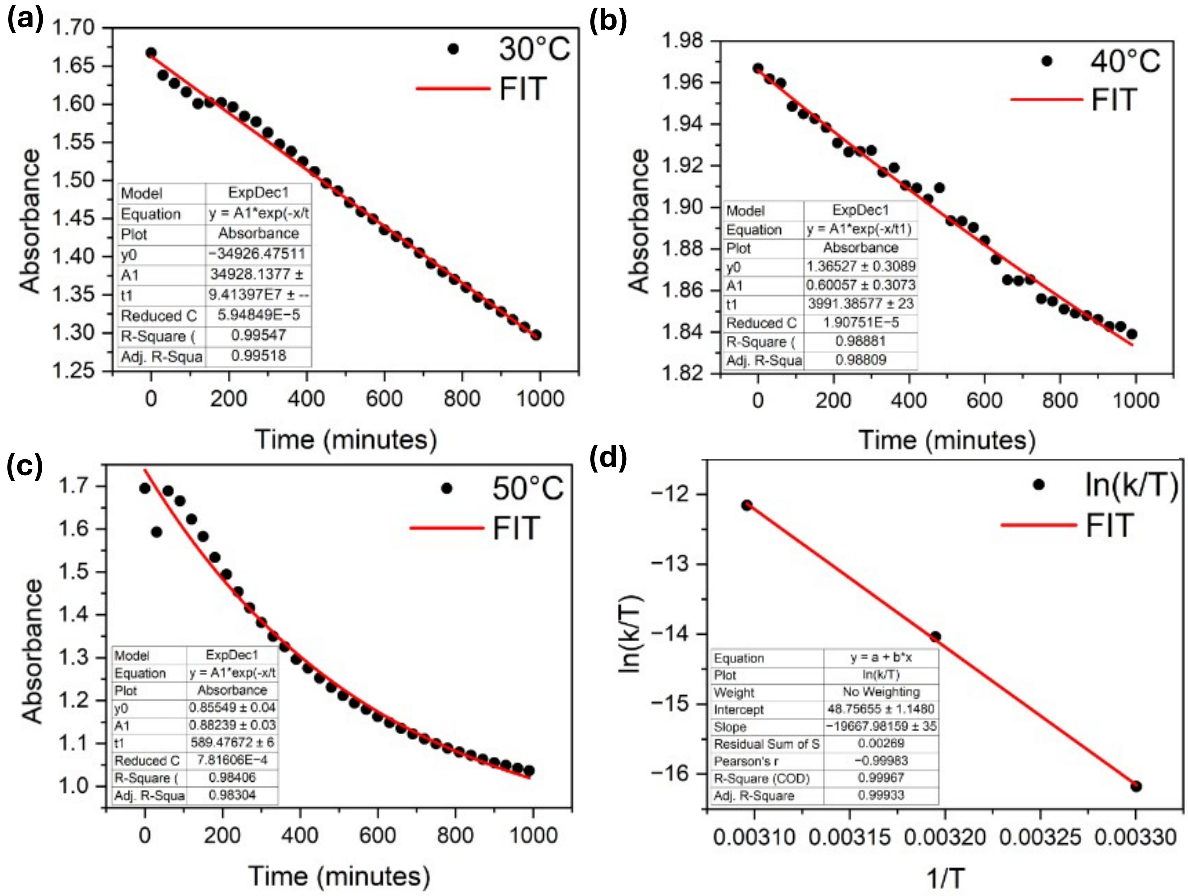

Supplementary Figure 43. Thermal back-conversion kinetics of Azo-FV in the gel state. (a) Thermal back-conversion kinetics at 30°C. (b) Thermal back-conversion kinetics at 40°C. (c) Thermal back-conversion kinetics at 50°C. (d) Eyring plot of the Azo-FV at gel state.

Supplementary Note 2 - Calculation of Activation Energies

According to the Exponential decay equation, $y = y_0 + A_1 e^{-\frac{1}{t_1}}$, the time constant t_1 can be obtained by fitting the measured data. Then, it can be deduced that the reaction rate k is equal to the slope ($Y = \ln\ A_1 - \frac{1}{t_1}X$, $k = -\frac{1}{t_1}$).

The Arrhenius equation is expressed as:

$$k = \mathrm{A}e^{-\frac{\mathrm{E_a}}{RT}}$$

where k is the rate constant, A is the pre-exponential (frequency) factor, E_a is the activation energy, R is the universal gas constant (8.314 J·mol^{-1}·K^{-1}), and T is the absolute temperature.

Taking the natural logarithm of both sides:

$$\ln k = \ln A - \frac{E_a}{RT}$$

Rewriting this in a form suitable for linear regression:

$$\ln k = -(\frac{E_a}{R})\frac{1}{T} + \ln A$$

This is in the form of a straight-line equation:

$$slope = \frac{\Delta(\ln k)}{\Delta(1/T)} = -\frac{E_a}{R}$$

Rearranging to solve for activation energy:

$$E_a = -slope * R$$

The activation energy (E_a) of 3 mg/mL Azo-FV in DMSO, pre-gel, and gel states is calculated to be 85.4 KJ·mol^{-1}, 100.3 KJ·mol^{-1} and 166.1 KJ·mol^{-1}.

Supplementary Data

Cross-Polarized Microscopy

Azo-FV solutions at a concentration of 200 mg/mL and pH 10.5 showed micellar aggregations in CPM images.

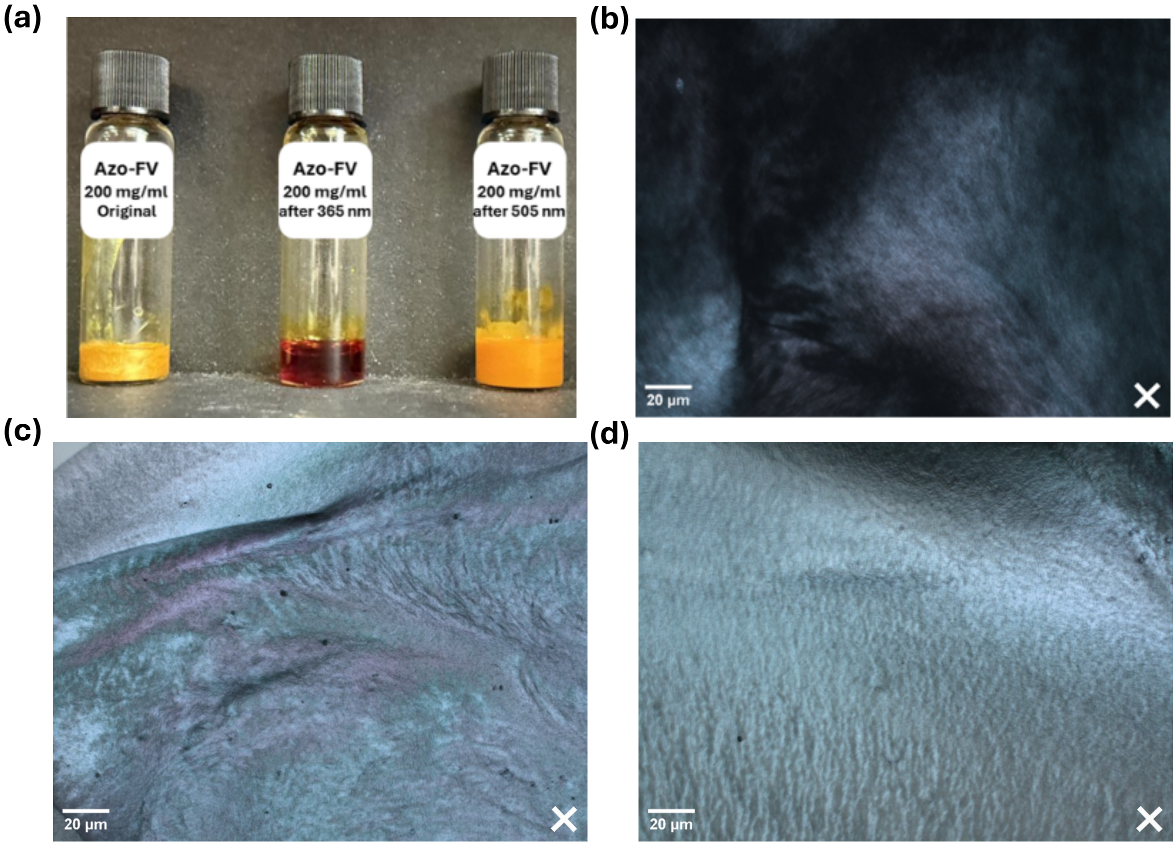

Supplementary Figure 44. (a) Photographs of solutions formed from initial *trans*-isomer (left), the *cis*-isomer (middle) and the *trans*-isomer after reversal (right), all at 200 mg/mL; Cross-polarized microscopy images of *trans*-isomer (b), the *cis*-isomer (c) and the *trans*-isomer after reversal (d), all at 200 mg/mL. Scale bar represents 20 μm and white crosses represent polariser directions.

Long term studies

Azo-FV 200 mg/mL *trans*-gel and *cis*-gel were stored in the dark for extended periods. At interval a month, a small amount was removed and diluted in pH 10.5 water to collect UV–vis spectra for comparison.

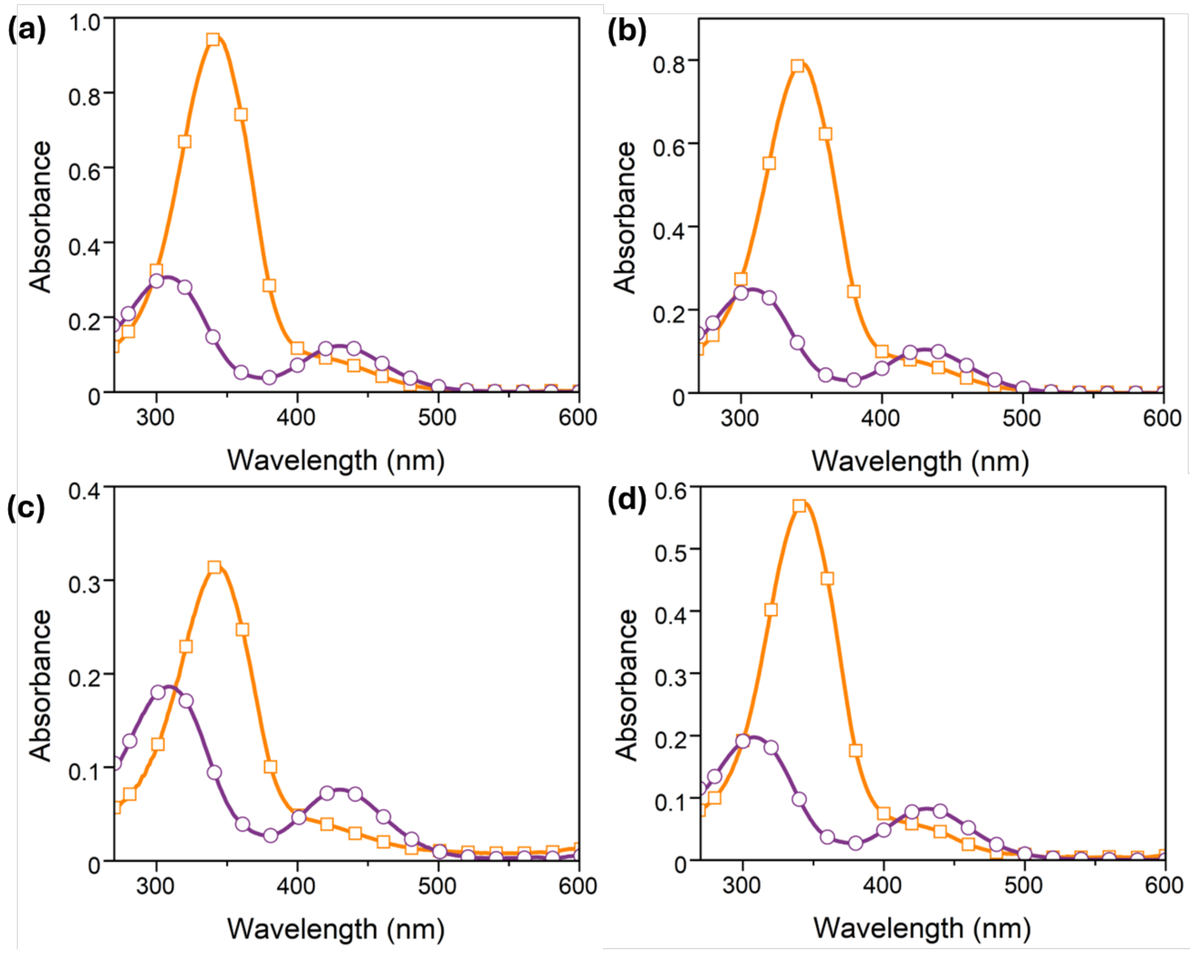

Supplementary Figure 45. UV–vis spectra for solutions of 200 mg/mL Azo-FV *trans*-gel (orange) and *cis*-gel (purple) after 0 month (a), 1 month (b), 2 months (c) and 3 months (d).

Calculation of theoretical exothermic temperature

According to the Specific heat capacity formula, $Q = mc\Delta T$, Q is heat energy, where m is the mass, c is the specific heat capacity and ΔT is the temperature change. $\Delta T = \frac{Q}{mc}$, $Q_{Azo-FV\ 200mg\cdot mol^{-1}} = 30.2\,J$ and $c_{H_2O} = 4.2\,J \cdot g°C^{-1}$. The theoretical temperature (ΔT) of water per unit volume after the complete release of heat by Azo-FV at a concentration of 200 mg/mL is calculated to be $7.19°C$.

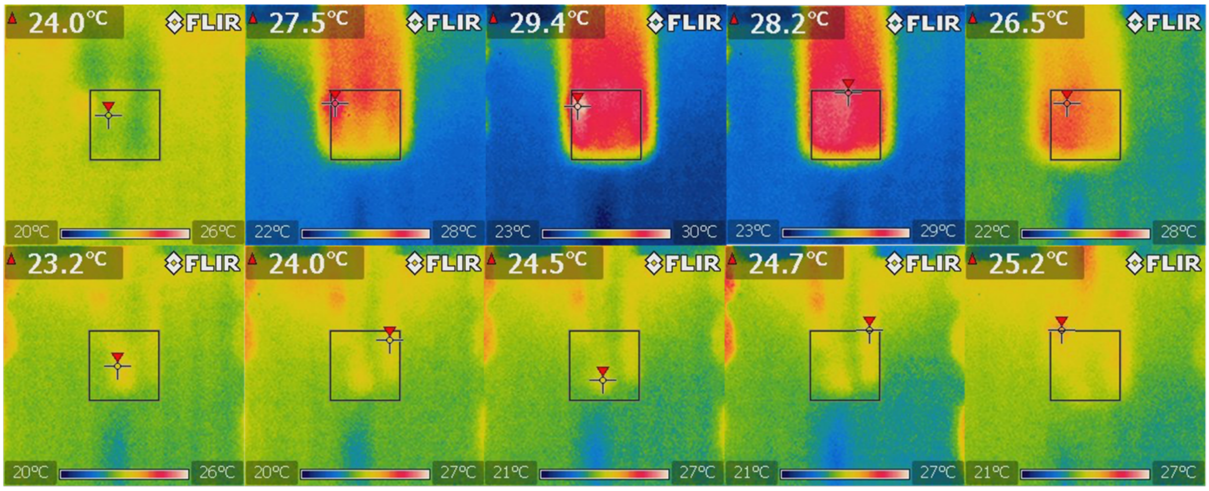

Supplementary Figure 46. Selected photographs of samples using a thermal camera used to determine data in Figure 5b; these images are expansions of those shown in Figure 5b to allow the scale bar to be seen more clearly.

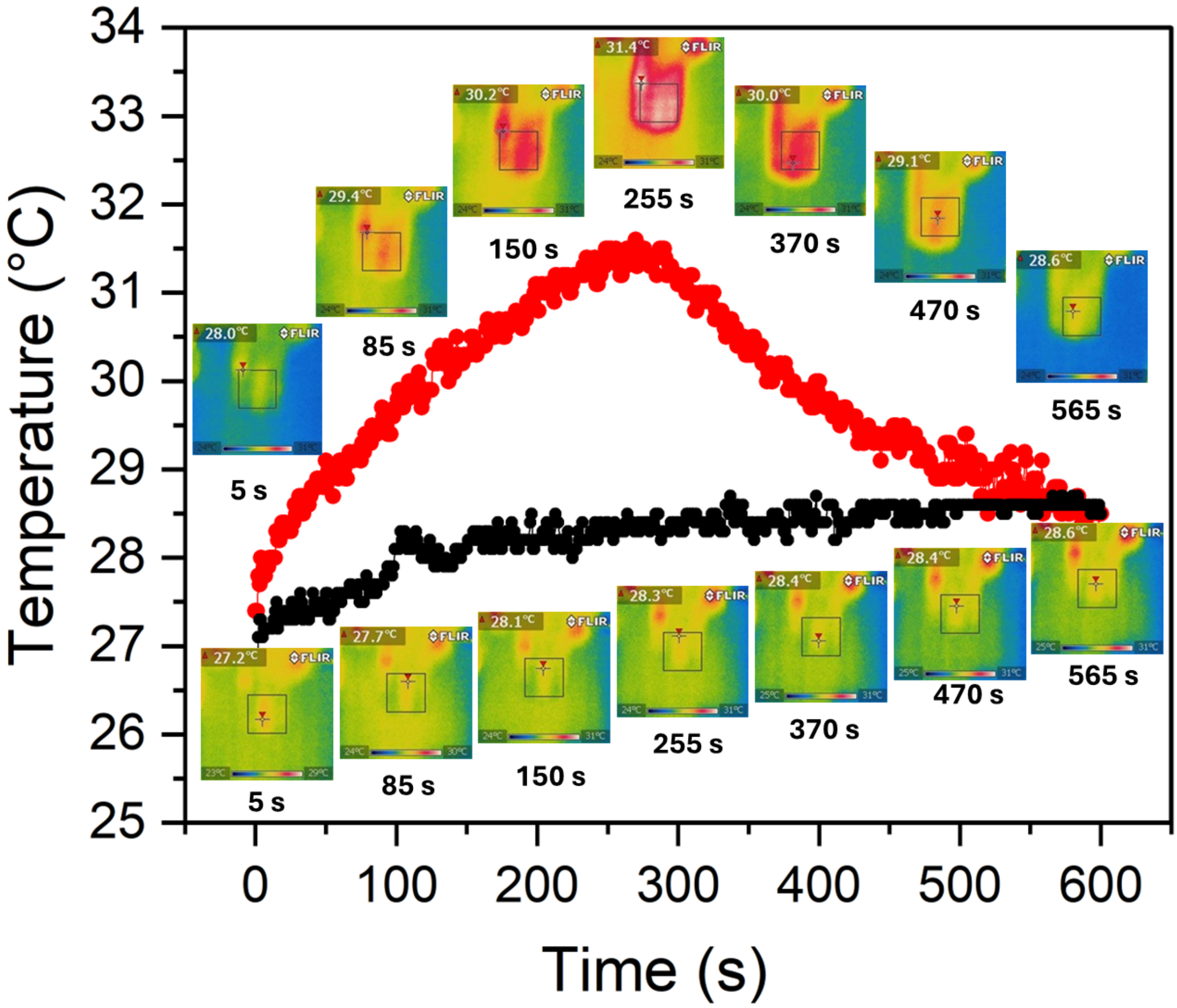

Supplementary Figure 47. Heat release from *cis*-gel (200 mg/mL) of Azo-FV (red) as compared to water (black). Here, the gel was formed and the heat release carried out immediately

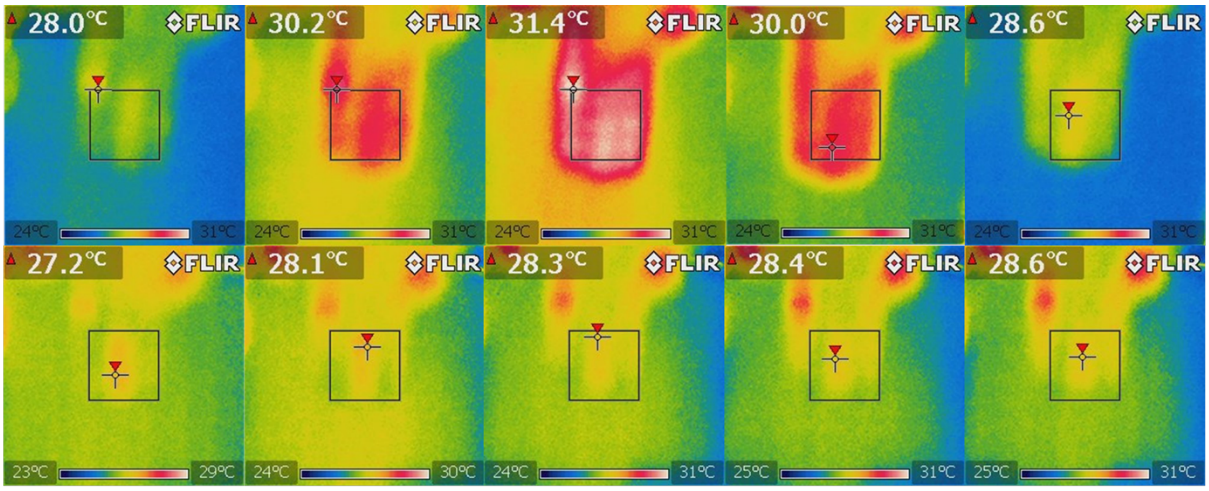

Supplementary Figure 48. Selected photographs of samples using a thermal camera used to determine data in Figure S47; these images are expansions of those shown in Figure S47 to allow the scale bar to be seen more clearly.

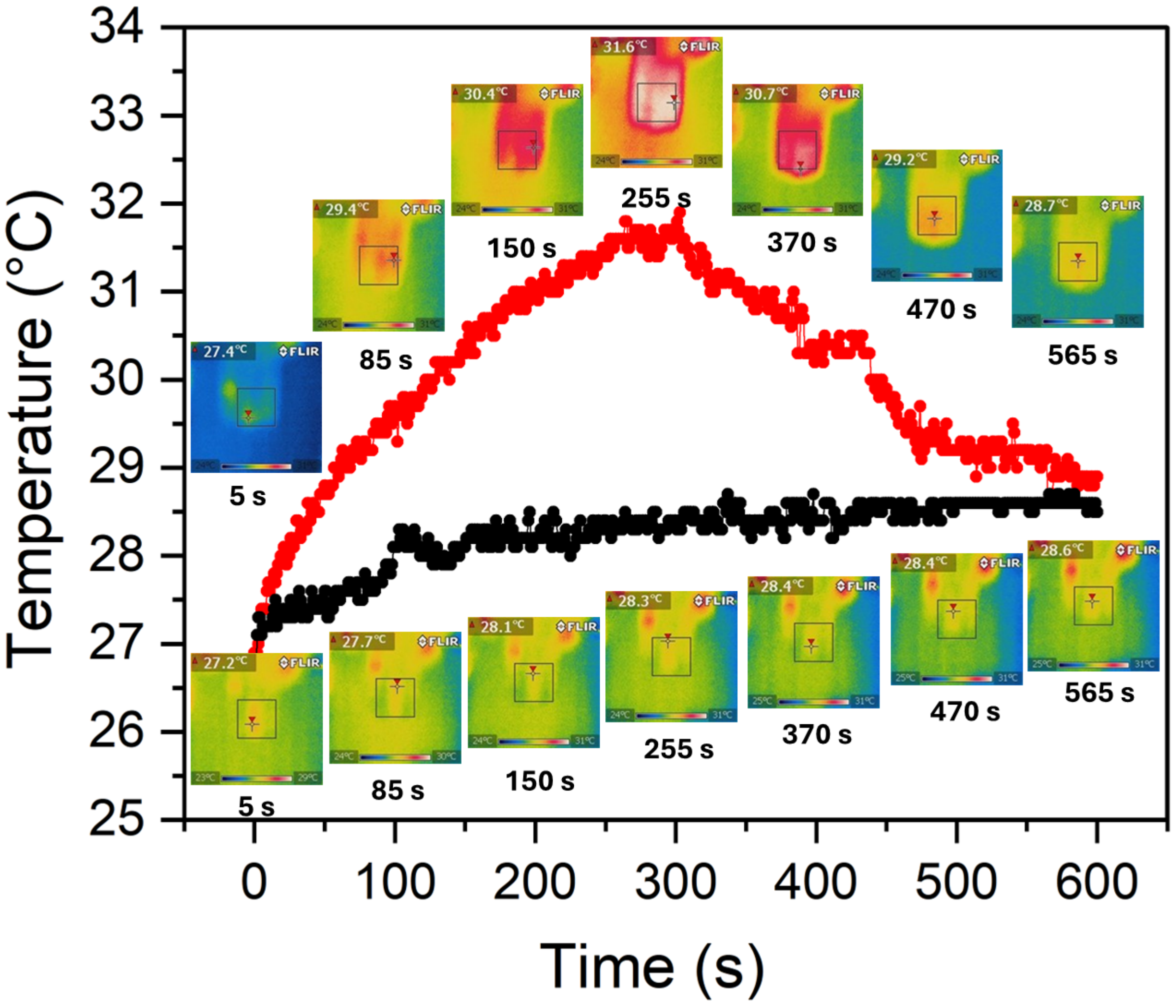

Supplementary Figure 49. Heat release from Azo-FV *cis*-solution at 200 mg/mL (red) as compared to water (black).

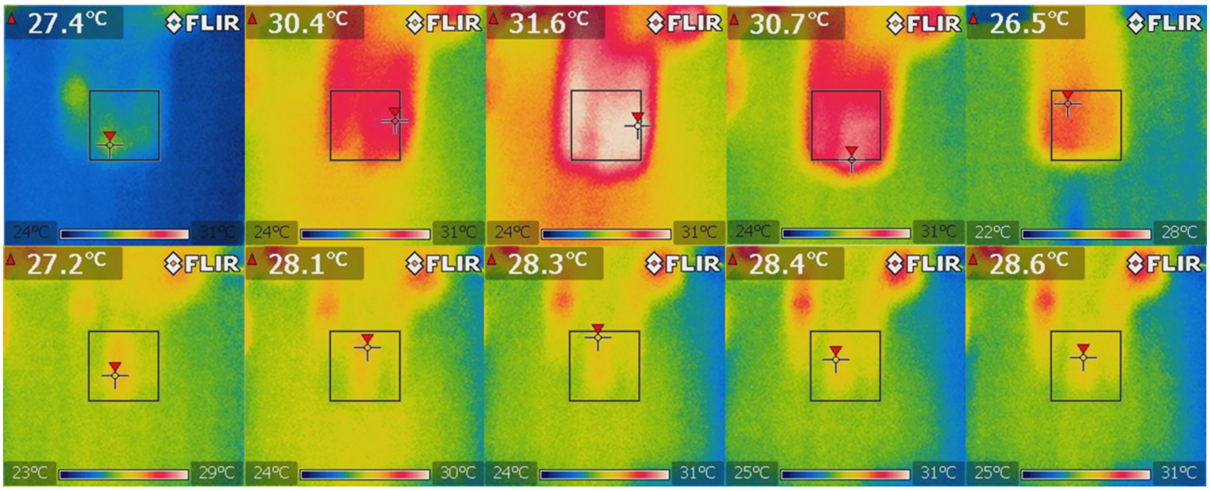

Supplementary Figure 50. Selected photographs of samples using a thermal camera used to determine data in Figure S49; these images are expansions of those shown in Figure S49 to allow the scale bar to be seen more clearly.

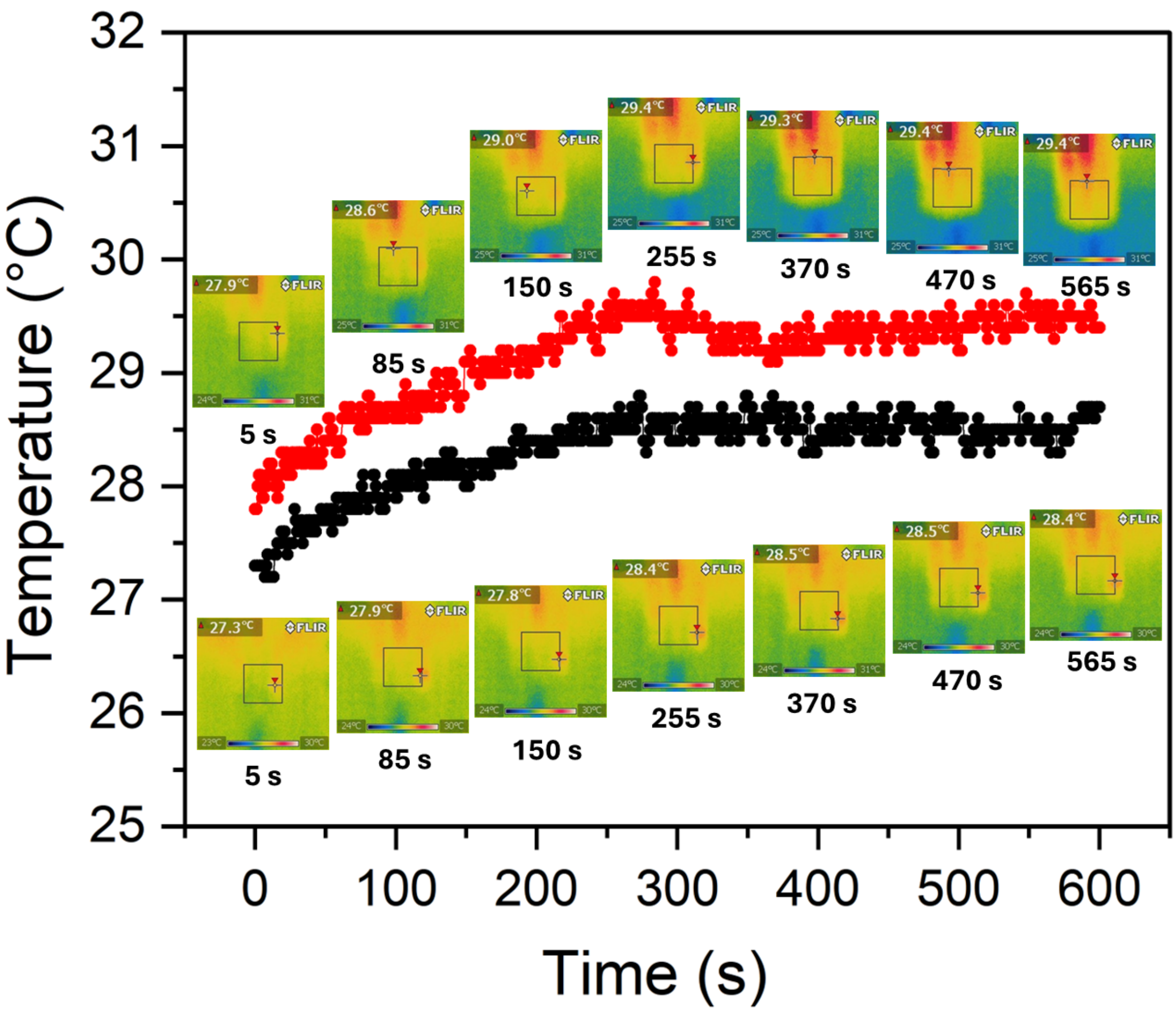

Supplementary Figure 51. Heat release controlled experiment of Azo-FV *trans*-solution at 200 mg/mL (red) as compared to water (black).

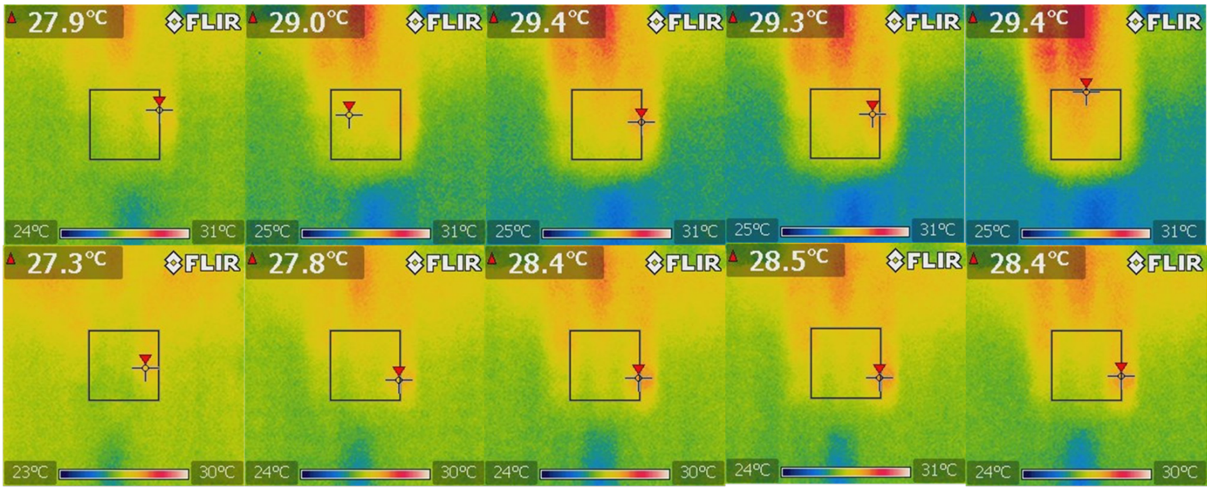

Supplementary Figure 52. Selected photographs of samples using a thermal camera used to determine data in Figure S51; these images are expansions of those shown in Figure S51 to allow the scale bar to be seen more clearly.

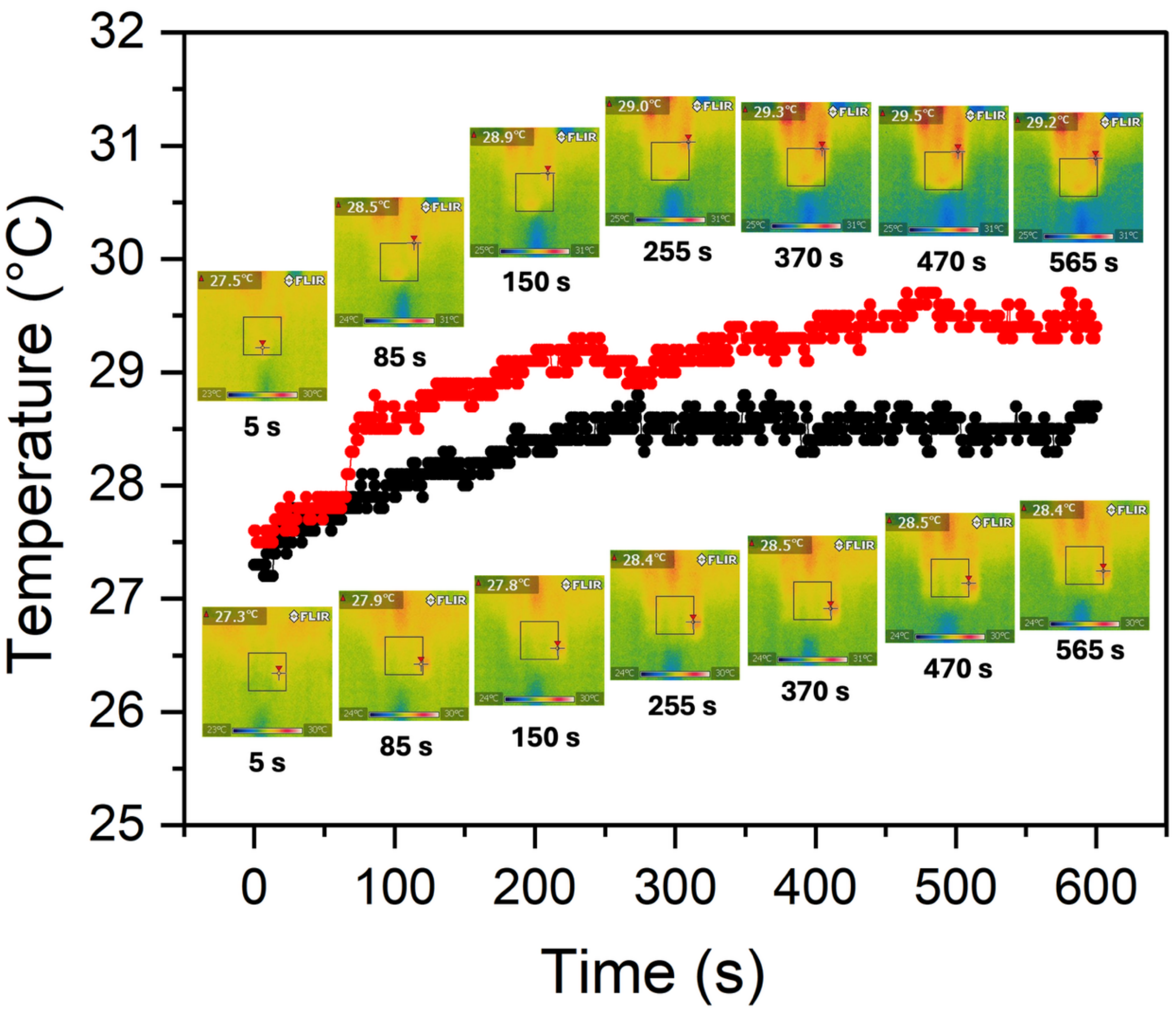

Supplementary Figure 53. Heat release from *trans*-gel (200 mg/mL) of Azo-FV (red) as compared to water (black).

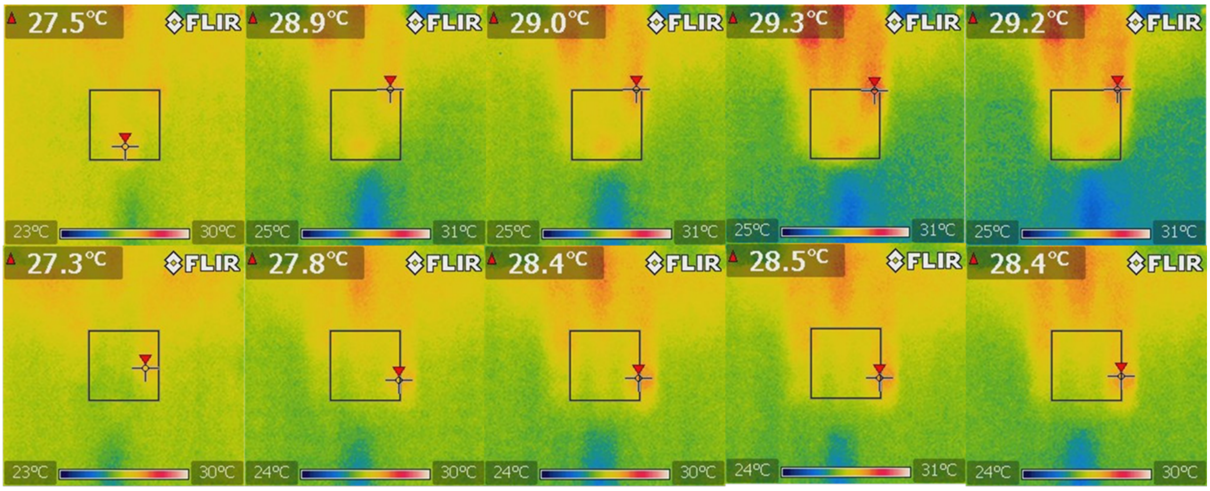

Supplementary Figure 54. Selected photographs of samples using a thermal camera used to determine data in Figure S53; these images are expansions of those shown in Figure S53 to allow the scale bar to be seen more clearly.

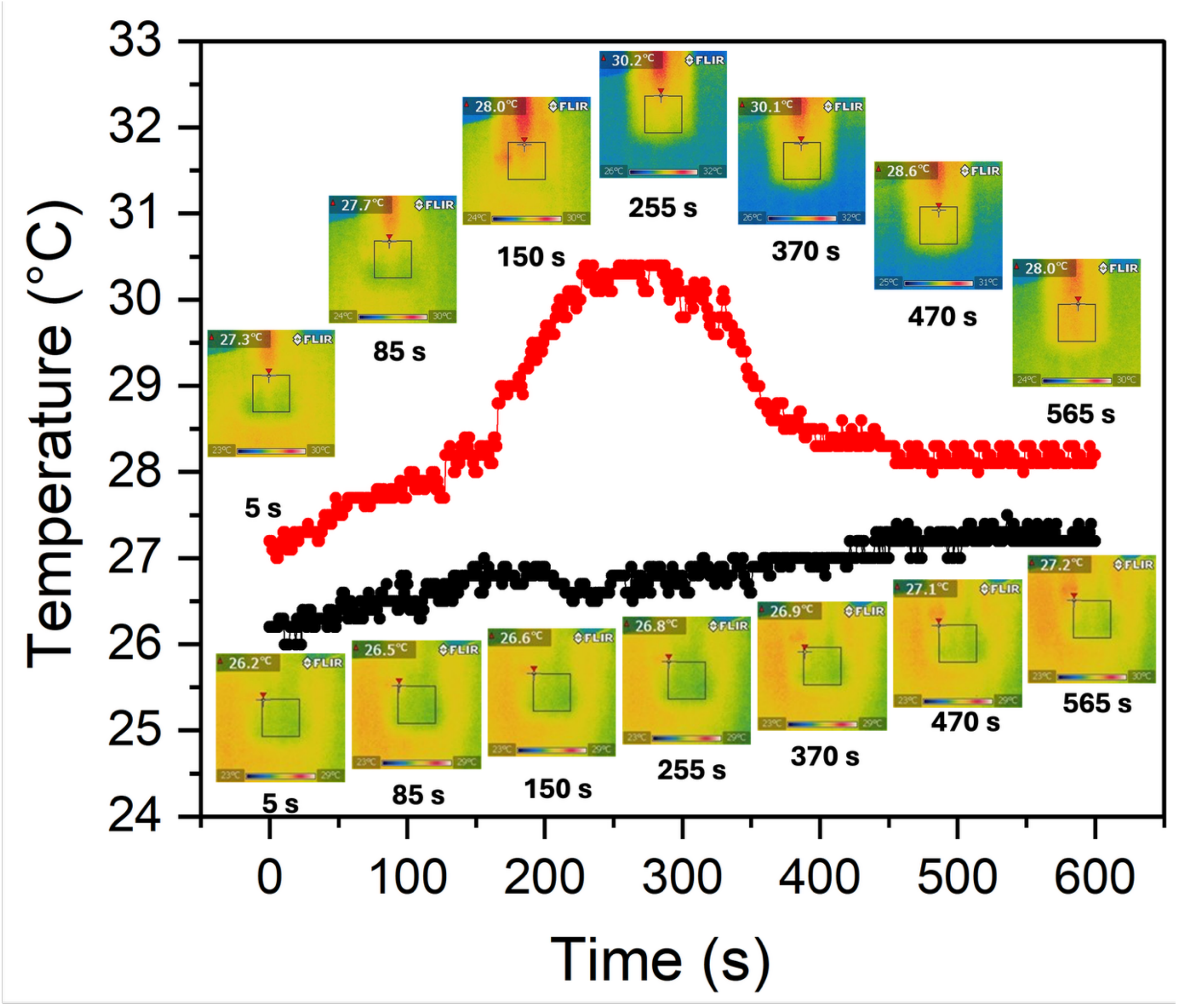

Supplementary Figure 55. Heat release from *cis*-gel (100 mg/mL) of Azo-FV (red) as compared to water (black).

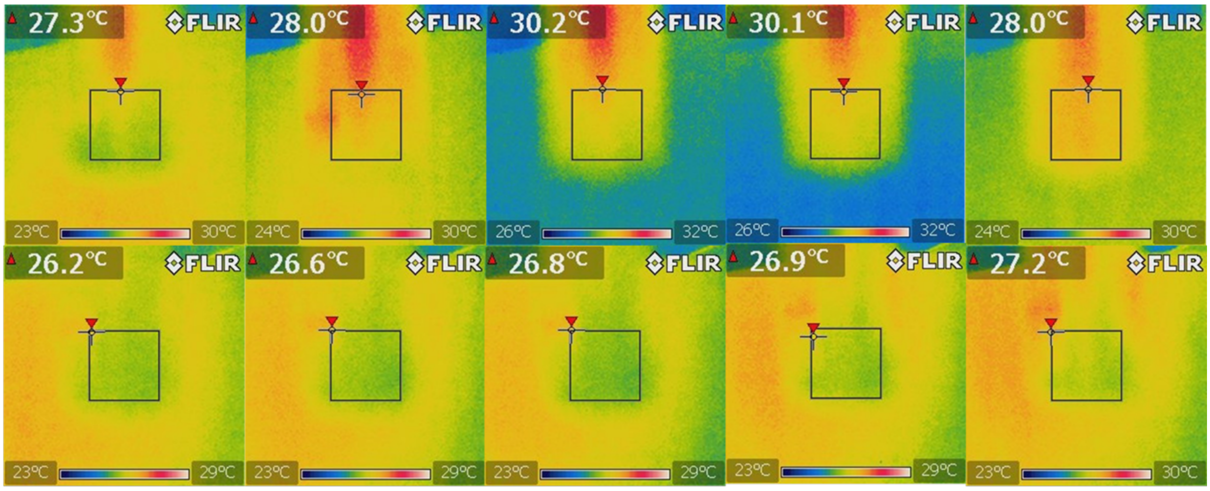

Supplementary Figure 56. Selected photographs of samples using a thermal camera used to determine data in Figure S55; these images are expansions of those shown in Figure S55 to allow the scale bar to be seen more clearly.

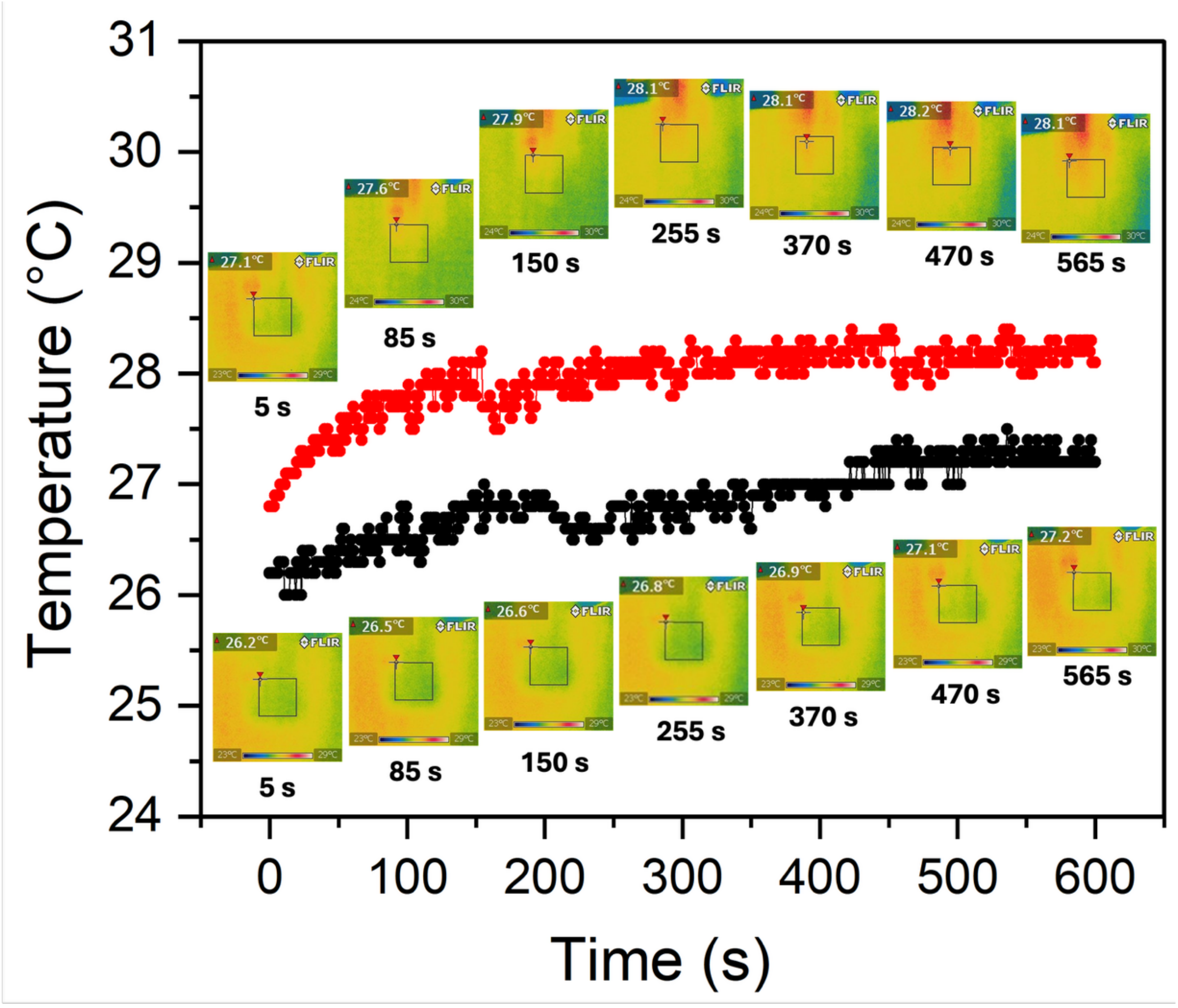

Supplementary Figure 57. Heat release controlled experiment of Azo-FV *trans*-gel at 100 mg/mL (red) as compared to water (black).

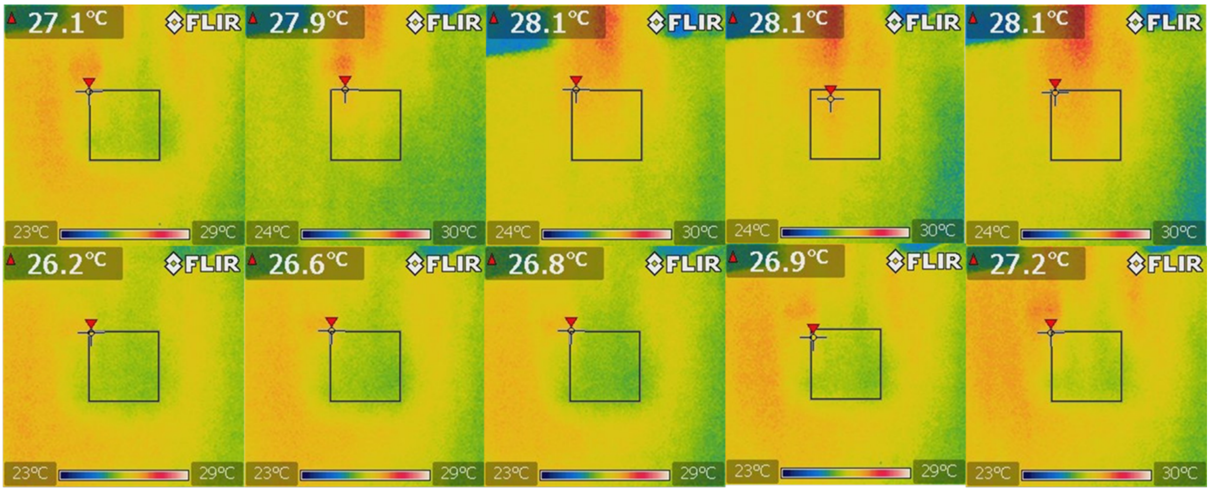

Supplementary Figure 58. Selected photographs of samples using a thermal camera used to determine data in Figure S57; these images are expansions of those shown in Figure S57 to allow the scale bar to be seen more clearly.

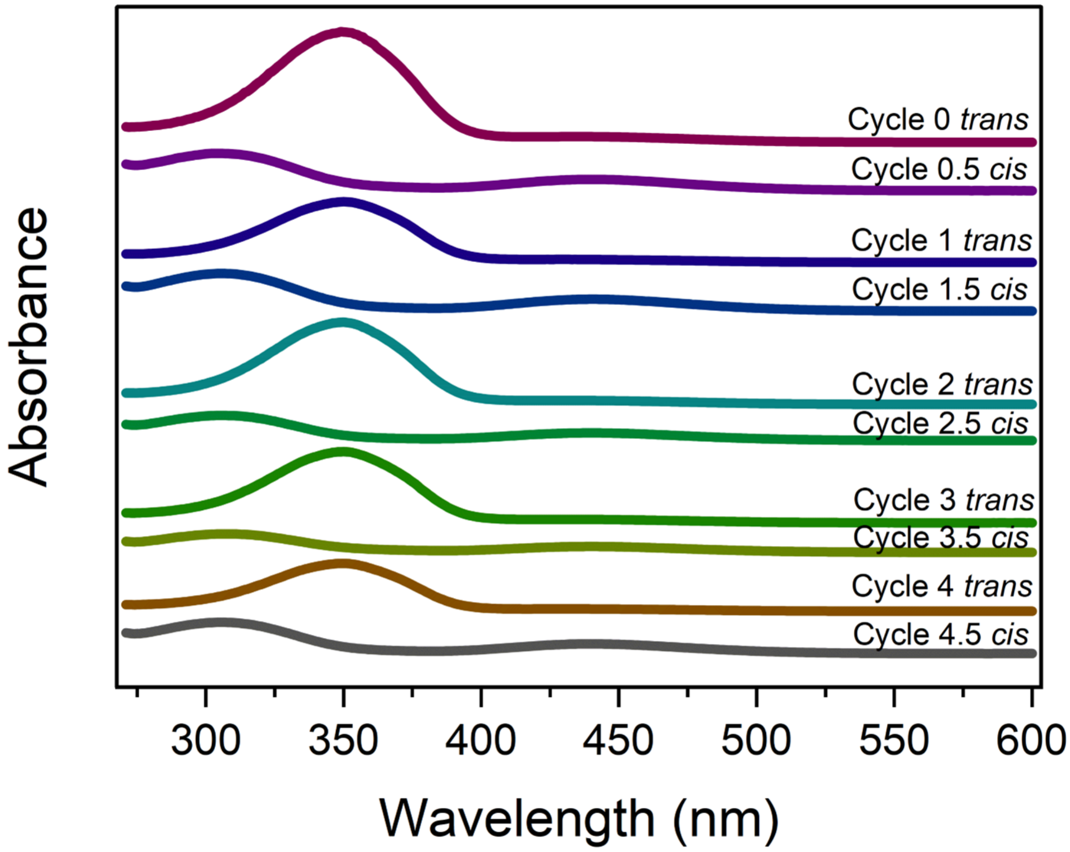

Supplementary Figure 59. UV-Vis spectrum for a small sample removed from the gel at each point in the cycling experiment correlating with the ^{1}H NMR spectra shown in Figure 5c.

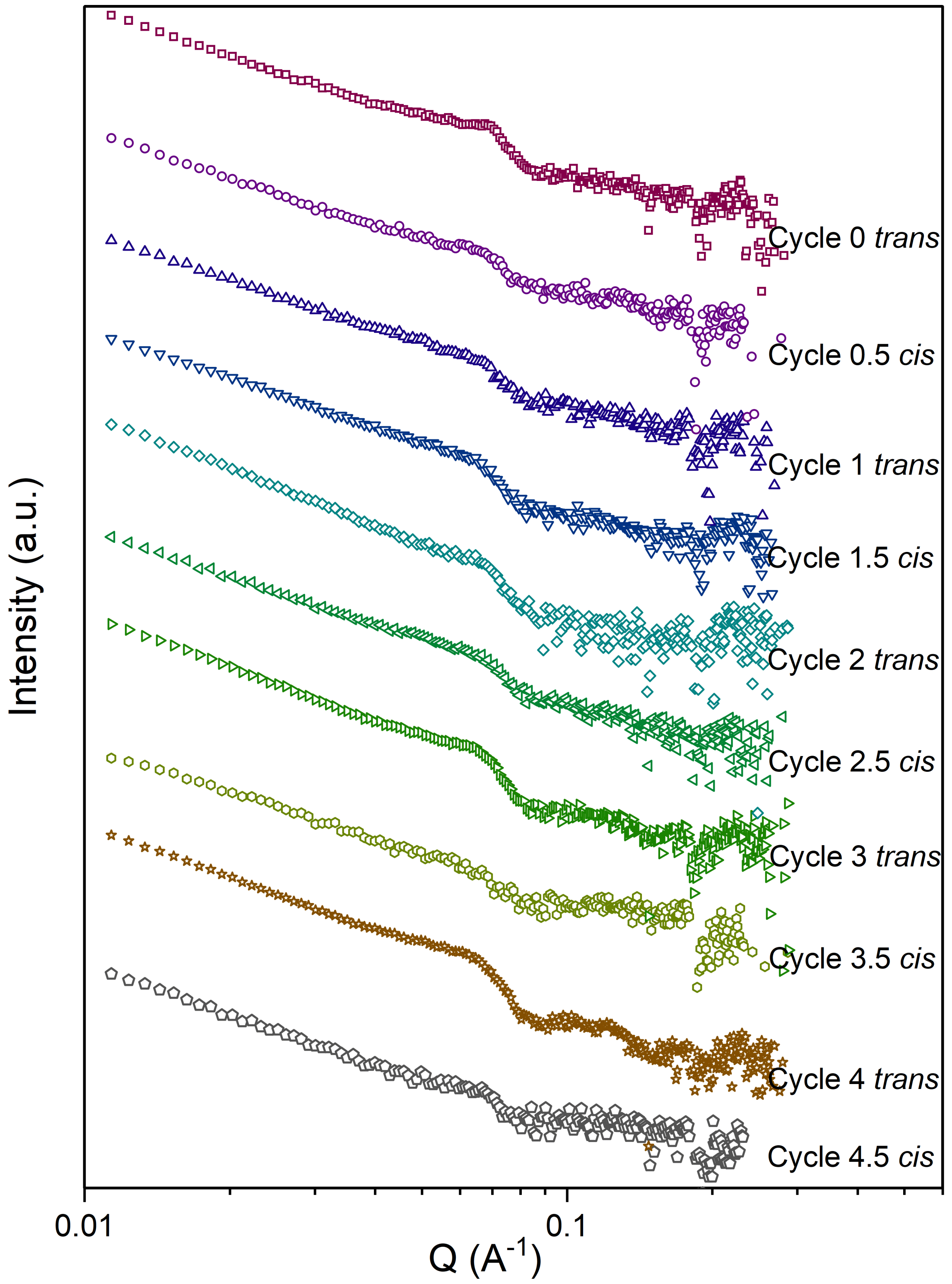

Supplementary Figure 60. SAXS data for the cycling study. Samples were prepared and irradiated with different LEDs to correlate with the heat release experiments shown in Figure 5. Note that these samples were prepared independently and not removed from the gel as were the samples for 1H NMR at UV-Vis studies (Figure 5c and Figure S59 respectively).

Supplementary References

S1. K. Haghbeen and E. W. Tan, *The Journal of Organic Chemistry*, 1998, **63**, 4503–4505.

S2. Z. Wang, B. E. Jones, L. G. Franca, T. Lawson, M. Jevric, K. Moth–Poulsen and R. C. Evans, *Journal of Materials Chemistry C*, 2024, **12**, 19030–19034.
