# Supplementary material for: Functionalized azobenzenes for micellar solar thermal energy storage as a next-generation MOST system
Source: Commun Chem. 2025 Nov 24;8:369. doi: 10.1038/s42004-025-01750-5 (PMC12644887; doi:10.1038/s42004-025-01750-5)
Supplement: Supplementary file 2 — Description of Additional Supplementary Files [file 42004_2025_1750_MOESM2_ESM.pdf]

Description of Additional Supplementary Files

File name- Supplementary Data 1

File description – Data underlying the graphs.
